# Targeting MDM4 as a Novel Therapeutic Approach in Prostate Cancer Independent of p53 Status

**DOI:** 10.3390/cancers14163947

**Published:** 2022-08-16

**Authors:** Javier Octavio Mejía-Hernández, Dinesh Raghu, Franco Caramia, Nicholas Clemons, Kenji Fujihara, Thomas Riseborough, Amina Teunisse, Aart G. Jochemsen, Lars Abrahmsén, Giovanni Blandino, Andrea Russo, Cristina Gamell, Stephen B. Fox, Catherine Mitchell, Elena A. Takano, David Byrne, Panimaya Jeffreena Miranda, Reem Saleh, Heather Thorne, Shahneen Sandhu, Scott G. Williams, Simon P. Keam, Ygal Haupt, Sue Haupt

**Affiliations:** 1 Peter MacCallum Cancer Centre, 305 Grattan St, Melbourne, VIC 3000, Australia; 2 Tumour Suppression and Cancer Sex Disparity Laboratory, Peter MacCallum Cancer Centre, Melbourne, VIC 3000, Australia; 3 Sir Peter MacCallum Department of Oncology, The University of Melbourne, Parkville, VIC 3010, Australia; 4 Olivia Newton-John Cancer Research Institute, School of Cancer Medicine, La Trobe University, Heidelberg, VIC 3084, Australia; 5 Department of Cell and Chemical Biology, Leiden University Medical Centre, 2333 Leiden, The Netherlands; 6 Aprea Therapeutics AB, 17165 Stockholm, Sweden; 7 Translational Oncology Research Unit, IRCSS Regina Elena National Cancer Institute, 0144 Rome, Italy; 8 Surgical Pathology Unit, Department of Research, Advanced Diagnostics and Technological Innovation, IRCSS Regina Elena National Cancer Institute, 0144 Rome, Italy; 9 Pathology Department, Peter MacCallum Cancer Centre, Parkville, VIC 3000, Australia; 10 Department of Medical Oncology, Peter MacCallum Cancer Centre, Parkville, VIC 3000, Australia; 11 Division of Radiation Oncology, Peter MacCallum Cancer Centre, Parkville, VIC 3000, Australia

**Keywords:** MDM4, MDMX, TP53, p53, prostate cancer, eprenetapopt, APR-246, XI-011, mutant p53

## Abstract

**Simple Summary:**

Prostate cancer is the most prevalent male cancer. It poses a survival risk if it spreads and fails treatment. In search of fresh insight into lethal prostate cancers, we examined failures in cancer defence systems operated by the key anticancer protein p53. Normally, levels of p53 activity are kept low by the protein MDM4, and consistently, we found that high MDM4 levels pose a prostate cancer risk in this context. Outside this explanation, we discovered that high MDM4 levels are also a cancer risk when p53 is genetically altered and unable to fight cancer, or even mutated to drive cancer spread. Our novel findings uncovered MDM4 inhibition as a new concept for prostate cancer treatment, and we demonstrated efficacy and uncovered mechanisms of action. Importantly, we showed that targeting MDM4 halted the growth of aggressive prostate cancer cells with mutant p53, and this was potentiated by a drug clinically trialled to target mutant p53 cancers.

**Abstract:**

Metastatic prostate cancer is a lethal disease in patients incapable of responding to therapeutic interventions. Invasive prostate cancer spread is caused by failure of the normal anti-cancer defense systems that are controlled by the tumour suppressor protein, p53. Upon mutation, p53 malfunctions. Therapeutic strategies to directly re-empower the growth-restrictive capacities of p53 in cancers have largely been unsuccessful, frequently because of a failure to discriminate responses in diseased and healthy tissues. Our studies sought alternative prostate cancer drivers, intending to uncover new treatment targets. We discovered the oncogenic potency of MDM4 in prostate cancer cells, both in the presence and absence of p53 and also its mutation. We uncovered that sustained depletion of MDM4 is growth inhibitory in prostate cancer cells, involving either apoptosis or senescence, depending on the cell and genetic context. We identified that the potency of MDM4 targeting could be potentiated in prostate cancers with mutant p53 through the addition of a first-in-class small molecule drug that was selected as a p53 reactivator and has the capacity to elevate oxidative stress in cancer cells to drive their death.

## 1. Introduction

Prostate cancer (PC) is the most frequently diagnosed malignancy and the second leading cause of cancer death in men in western countries. When detected early, localised PC is often cured; however, one in ten of these patients will subsequently develop incurable metastatic disease [1]. Methods for identifying men at higher risk of recurrence have been largely limited to histopathological factors. Guided by these, treatment intensification to improve survival outcomes after surgery and/or radiotherapy for localized disease, has been disappointing. A more recent rational strategy for novel therapeutic intervention in men at high-risk of lethal PC is to identify molecular biomarkers that correlate to actionable targets. The efficacy of this approach is well exemplified by extended patient survival in response to PARP inhibitors administered to treat men with advanced PCs harbouring BRCA1/2 alterations [2].

Metastatic progression is driven by the mutated form of the tumour-suppressor protein p53 (encoded in the human *TP53* gene), as demonstrated in mice [3,4], human breast [5] and pancreatic cancers [6]. This phenotype is one of the major, acquired neomorphic oncogenic capabilities of mutant p53, commonly referred to as ‘gain-of-function’ (GOF). Stabilisation of mutant p53 protein is essential for activation of its GOFs, and coincides with a loss of wild type p53 (wt p53) tumour-suppressive properties (as reviewed [7]). A number of key studies have overturned the earlier notion that p53 was not involved in PC and instead, demonstrated that *TP53* mutations are among the most common genetic alterations in PC metastases [8,9,10]. In the majority of cases that eventually advance to deadly PC, *TP53* mutations were detected at low frequency in the primary tumours (e.g., [11]). Importantly, clustering of mutant p53-positive cells in the primary tumour is a powerful, clinically relevant prognostic biomarker for lethal metastatic prostate cancer [12]. As high as 73% of patients with metastatic PC have mutant p53 (as reviewed [13]).

The efficacy of restoring the tumour-suppressor activities of wt p53 to eliminate tumour growth has been demonstrated (e.g., [14]). This has been achieved by blocking MDM2 and MDM4, which are two key inhibitors of p53 (as reviewed [15,16]). MDM2 drives p53 degradation [17,18] and operates in a regulatory feed-back loop with p53. MDM4 inhibits p53 transcriptional activities and also cooperates with MDM2 to degrade p53 (as reviewed [15,19]). The MDM proteins keep the growth-inhibitory activities of wt p53 in-check. Hence, deregulated high levels of either MDM proteins pose a serious cancer risk. This has attracted the pharmaceutical industry to develop inhibitors that protect p53 from the MDM family, centered primarily on MDM2. Clinical trials demonstrated MDM2 inhibitors to be highly effective, but their toxic, on-target effects on normal tissues have hampered their development (as reviewed [20]). Alternatively, MDM4 may provide a more attractive therapeutic target for the following reasons. First, by contrast with MDM2, loss of MDM4 is well tolerated by normal adult tissues [21]. Second, depletion of MDM4 inhibits the growth of wt p53 cancer cells (e.g., [19,22,23]). MDM4 inhibitors are in their infancy and are yet to be comprehensively clinically trialed [24]. Importantly, we discovered that targeting MDM4 also inhibits tumours with mutant p53, at least in breast cancer cells, challenging prevalent dogma [25].

In the current study we examined the role of MDM4 in PC and unveiled the therapeutic potential of targeting MDM4 to treat lethal PCs, either encoding wt or mutant p53, or lacking p53. We found that MDM4 is highly expressed in PC patient datasets, in particular in metastatic tumours. Our study uncovered a critical role for MDM4 in the growth and survival of PCs in vitro and in vivo. Importantly, we demonstrated that MDM4 depletion is inhibitory not only to PC cells expressing wt p53, but also to those lacking p53 or expressing missense mutant p53. We showed that PC inhibition caused by depleting MDM4 can be potentiated in cancers harbouring mutant p53, by co-treatment with eprenetapopt (APR-246), a first-in-class drug originally screened for its capacity to target mutant p53 cancers.

## 2. Materials and Methods

### 2.1. Prostate Cancer Specimens

Studies of autopsied human specimens were approved by the Peter MacCallum Cancer Centre Human Ethics Committee. Tissue microarray (TMA) PC biopsies were collected from the Urology Department at IRCCS Regina Elena National Cancer Institute, Rome, Italy.

### 2.2. Immunohistochemistry

Tumour samples and TMAs were stained using anti-MDM4 (A300 287A; Bethyl Laboratories), anti-MDM2 (C-18 Rabbit polyclonal; Santa Cruz Biotechnology) and anti-p53 (Mouse monoclonal D-O7; Novocastra, Leica Biosystems) antibodies. Samples were scored for both staining intensity and the proportion of cells stained. Intensity of nuclear staining was scored as either 0 (absent), 1 (low), 2 (intermediate) or 3 (strong), and the proportion of tumour cells stained was then categorised according to percentage staining 0, 1 => 25%, 2 => 25–50%, 3 => 50–75% and 4 => 75%. The histoscores were calculated as the product of the intensity added to the proportion of stained tumour cells, on a scale between 0–7.

### 2.3. Cell Culture

Cell lines were purchased from ATCC (ATCC, Manassas, VA, USA). All cell lines were maintained at 5% CO_2_ in culture medium with 10% fetal bovine serum (Cat. 10082147, Gibco, Big Cabin, OK, USA) and 0.1% *w*/*v* penicillin/streptomycin (Cat. 10378016, Gibco). HEK293, SKBr3, DU145 (p53^P223L/V274F^) PC-3 (p53^null^) and PC-3 (p53^R273H^) were maintained in Dulbecco’s Modified Eagle’s Medium (DMEM) (Cat. 12491015, Gibco). C4-2 (p53^wt/wt^) cells were maintained in RPMI-1640 (Cat. 11875093, ThermoFisher, New York, NY, USA); 22Rv1 (p53^wt/Q331R^) were maintained in RPMI-1640 (Cat. 11875093, ThermoFisher) containing 0.25% glucose (Cat. G7021, Sigma-Aldrich, St. Louis, MO, USA), 1 mM sodium pyruvate (Cat. S8636, Sigma-Aldrich), 0.1 mM HEPES buffer (Cat. H0887, Sigma-Aldrich). VCaP (p53^A248W/null^) were cultured in DMEM containing 1 mM sodium pyruvate (Cat. S8636, Sigma-Aldrich) and 1.5 g/L sodium bicarbonate (Cat. S5761, Sigma-Aldrich). For passaging, cells were carefully washed using phosphate buffer saline (PBS) buffer solution (1X) and detached using 0.25% trypsin (Cat. 25200072, Gibco). PCR was used to confirm that the cells were clear of mycoplasma at regular intervals.

### 2.4. Inducible Lentiviral Short Hairpin RNA (shRNA) Sequences and Viral Production

Viruses were generated using HEK293 cells as described by Herold et al., 2008 [26]. Lentivirus was generated for the transduction of cell lines and contained a FH1t vector constitutively expressing a green fluorescent protein (GFP) tag as previously described [19,25,26], and a Doxycycline-inducible shMDM4 (shRNA targeting MDM4) and shCtrl (shRNA control). All PC cell lines were transduced as described by Miranda et al., 2017 [25]. GFP^+^ cells were subsequently sorted using BD FUSION flow cytometer 5 (BD Biosciences). Untransduced PC cell lines were used as negative controls for gating. In vitro shRNA expression was induced using 25 ng/mL of Doxycycline (Doxy), with replenishment every 2 days, without media change, for the duration of each experiment. Specifically, for in vitro shRNA expression, PC cell lines were seeded in complete media containing 25 ng/mL of Doxy, with addition every 2 days to maintain this concentration. Media changes were avoided by adding minimal volumes. A working Doxy stock solution was prepared accordingly, depending on the final volume of the plates/wells by diluting a 10 mg/mL stock in PBS. Verified shRNA sequences are as previously reported (using Forward: F; Reverse: R primers): Targeting Human MDM4 Exon 7, shMDM4 F-TCCCGTGCAGAGGAAAGTTCCACTTCAAGAGAGTGGAACTTTCCTCTGCACTTTTTC; shMDM4 R-TCGAGAAAAAGTGCAGAGGAAAGTTCCACTCTCTTGAAGTGGAACTTTCCTCTGCAC; Targeting Mdmx (control), shCtrl F-TCCCGAATCTTGTTACATCAGCTTTCAAGAGAAGCTGATGTAACAAGATTCTTTTTC; shCtrl R-TCGAGAAAAAAGCTGATGTAACAAGATTCTCTCTTGAAACATGGCTTCAAGAGATTC [19,25].

### 2.5. Transduction of PC-3 (p53^null^) with p53-R273H

The PC-3 (p53^R273H^) cell line was generated by transducing the PC-3 (p53^null^) parental cell line using a lentivirus construct for constitutive overexpression of p53^R273H^ by cloning pLenti6/V5-p53_R273H (Cat. 22934, Addgene). PC-3 cells infected with the recombinant lentivirus were selected with 550 μg/mL of blasticidin (Cat. A1113902, Gibco, New York, NY, USA). Two PC-3 (p53^R273H^) clones were generated. Clone 2 was selected for its high expression levels of mutant p53 (Supplementary Figure S7).

### 2.6. p53 Immunofluorescence Assay of PC-3 (p53^R273H^)

PC-3 (p53^R273H^) cells were seeded on coverslips, fixed with 4% paraformaldehyde and permeabilised with 0.2% TritonX-100 (Cat. 85112, ThermoFisher). Cells were blocked with 3% BSA (Cat. 9998, Cell Signaling, Danvers, MA, USA) in PBS (1X) for 30 min and washed with PBS (1X). Cells were then incubated with murine anti-p53 antibody (D-01/1801 hybridoma, at dilution factor of 1:4), diluted in 1% BSA/PBS overnight at 4 °C in a humid chamber. The coverslips were subsequently washed and incubated with anti-mouse Alexa 488 (Cat. A32723, ThermoFisher, Hanover Park, IL, USA, at dilution of 1:700) along with DAPI (Cat. D1306, Invitrogen, Inchinnan, UK, 1:5000 from stock of 10 mg/mL) for 1 h at room temperature in a humid chamber and then mounted on glass slides with Fluorsafe mounting medium (Cat. 345789, Millipore, North Ryde, NSW, Australia). Stained cells were imaged using Olympus BX-51 (Olympus, Tokyo, Japan) and software package Spot one (version 5.0.27; Diagnostics Instruments Inc., Sterling Heights, MI, USA).

### 2.7. Incucyte^®^ Cell Count Proliferation and Caspase 3/7 Apoptosis Assays

An Incucyte^®^ Live-Cell analysis device was used to capture high-resolution temporal fluorescence and bright field images. All cell lines were seeded in 100 μL/well in 96-well flat-bottom plates (Cat. 3595, Corning, Kennebunk, ME, USA) prior to experimental treatment. The optimal seeding density for each cell line was determined prior to the commencement of experiments. Experiments were performed with six replicates. Cells were treated with either, Doxycycline [Doxy] (25 ng/mL/well; Cat. D9891, Sigma-Aldrich), doxorubicin (50 μM/well; Batch: 92094703, EBEWE Pharma, Unterach, Austria), z-VAD-FMK (25 μM/well; Cat. V116, Sigma-Aldrich), ferrostatin-1 [Fer-1] (10 μM/well; Cat. SML0583, Sigma-Aldrich), N-acetylcysteine [NAC] (2.5 mM/well; Cat. A0737, Sigma-Aldrich), ciclopirox [CPX] (0.5 μM/well; Cat. PHR1920, Supelco, Castle Hill, NSW, Australia), propidium iodide [PI] (5 μg/mL/well; Cat. P4864, Sigma-Aldrich), Incucyte^®^ Caspase-3/7 Red Dye (1:1000/well, Cat. 4440, Sartorius, New York, NY, USA) and combinations of these. The concentration of DMSO (vehicle) (Cat. D8418, Merck, Macquarie Park, NSW, Australia) was kept consistent across different drug treatments (0.25% as the final concentration per well). The cell plates were placed into the Incucyte^®^ Live-Cell Analysis System and allowed to warm to 37 °C for 30 min before scanning (objective 10X, phase contrast + green channel + red channel, standard/adherent cell-by-cell scan type). Plates were maintained at 37 °C in 5% CO_2_, and scanning was conducted periodically for seven days. Image analysis was done using the integrated software per the manufacturers’ recommendations (Incucyte^®^ Live-Cell Analysis System and Cell-by-Cell Analysis Software Module).

### 2.8. Small Molecule Preparation and Treatments

Doxycycline: Doxycycline hyclate [Doxy] (Cat. D9891, Sigma-Aldrich) was prepared as a stock of 10 mg/mL solution for in vitro experiments and diluted to a concentration of 25 ng/mL in culture medium.

APR-246 (eprenetapopt): (Syngene, batch no: S0312053) was obtained from Aprea therapeutics (Solna, Sweden). APR-246 was prepared as a 100 mM stock in 100% DMSO (Cat. D8418, Merck) and stored in aliquots at −20 °C for in vitro and in vivo experiments. For in vitro experiments, stock solutions were diluted in complete media to reach the desired final concentrations of APR-246. Care was taken to keep the DMSO concentration consistent across different doses and between cell lines. APR-246 dose–response curves were determined using a range of concentrations (0–60 µM) and two biochemical assays (SRB and Alamar Blue assays). For in vivo experiments, the APR-246 stock solution (100 mM) was diluted to a 120 mg/mL working concentration using PBS. The working stock was aliquoted and stored at −20 °C. Before administering to the mice, the working stock was further diluted in PBS for IP injection as 200 mg/Kg/day per mouse for a period of 14 days.

XI-011: The MDM4 inhibitor XI-011 (Cat. AOB1263, AOBIOUS, Gloucester, MA, USA) was prepared in DMSO (Cat. D8418, Merck) at 4 mg/mL for in vitro studies (as previously described [27]).

### 2.9. Colourimetric Assays

Cell viability was determined after drug treatment using either Alamar Blue (AB), Sulforhodamine B (SRB) assays as previously described [25,28,29] or CellTiter-Blue^®^ Cell Viability assay (Cat. G8080, Promega, Madison, WI, USA for VCap alone).

Senescence-associated β-galactosidase (SA-β-gal) staining was performed as described by Dimri et al., 1995 [30]. Cell plates were examined microscopically using a Zeiss Axiovert S100 Inverted Phase Contrast Microscope (Zeiss). The proportion of positive SA-β-gal cells relative to the total number of cells in three random fields of three biological replicates (per treatment group) was determined.

### 2.10. Immunoblotting

Immunoblotting was undertaken as previously described [25,31], with the following mouse primary antibodies against the following human proteins: p53 (DO1 and 1801, a kind gift of Sir D.Lane); MDM4 (8C6 04-1555; Merck Millipore, Darmstadt, Germany); PARP-1 (33-3100; Zymed/Invitrogen^TM^, Sunnyvale, CA, USA); and p27 (610241; BD Biosciences, San Jose, CA, USA). The following rabbit primary antibodies were also used: MDM2 (C-18; Santa Cruz, Buda, TX, USA; and 3G9 04-1530 from Merck Millipore for VCap alone); Cleaved caspase 3 (9664, Cell Signalling); SKP2 (ab68455, Abcam, Cambridge, UK); p21 (12D1, Cell Signalling); SLC7A11 (D2M7A, Cell Signaling), BAX (B9; Santa Cruz, TX, USA); β-actin (13E5, Cell Signalling); USP7 (A300-034A, Bethyl Laboratories); PARP (9542; Cell Signaling Technologies, for VCap alone); p73 (A300-126; Bethyl Laboratories, Montgomery, TX, USA) and Hsp60 (H-300, Santa Cruz). Primary antibody detection was undertaken using secondary polyclonal antibodies from goats (Invitrogen) that were horse radish peroxidase (HRP) conjugated and directed against immunoglobulins of mouse or rabbit, as appropriate.

### 2.11. Flow Cytometry

DU145 adherent and non-adherent cells were harvested on day 5 after Doxycycline treatment. A total of 5 × 10^6^ cells were resuspended in 500 μL of FACS buffer (2% FBS in PBS). Live cells were distinguished from dead cells by cell dye exclusion using TO-PRO™3 dye (Cat. R37170, ThermoFisher) with FACSVERSE (BD Biosciences). The data were analysed using the Flow Logic software (Inivai Technologies, Mentone, VIC, Australia).

### 2.12. RNA Extraction and Polymerase Chain Reactions (PCRs)

RNA extraction and cDNA generation were performed as previously described [19,25] using a Promega Reverse Transcription System (Cat. A3500, Promega). The resulting cDNA was amplified with 2 μM forward (5′-3′, F) and reverse (5′-3′, R) primers and Fast SYBR™ Green (Cat. 4309155, ThermoFisher) in the Applied Biosystems StepOne Real-Time (RT) PCR system (ThermoFisher) as previously reported [19,25] using the following primer pairs: for forward primer sequence (F) and Reverse primer sequence (R): **hMDM4 (HDMX)** F- TGATTGTCGAAGAACCATTTCGG, R- TGCAGGGATCAAAAAGTTTGGAG; **hBBC3 (PUMA)** F-CGACCTCAACGCACAGTACGA, R-AGGCACCTAATTGGGCTCCAT; **hPMAIP1 (NOXA)** F-ATGAATGCACCTTCACATTCCTC, R-TCCAGCAGAGCTGGAAGTCGAGTGT; **hBAX (BCL2L4)** F-GGACGAACTGGACAGTAACATGG, R-GCAAAGTAGAAAAGGGCGACAAC; **hCCNA2 (Cyclin A2)** F-AGCAGCCTGCAAACTGCAAAGTTG, R-TGGTGGGTTGAGGAGAGAAACAC; **hCDKN2A (p16INK4a)** F-ATGGAGCCTTCGGCTGACT, R-GTAACTATTCGGTGCGTTGGG; **hSERPINE1 (PAI-1)** F-CCGGAACAGCCTGAAGAAGTG, R-GTGTTTCAGCAGGTGGCGC; **hCDKN1A (p21Cip1/Waf1)** F-GAGGCCGGGATGAGTTGGGAGGAG, R-CAGCCGGCGTTTGGAGTGGTAGAA; **hCDKN1B (p27Kip1)** F-GGCCTCAGAAGACGTCAAAC, R-ACAGGATGTCCATTCCATGA; **hSKP2 (FBXL1)** F-GATGTGACTGGTCGGTTGCTGT, R-GAGTTCGATAGGTCCATGTGCTG; **hRPL37a** F-GCCAGCACGCCAAGTACAC, R-CCCCACAGCTCGTCTCTTCA [19]. Primers targeting RPL37a were used as a loading control. Relative gene expression was calculated using the 2^−ΔΔCT^ method as described by Livak et al., 2001 [32]. All primers were purchased from Sigma-Aldrich (MO, USA).

### 2.13. In Vivo Mice Experiments

Mice were cared for and treated according to the Australian Code of Animal Care and the Use of Animals for Scientific Purpose. Mouse experiments were conducted with approval from the Peter MacCallum Cancer Centre Animal Experimentation Ethics Committee. Mice were housed in a pathogen-free environment, in a 12 h light/dark cycle at 23 °C ± 2 °C room temperature with a relative humidity of 50 ± 20% and ad libitum access to food and water.

In preparation for mice prophylaxis in vivo experiments, DU145 cells were pre-treated with Doxycycline for 24 h prior to subcutaneous injection into the contralateral flanks of NOD SCID gamma IL2R-gamma chain (NSG) male mice at 6–8 weeks of age, as previously described [33]. Per side, 0.5 × 10^6^ cells/100 µL Matrigel (Cat. 354234, BD Biosciences) were injected into recipient mice (*n* = 6 per cohort) that had been pre-primed with Doxycycline, which was continuously supplied ad libitum in the drinking water (2.0 mg/L) until ethical endpoint, as previously described [25].

In preparation for the mice treatment experiment DU145 and PC-3 (p53^R273H^) cells were injected as just described, but without prior Doxycycline treatment. Tumours were allowed to grow to ~150 mm^3^. Doxycycline was then supplied in the drinking water (2.0 mg/L) and maintained until endpoint. Three days after commencing Doxycycline administration, eprenetapopt was administered at 200 mg/Kg per day by intraperitoneal injection (100 mg/Kg twice a day) for 14 days (which was the limit approved by our animal ethics committee). Tumour growth was measured (volume = [length × width × width]/2) weekly at a minimum using vernier calipers until the endpoint was reached (tumour volume of ~1500 mm^3^), at which point the mice were culled as per ethics guidelines.

### 2.14. Statistical Analyses

Two-way comparisons were analysed using an unpaired and two-tailed Student’s *t*-test using Prism 9 (GraphPad). One-way analysis of variance (ANOVA) followed by a Tukey post-hoc comparison test was performed on comparisons of more than two conditions using Prism 9 (GraphPad). Data are presented if not indicated elsewhere as mean ± standard error of the mean (SEM). Statistical significance is indicated in all figures.

## 3. Results

### 3.1. MDM4 Levels Are High in Primary and Metastatic PC

**Figure 1:**
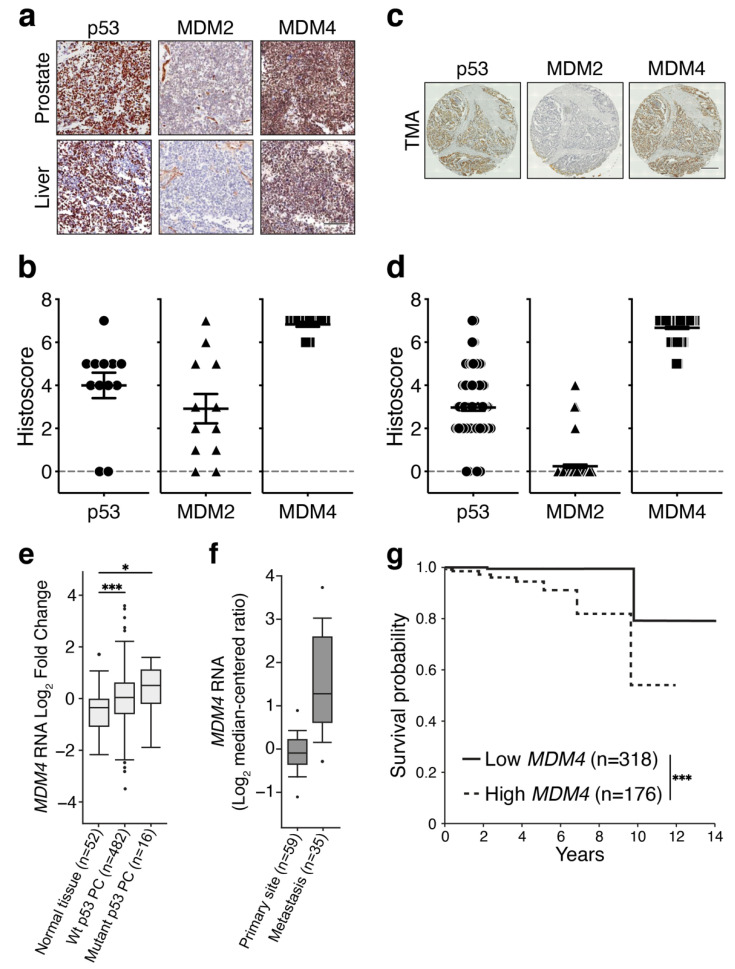
MDM4 is highly expressed in primary and metastatic prostate cancer and is associated with poor clinical outcome. (**a**,**b**) Primary and metastatic tumour samples were collected from autopsied PC patients (*n* = 5) and stained with antibodies against p53, MDM2, and MDM4. (**b**) Graph showing the staining intensity and the proportion of cells stained (histoscore) of metastatic samples (*n* = 12, see Supplementary Table S1). (**c**,**d**) TMA of a second cohort of primary PC samples (*n* = 120) [Gleason grade 6–9 and tumour stage T2–T4]. Likewise, TMAs were stained for p53, MDM2, and MDM4. (**d**) Graph shows TMAs histoscore. Histoscores were calculated as described in Materials and Methods. Scale bars are equivalent to 100 µm. Data is shown as mean ± SEM. (**e**) TCGA analysis of *MDM4* mRNA expression levels in PC that retain either wt or mutant p53 compared to normal prostate tissue. (**f**) Oncomine dataset analysis of *MDM4* mRNA expression levels in primary and metastatic PC. Data is shown as mean ± SD. Statistical significance was calculated using ANOVA and Tukey’s tests. (**g**) Kaplan–Meier plot for PC patients expressing either low or high *MDM4* mRNA levels as a function of survival probability. Statistical significance was calculated using a Log-rank (Mantel–Cox) test. Statistical significance is shown as * *p* ≤ 0.05, *** *p* ≤ 0.001.

The tumour-suppressive capacity of p53 is frequently lost in PC by direct mutation [8,10,34]. *TP53* mutation frequency increases (>70%) with progression to lethal metastatic PC (reviewed [13]). In PCs that retain wt p53, we hypothesized that its activities are compromised by deregulation of its key negative regulators, MDM2 and MDM4. Importantly though, as both MDM proteins have p53-independent oncogenic functions in various cancer types (reviewed in [16,35]), we speculated that their activities may extend beyond a wt p53 context also in PC. To define the role of the MDM proteins in PC, we examined their expression relative to p53 status at the protein and mRNA levels. For this purpose, we undertook an exploratory study on PC samples from CASCADE (Cancer tissue Collection After Death) autopsy cohort, (*n* = 5 Australian patients, each with either two or three metastases, *n* = 12 in total [36]). At autopsy, primary prostate tumour in situ was available for sampling from only two of the patients. p53, MDM2 and MDM4 proteins were detected by immunohistochemistry (IHC), as shown for a single patient, who had both primary PC and a liver metastasis (Figure 1a). Histoscores were calculated for each of the patient samples, as the sum of nuclear intensity and the proportion of stained cells (Supplementary Table S1). We identified high p53 histoscores in four of the five patient samples (80%). A single patient (Patient 2) had no detectable p53 overexpression either in the primary or the two metastatic samples. Notably, in the primary PC tumour with no detectable p53, the histoscores of MDM2/4 were high and were also elevated in the metastases, consistent with the capacity of MDM4 and MDM2 to diminish wt p53 protein levels (as we reviewed [15]). Focusing on the 12 metastatic samples specifically (Figure 1b), as particularly relevant to lethal disease, MDM4 histoscores were universally high in all these samples (100%), independent of p53 staining and the wide variety of MDM2 histoscores (ranging from low to high).

To substantiate our preliminary findings regarding MDM levels, we analysed additional primary PC samples (*n* = 120, from Rome), using tissue microarray (TMA) screening (Figure 1c,d). High MDM4 histoscores were also universally measured in these primary PC samples, independent of both the variance in accompanying expression levels of p53 (ranging from low to high), and also generally low MDM2 histoscores. MDM2 histoscores were low or undetectable in eight of the 12 metastatic samples (66%) and modestly elevated in only four samples (Figure 1d). The high levels of MDM4 predict that it is likely to pose a greater oncogenic risk, compared with MDM2, in both a wt p53 context and also following p53 mutation. The prominence of MDM4 in PC samples substantiated our rationale to address the contribution of its high levels to the progression of PC.

To investigate the interplay between *MDM4* expression and *TP53* status in PC, we analysed The Cancer Genome Atlas (TCGA) database. *MDM4* mRNA expression was significantly higher in PC than the normal prostate, and unexpectedly, levels trended highest with mutant *TP53*, above the wt p53 PC counterparts (Figure 1e). To test if *MDM4* expression levels correlated with disease progression, we compared high-grade localized primary PC and metastatic autopsy samples, which had been sequenced by the Tomlins lab and uploaded in Oncomine [37]. The expression levels of *MDM4* mRNA trended higher in samples from metastatic PC samples as compared with those from primary PC (Figure 1f), which is consistent with a separate study [38]. Further interrogation of a data set of 150 metastatic PC samples in CBioPortal [34] revealed *MDM4* DNA amplification only in a minority of samples (<10%). By contrast, in the majority of samples (~60%), high *MDM4* expression levels are likely to be a direct result of its increased mRNA production/longevity (Supplementary Figure S1).

The elevated levels of *MDM4* mRNA in metastatic samples (Figure 1f) raised the possibility of association between *MDM4* expression and PC prognosis. We analysed TCGA primary PC dataset to correlate *MDM4* mRNA expression with patient survival. Our analysis revealed that PC patients with the highest levels of *MDM4* expression in their primary PC tumours showed reduced survival probability as compared with those with lower *MDM4* levels (Figure 1g). By contrast, levels of *MDM2* showed no significant correlation with the survival probability of PC patients (Supplementary Figure S2).

Overall, our analyses are consistent with previous findings of moderate-to-low frequency of *TP53* mutations in primary PC [13]. Relative to normal prostate, we found *MDM4* expression to be elevated in PC, with the highest trend observed in mutant p53 PC samples. *MDM4* levels also trended highest in metastatic PC, relative to the primary site. Our results suggest that elevation of *MDM4* levels is likely to be an early oncogenic event in PC with the potential to contribute to PC progression and to poor outcomes. On the other hand, *MDM2* expression was not consistently altered in PC, nor did its expression correlate with PC prognosis.

### 3.2. MDM4 Expression Is Required for the Growth of PC Cells In Vitro

**Figure 2:**
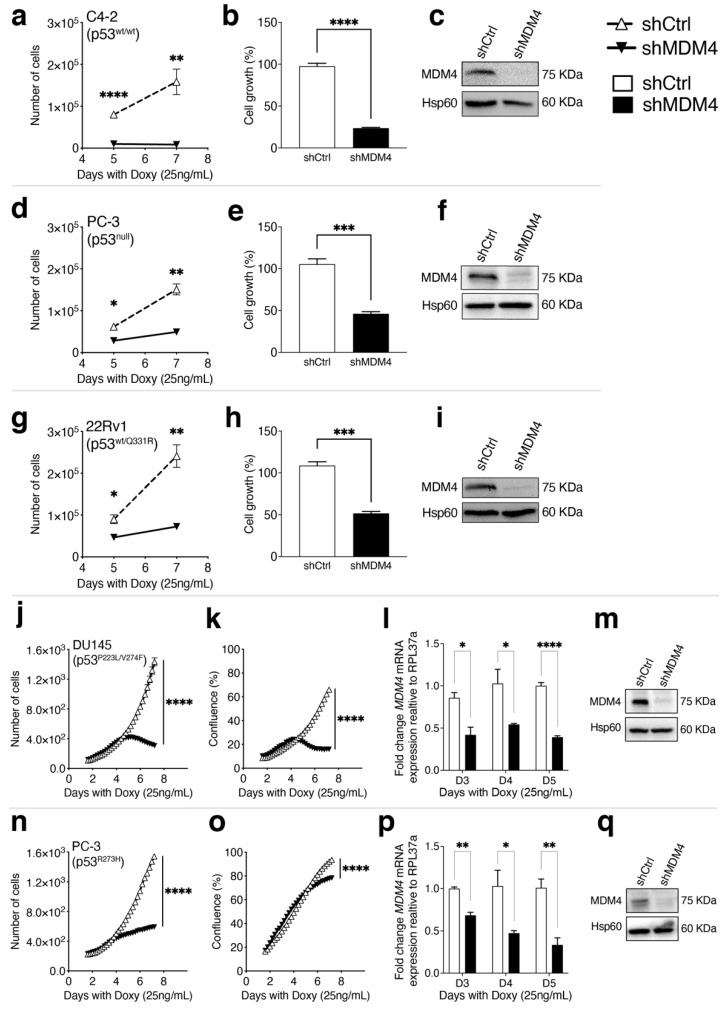
MDM4 knock down impeded the in vitro proliferation of prostate cancer cell lines that are p53 wild-type, null and mutant. Five PC cell lines of distinct p53 status were transduced with inducible shRNA to generate either MDM4 knock down (shMDM4) or counterpart controls (shCtrl). Expression of the shRNAs was induced with Doxycycline (Doxy; 25 ng/mL). (**a**,**d**,**g**) The effects on cell numbers in response to Doxycycline treatment were determined for each PC cell line up to day 7. (**b**,**e**,**h**) Cell growth assessed by SRB assay was calculated as a proportion of the control groups on day 5. Changes in (**j**,**n**) cell numbers and (**k**,**o**) confluence upon shRNA expression were determined for two mutant p53 expressing lines using the Incucyte^®^ system up to day 7. (**c**,**f**,**i**,**m**,**q**) MDM4 protein levels were analysed by Western blot on day 5. (**l**,**p**) *MDM4* mRNA levels were analysed by RT-qPCR for two mutant p53 lines on days 3, 4 and 5. *MDM4* levels were normalised to the housekeeping gene *hRLP37a* and expressed relative to shCtrl. Data are shown as mean ± SEM (*n* = 3–6). Statistical significance was calculated using a two-tailed Student’s *t*-test (* *p* ≤ 0.05, ** *p* ≤ 0.01, *** *p* ≤ 0.001, **** *p* ≤ 0.0001). The Raw Western blot data is shown in Figure S8.

Based on the higher expression of *MDM4* in PC samples, as compared to normal tissue (Figure 1e), we hypothesized that MDM4 is required to drive PC cell growth. It was also important to establish whether its oncogenic influence would be restricted to wt p53-expressing PC cells. To address this, we measured the effects of *MDM4* knockdown (KD) on the growth of PC cell lines that harbour either wt or missense mutant *TP53* or lack *TP53*. This was achieved by transducing PC cell lines with lentiviral-mediated Doxycycline (Doxy)-inducible shRNA against *MDM4* (shMDM4). As a control we used shRNA directed against mouse *Mdm4* sequence (shCtrl; with the most efficient shMDM4 primers selected and control developed as previously described [25]). *MDM4* KD reduced the proliferation of C4-2 PC cells (p53^wt/wt^), as enumerated at day 5 and 7 (Figure 2a), and corroborated by total protein content (day 5, using Sulforhodamine B assay, SRB) [39] (Figure 2b), with coincident reduction in MDM4 protein levels (day 5, Figure 2c). Similar outcomes were measured in two additional PC cell lines: PC-3 (p53^null^, Figure 2d–f), and 22Rv1 (p53^wt/p53Q331R^, Figure 2g–i).

These results suggested that depletion of MDM4 reduced the growth of PC cell lines expressing wt p53, no p53 and even heterozygote wt/mutant p53. The latter is of interest, as most advanced metastatic PC expresses mutant p53 [13]. For a detailed characterisation of the impact of MDM4 depletion on mutant p53-expressing PC, we measured the growth kinetics and cellular responses of DU145 (p53^P223L/V274F^) using the Incucyte^®^ system. DU145 cells were incubated in the Incucyte^®^ Live-Cell Analysis system, with scanning conducted periodically for seven days, to explore the responsiveness to *MDM4* KD (Figure 2j,k; Supplementary Figure S3). We confirmed *MDM4* mRNA KD on day 3, 4 and 5, using reverse transcription-quantitative PCR (RT-qPCR) (Figure 2l). Diminished MDM4 protein was measured on day 5 by Western blot (Figure 2m). *MDM4* KD markedly reduced the proliferation of DU145 cells expressing mutant p53 after day 5 (Figure 2j), corresponding to a reduction in surface area occupancy (Figure 2k).

We were concerned to verify that this inhibitory effect was not exclusive to DU145 cells harbouring these two specific *TP53* missense mutations. In the absence of relevant PC cell lines harbouring *TP53* missense mutations that are prevalent in PC, we generated a derivative of the p53 null cell line PC-3 (p53^null^), engineered to express mutated p53-R273H [PC-3 (p53^R273H^)]. We selected this mutant as it is a frequently altered amino acid residue in advanced PC [40]. shMDM4 inhibited PC-3 (p53^R273H^) cell proliferation and increased surface occupancy (Figure 2n,o; Supplementary Figure S3), with RT-qPCR confirmation of lowered *MDM4* mRNA levels across days 3, 4, 5 (Figure 2p) and Western blot demonstration of reduced MDM4 protein level at day 5 (Figure 2q).

Notably, the inhibited proliferation in response to *MDM4* KD in DU145 (p53^P223L/V274F^) and PC-3 (p53^R273H^) (Figure 2j,n; respectively), was accompanied by distinct and significant morphological changes in each line (Supplementary Figure S3). DU145 cells decreased in size, whereas PC-3 cell size increased, as evident from confluence measures relative to the cell numbers (Figure 2j,k,n,o; respectively). The differences in response to *MDM4* KD in the context of these distinct p53 mutants, suggested that the molecular mechanism(s) of the reaction to MDM4 inhibition is significantly different between these two PC cell lines. Taken together, these results support the notion that *MDM4* depletion hinders the proliferation of all these PC cells; however, the nature of these physiological manifestations differs in individual PC cell lines, each of which holds distinct p53 alterations.

To corroborate the impact of *MDM4* downregulation on PC cell growth using an alternative approach to shRNA, we tested the efficacy of XI-011, a small molecule inhibitor of *MDM4* expression [27]. This was particularly relevant to an additional PC line with an endogenous p53 mutation, VCaP (p53^R248W/null^) (which proved technically challenging to transduce with shRNA, due to toxicity). XI-011 has been reported to inhibit the expression of *MDM4* in uveal melanoma [22], head and neck [41], and breast cancers [25]. We found that XI-011 inhibited cell proliferation in a concentration-dependent manner in the PC lines: C4-2 (wt p53), DU145 (mutant p53) and PC-3 (p53 null), as shown using the SRB assay (Supplementary Figure S4a–c). DU145 exhibited the greatest sensitivity (Supplementary Figure S4d). PCR corroborated *MDM4* expression across these cell lines (Supplementary Figure S4e). VCaP exposed to XI-011 (24 h; 0.05–0.8 μM) was also growth inhibited, as measured using the CellTiter-Blue Cell Viability assay (Supplementary Figure S4f); with reduced MDM4 levels confirmed in response to these treatments (Supplementary Figure S4g). Taken together, these data support a critical role for MDM4 in PC cell proliferation and/or survival, and that its depletion reduces PC cell growth independently of p53 status.

### 3.3. MDM4 Knockdown Causes Apoptotic Death In Vitro in PC Cells with Endogenous Mutant p53

Wt p53-dependent growth inhibition is the best-characterised response to MDM4 targeting (as reviewed [24]). Our results here show that *MDM4* KD also inhibited the growth of PC cells that acquire mutant p53. This finding prompted us to investigate the nature of this retardation in DU145 and PC-3 (p53^R273H^) PC cell lines. As these cells exhibited different rates of growth inhibition (Figure 2) and distinct morphological characteristics (Supplementary Figure S3) in response to *MDM4* KD, we explored the nature of the responses individually.

**Figure 3:**
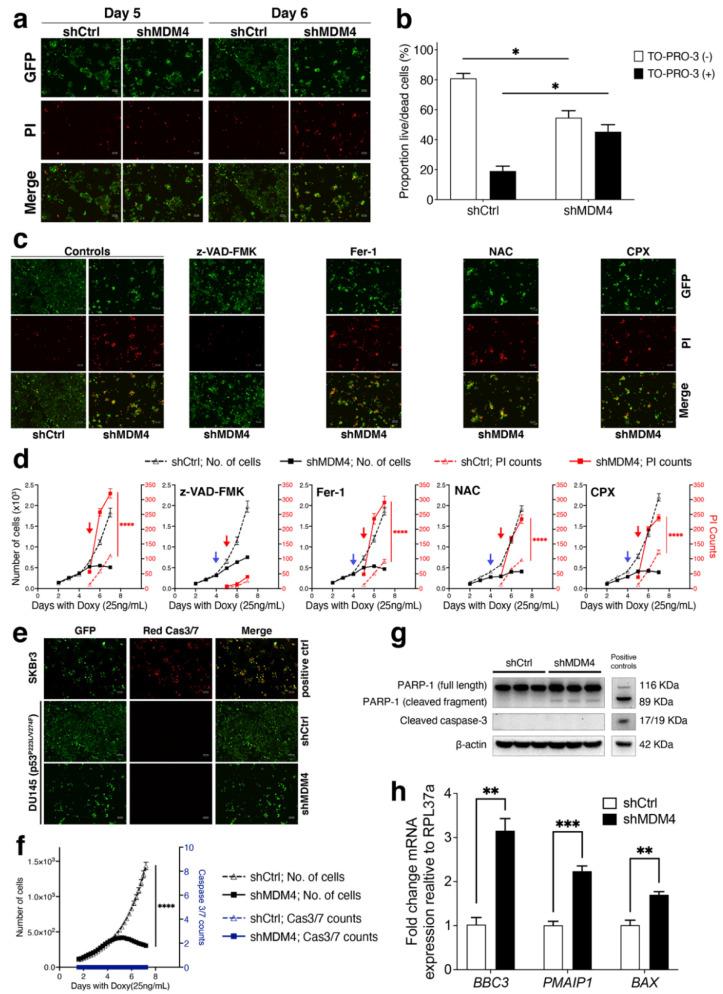
MDM4 inhibition causes apoptosis independently of PARP-1 cleavage and caspase 3/caspase 7 activation in the DU145 prostate cancer cell line in vitro. (**a**) shRNA expression was induced with Doxycyline (Doxy; 25 ng/mL) in mutant p53 and GPF-tagged DU145 cells. Cells were stained with propidium iodide (PI) on day 5 and 6. Representative fluorescence microscopy images of DU145 are shown. (**b**) Flow cytometry analysis of the proportion of live/dead DU145 cells using TO-PRO-3 following *MDM4* KD in response to 5 days of Doxycycline exposure. (**c**,**d**) After the initial Doxycycline treatment (day 1) for shRNA induction, z-VAD-FMK (25 μM), Fer-1 (10 µM), NAC (2.5 mM), and CPX (0.5 µM) were added on day 4 (indicated by the blue arrow). PI was added on day 5 (indicated by the red arrow) and was used to unveil cell death (with PI counts plotted in red). The live-cell imaging Incucyte^®^ system was used to track the effects on the cell growth and cell death kinetics of z-VAD-FMK, Fer-1, NAC, and CPX, following MDM4 inhibition. (**c**) Representative fluorescence microscopy images. (**e**,**f**) The SKBr3 (GFP tagged) cell line was treated with 20 µM of doxorubicin and used as an apoptosis-positive control (see Western blot in Figure S9). shRNA expression was induced with Doxycycline (25 ng/mL) in DU145 for 7 days. On day 2, cells were treated with Red Incucyte^®^ Caspase-3/7 Dye for detecting apoptosis. (**e**) Representative fluorescence microscopy images. (**f**) Cell growth rate and kinetic activation of caspase 3/7 were monitored using the live-cell imaging Incucyte^®^ system. (**g**) Following MDM4 inhibition, protein was extracted on day 5 for exploring the activation of caspase 3 and PARP-1 by Western blot. Each column corresponds to a biological replicate. (**h**) DU145 was treated with Doxycycline and collected on day 5 for RNA extraction. *BBC3* (*PUMA*), *PMAIP1* (*NOXA*), and *BAX* mRNA levels were analysed by RT-qPCR; the graph depict the mRNA fold change levels normalised to the housekeeping gene *hRLP37a* and expressed relative to shCtrl. In representative fluorescence microscopy images, scale bars indicate 100 μm. Data are shown as mean ± SEM (*n* = 3–6). Statistical significance was calculated using a two-tailed Student’s *t*-test (* *p* ≤ 0.05, ** *p* ≤ 0.01, *** *p* ≤ 0.001, **** *p* ≤ 0.0001). The Raw Western blot data is shown in Figure S10.

*MDM4* KD in DU145 cells caused them to shrink, bleb and detach, associated with the loss of adhesion. These observations prompted us to ask whether the cells were dying. Indeed, in response to *MDM4* KD, in conjunction with the significant reduction in DU145 cell numbers (Figure 2j), we observed an increase in the number of cells stained by the nucleic acid intercalating dye Propidium Iodide (PI), which permeates cells upon their death (Figure 3a). PI positivity was evident at day 5 and pronounced at day 6 in response to *MDM4* KD. Quantification of this increased death was undertaken on day 5, using flow cytometry to measure incorporation of nucleic acid stain TO-PRO-3 Iodide (Figure 3b). Cell death, rather than protracted growth inhibition in response to *MDM4* KD was consistent with a lack of cellular senescence features, including the absence of senescence-associated beta-galactosidase (SA-β-gal) staining and relevant morphological changes (i.e., cell enlargement and flattening; Supplementary Figures S3 and S5).

Two main forms of death have been linked to the p53–MDM2/4 pathway: apoptosis and ferroptosis (as reviewed in [42]). Notably, ferroptosis is a form of regulated cell death induced by excessive lipid peroxidation and recently linked to high MDM4 levels (independent of p53), as demonstrated in short-term experiments (< 4 days) in glioblastoma (GBM) cells, by the Stockwell-Prives team [43]. To elucidate the form of death instigated by *MDM4* KD in mutant p53 PCs, we attempted a series of rescue experiments. As caspases are key enzymes involved in apoptosis, but not required for ferroptosis, we adopted a pan-caspase inhibitor (z-VAD-FMK). Separately, we introduced specific ferroptosis pathway inhibitors: a lipophilic antioxidant, Ferrostatin-1 (Fer-1); an antioxidant, N-acetylcysteine (NAC); and an iron chelator, Ciclopirox (CPX). We compared the rescue capacity of z-VAD-FMK to ferroptosis inhibitors (Figure 3c,d). Only z-VAD-FMK efficiently inhibited cell death induced by *MDM4* KD, in DU145 cells, as measured by PI staining. The failure of Fer-1, NAC and CPX to rescue the *MDM4* KD-mediated cell death in DU145 cells, indicated that ferroptosis was not their main death pathway.

To provide further detail regarding the role of caspases in the death process, we tested for activation of caspase 3 and 7 using a Red Incucyte^®^ caspase 3/7 dye over a time course (Figure 3e,f). There was no evidence of their activation using this approach and these findings were reiterated by Western blot, where cleavage of caspase 3 was not detected in DU145 cells in response to *MDM4* KD on day 5. Consistently in these cells, cleavage of PARP-1, which is the classic target of caspase 3/7, was also not detected (Figure 3g). This dependency on caspases is relevant to apoptosis, although our data indicates that the effect is not driven along the canonical caspase 3/7 pathway. Elevated expression of key apoptosis mediators *BBC3* (*PUMA*), *PMAIP1* (*NOXA*) and *BAX* further support the induction of apoptotic cell death in DU145 cells in response to *MDM4* KD (Figure 3h). These additional apoptotic characteristics contradict the involvement of necrotic cell death pathways that have recently been shown to involve caspases (e.g., [44]). It is relevant to add that in VCaP, treatment with XI-011 triggered MDM4 depletion (Supplementary Figure S4g) and induced BAX elevation and PARP cleavage and was also accompanied by p73 elevation most noticeable at 72 h; together with a decrease in MDM2 levels and a slight increase in p53 (Supplementary Figure S4g). These data are also indicative of an apoptotic type of death, although involving distinct responses compared to DU145 cells.

### 3.4. Downregulation of MDM4 Attenuates the Tumour Growth of DU145 Xenotransplants In Vivo

**Figure 4:**
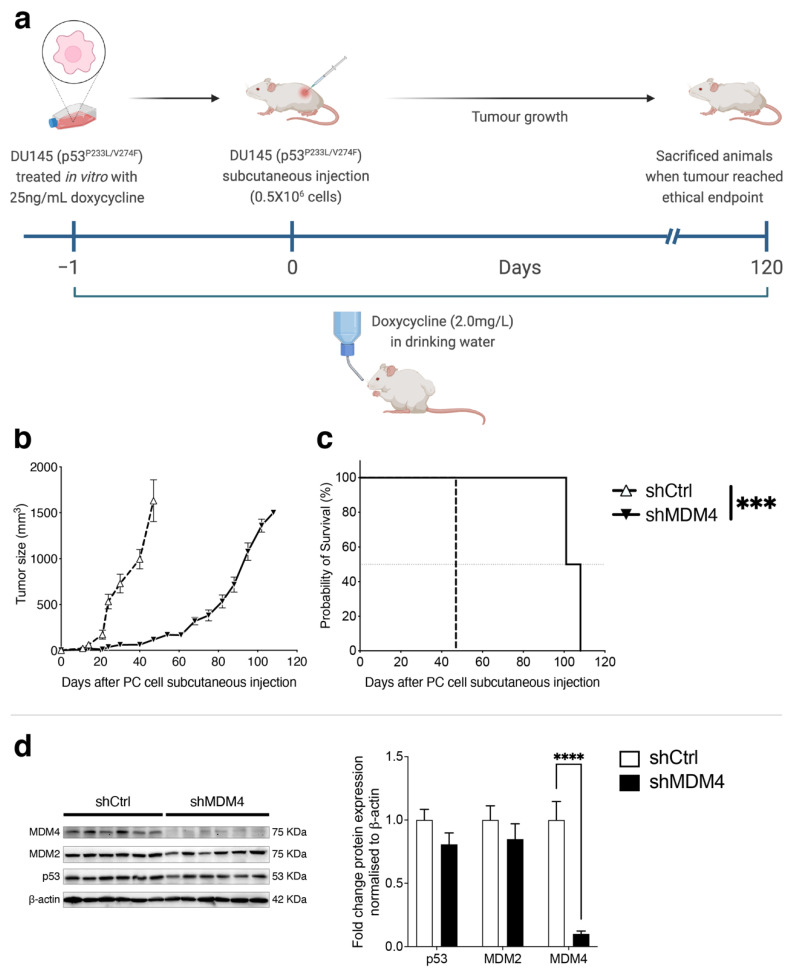
MDM4 expression is necessary for the in vivo growth of prostate cancer cells with mutant p53. DU145 mutant p53 PC cell line transduced with either Doxycycline-inducible shMDM4 or shCtrl was subcutaneously injected into the contra-lateral flanks of 6–8 weeks old male NOD SCID gamma IL2R-gamma chain (NSG) mice (*n* = 6 per treatment cohort). (**a**) DU145 cells were pre-treated in vitro with Doxycycline (25 ng/mL) for 24 h before injection into the mice. Doxycycline was supplemented in the drinking water (2.0 mg/L) until ethical endpoint and subsequent sacrifice. (**b**) Tumour volume and (**c**) survival percentage were measured. Statistical significance shown as result of Log-rank (Mantel–Cox) test. (*** *p* ≤ 0.001, **** *p* ≤ 0.0001). When the ethical endpoint was reached (∼1500 mm^3^), tumours were collected, and protein was extracted. (**d**) p53, MDM2 and MDM4 protein levels were analysed by Western blot. Each column in the Western blot corresponds to a biological replicate. The graph shows the Western blot’s densitometric analysis; protein levels were normalised to β-actin and expressed relative to shCtrl. Data are shown as mean ± SEM of biological replicates. Statistical significance was calculated using a two-tailed Student’s *t*-test (*** *p* ≤ 0.001, **** *p* ≤ 0.0001). The Raw Western blot data is shown in Figure S11.

Having established a form of apoptotic death in DU145 cells in response to *MDM4* KD in vitro, we questioned whether this effect could be harnessed in vivo. This is particularly relevant to the almost universal link between *TP53* missense mutations and lethal metastatic PC [12]. As patients at risk standardly undergo resection of their primary disease, it is the dispersed tumour growth that typically kills the patient. To test the efficacy of *MDM4* KD prophylaxis in a PC mouse model, we xenotransplanted DU145 cells into the contralateral flanks of immunocompromised NOD/SCID/IL2rγ^null^ (NSG) male mice (Figure 4a). Mice either received cells transduced for shMDM4 or shCtrl (Figure 4a). Mice injected with shCtrl DU145 cells established tumours rapidly and reached the ethical endpoint (1500 mm^3^) at ~47 days (Figure 4b). At this time point, shMDM4 injected mice had tumour volumes of ~115 mm^3^, and only reached the endpoint (1500 mm^3^) at ~108 days. It is relevant to observe that tumour growth in these mice was strongly inhibited up to around 60 days, from which point proliferative growth became overwhelming. All mice were culled at the ethical endpoint. The survival analysis revealed that *MDM4* KD extended mice survival by >2-fold compared to the shCtrl (Figure 4c). Our studies demonstrated that downregulation of *MDM4* expression as a single treatment attenuated the growth of PC with mutant p53 in vivo, consistent with the in vitro findings. The ultimate capacity of the tumours to overcome the inhibitory effects of *MDM4* KD, however, predicts that additional events were selected in vivo to circumvent the inhibitory impact of *MDM4* KD (where *MDM4* KD is maintained at end point in these tumours Figure 4d). This highlights the relevance of understanding the cell inhibitory mechanisms that are invoked by *MDM4* KD as a basis for formulating an augmented therapeutic approach, as subsequently explored.

### 3.5. A Cellular Senescence Response Is Induced in PC-3 (p53^R273H^) Following MDM4 KD

**Figure 5:**
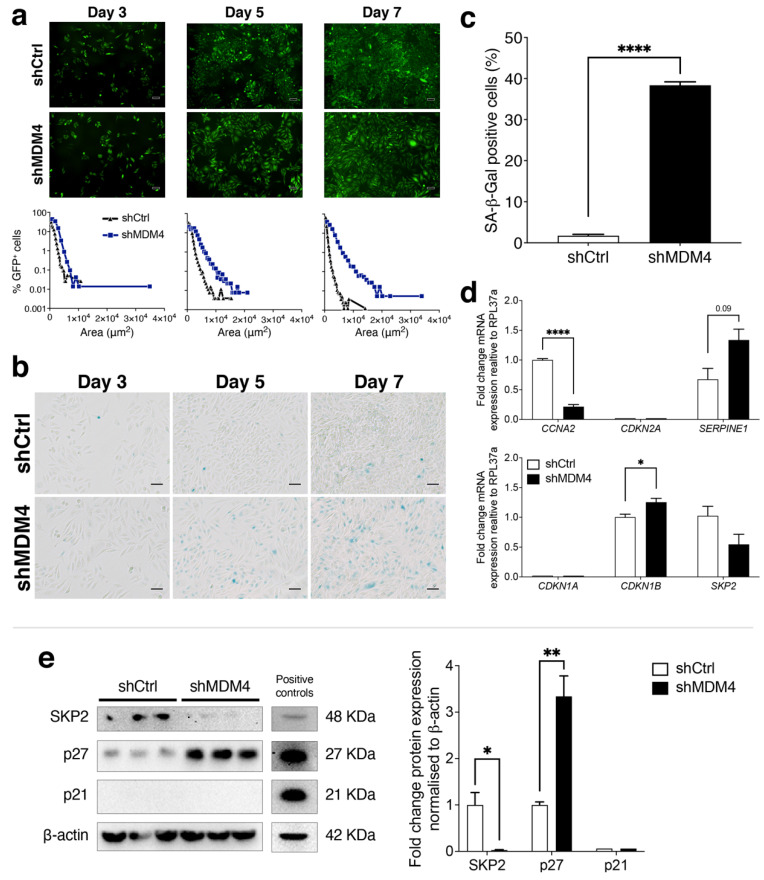
Following MDM4 inhibition, PC-3 (p53^R273H^) prostate cancer cell line undergoes cellular senescence in vitro, in a process strongly associated with the SKP2/p27 pathway. Either shMDM4 or shCtrl expression was induced with Doxycycline (Doxy; 25 ng/mL) in GPF-tagged (GFP^+^) PC-3 (p53^R273H^). (**a**) High-resolution fluorescence photos were taken using the Incucyte^®^ system on days 3, 5, and 7 to explore cell morphology changes upon *MDM4* KD. Images were analysed using the Incucyte^®^ Live-Cell Analysis System and the Cell-by-Cell Analysis Software Module. Histograms comparing the distribution frequency of the fluorescent area (μm^2^) of all cells plated at a given time point (either day 3, 5 or 7). The software automatically determined the histogram bins. Data are shown as a percentage of GFP^+^ cells. (**b**) Senescence-associated β-galactosidase (SA-β-gal) staining at pH 6 of the PC-3 (p53^R273H^) cell line on day 3, day 5 and day 7 after induction of either shMDM4 or shCtrl expression with Doxycycline. SA-β-gal-positive cells stained blue. Scale bars indicate 100 μm. (**c**) Graph shows the percentage of senescent cells relative to the total number of cells (per image) on day 7 of three biological replicates. (**d**) PC-3 (p53^R273H^) cells were collected after 5-day treatment with Doxycycline for RNA and protein analyses, respectively. Senescence-related genes *CCNA2* (Cyclin A2), *CDKN2A* (p16), *SERPINE1* (PAI-1), *CDKN1A* (p21), *CDKN1B* (p27), and *SKP2* (SKP2) mRNA levels were analysed by RT-qPCR. mRNA expression levels were normalised to the housekeeping gene *hRLP37a* and expressed relative to shCtrl. (**e**) Protein levels of p21, p27 and SKP2 were determined by Western blot. Each column corresponds to a biological replicate. The graph shows the Western blot densitometric analysis of p21, p27 and SKP2 protein levels normalised to β-actin and expressed relative to shCtrl. In all cases, data are shown as mean ± SEM of biological replicates (*n* = 3–6). Statistical significance was calculated using a two-tailed Student’s *t*-test (* *p* ≤ 0.05, ** *p* ≤ 0.01, **** *p* ≤ 0.0001). The Raw Western blot data is shown in Figure S12.

Following *MDM4* KD, both PC-3 (p53^R273H^) and DU145 cells were growth retarded (Figure 2) but behaved in a manner that was visually distinct from each other. PC-3 (p53^R273H^) cells became substantially enlarged and flattened after *MDM4* KD (Supplementary Figure S3) and occupied more surface area (Figure 2o). Importantly, a key phenotype of senescence cells in vitro is the adoption of enlarged, flat and irregular cell shape. Since targeting MDM4 has been linked in some cancer types to cellular senescence (e.g., [19,25,45], it was pertinent to measure the effect of *MDM4* KD on cell morphology and SA-β-gal staining of PC-3 (p53^R273H^) cells. To quantify the morphological changes in PC-3 (p53^R273H^) attributable to *MDM4* KD, we adopted the Incucyte^®^ system and analysed high-resolution fluorescence images taken on day 3, 5, and 7 after shRNA induction (Figure 5a). *MDM4* KD caused a temporal expansion in cell size (measured as area gain). These morphological changes were accompanied by SA-β-gal staining positivity, indicative of senescence (Figure 5b), which was evident at day 5 and pronounced at day 7 in ~40% of cells (as quantitated in Figure 5c).

To elucidate the molecular mechanisms involved in this senescence response in PC-3 (p53^R273H^) cells, we measured the impact of the *MDM4* KD on the expression of key drivers of cellular senescence and relevant genes that have been linked to MDM4. We measured induction of the senescence-promoting gene *SERPINE1* (PAI-1) [46] and a reduction in *CCNA2* (CyclinA2), which is associated with cellular senescence [47]. *CDKN2A* (p16) was not detected in this cell line (Figure 5d). A key regulator of the cell cycle and cellular senescence is p27, which is regulated by its E3 ligase, Skp2 [48]. We identified that *MDM4* KD markedly increased *CDKN1B* (p27) mRNA (Figure 5d) and its protein product p27 (Figure 5e). We also observed the corollary, with a trending decrease in *SKP2* mRNA (Figure 5d) and a drop in its protein expression (Figure 5e). The cell cycle inhibitor p21 encoded by *CDKN1A*, that is a key target of wt p53, did not reach levels of detection either for mRNA (Figure 5d) or protein (Figure 5e). Together these results support the ability of *MDM4* KD to promote cellular senescence in PC-3 (p53^R273H^) cells through multiple downstream targets including the SKP2-p27 axis. Consistently, in response to *MDM4* KD there was no evidence of caspase 3/7 activation (Supplementary Figure S6a,b), or PARP-1 cleavage (Supplementary Figure S6c, The Raw Western blot data is shown in Figure S15). Apoptotic marker genes *BAX* and *PMAIP1* were also not elevated (Supplementary Figure S6d), in contrast to their altered levels in DU145 (Figure 3h). Our results support the onset of cellular senescence as a dominant growth inhibitory process induced in PC-3 (p53^R273H^) in response to *MDM4* KD. Overall, our findings from DU145 and PC-3 (p53^R273H^) uncovered that *MDM4* KD inhibits proliferation in PC cells expressing mutant p53, in a cell type- and context-dependent manner.

### 3.6. Treatment of PC Cells by Targeting MDM4 Alone or in Combination with APR-246

The ability of *MDM4* KD to inhibit DU145 cell growth in vivo indicated its therapeutic potential (Figure 4). However, the ultimate outgrowth of the tumour cells exposed that this single agent approach is insufficient. This implicated a rationale for complementary treatment administration. As we have demonstrated that ferroptosis is not the major cell inhibitory response induced by *MDM4* KD in either DU145 or PC-3 (p53^R273H^) cells, we speculated that inducing this mechanism in parallel could pose a potent approach to targeting PC cancers with mutant p53.

**Figure 6:**
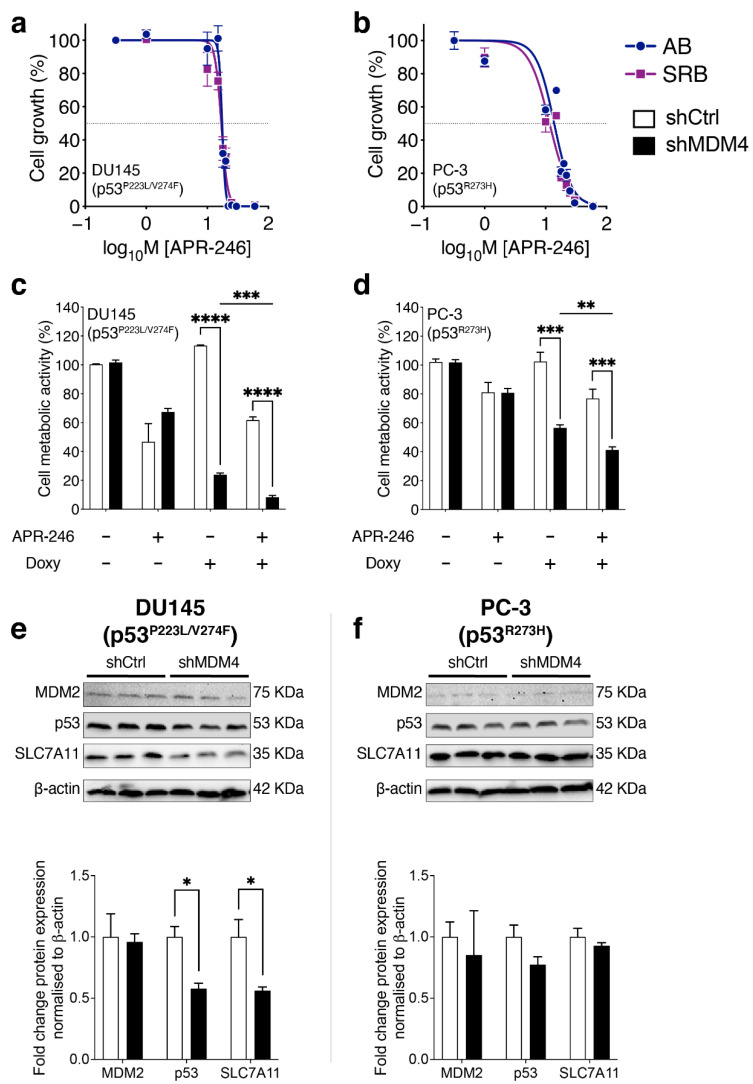
Combination therapy: MDM4 inhibition and eprenetapopt (APR-246) attenuate the cell growth of mutant p53 expressing PC cell lines in vitro. (**a**,**b**) PC cell lines DU145 (p53^p223L/V274F^) and PC-3 (p53^R273H^) were treated in vitro with APR-246 (eprenetapopt) at concentrations ranging from 0 µM to 60 µM for a period of five days prior to assessment. APR-246 dose–response was evaluated by assessing the suppression of cell growth using Alamar blue (AB) and sulforhodamine B (SRB) assays. (**c**,**d**) To examine whether APR-246 increases the efficacy of MDM4 inhibition, PC cell lines were treated either with IC_30_ of APR-246 alone or in combination with Doxycycline (Doxy 25 ng/mL) over a period of 5 days. Treatment response was evaluated by assessing the suppression of cell growth using Alamar blue assay. (**e**,**f**) shRNA expression was induced with Doxycycline (25 ng/mL) in mutant p53 PC cells. Cells were collected on day 5 for protein expression analyses. Protein levels of MDM2, p53, and SLC7A11 were determined by Western blot. Each column corresponds to a biological replicate. The graphs show the Western blot densitometric analysis of MDM2, p53, and SLC7A11 protein levels normalised to β-actin and expressed relative to shCtrl. Data are shown as the mean ± SEM of biological replicates (*n* = 3–6). Statistical significance was calculated using a two-tailed Student’s *t*-test (* *p* ≤ 0.05, ** *p* ≤ 0.01, *** *p* ≤ 0.001, **** *p* ≤ 0.0001). The Raw Western blot data is shown in Figure S13.

Recent work identified that the small molecule pro-drug eprenetapopt (used interchangeably in our figures as APR-246) promotes ferroptosis in a range of cancer cells [49,50]. Of relevance, ferroptosis is suppressed in the presence of high levels the cystine-glutamate antiporter SLC7A11, which imports cystine across the plasma membrane, feeding the biosynthesis of the antioxidant glutathione. Work from the Clemons lab in collaboration demonstrated that eprenetapopt activity correlates inversely with levels of the SLC7A11 in a variety of cancer cell types, including those expressing mutant p53 [51,52]. Importantly, safe and efficacious application of eprenetapopt was demonstrated in early pharmacokinetic studies in PC patients [53]. We first measured responses to eprenetapopt as a single agent, across a range of concentrations in DU145 and PC-3 (p53^R273H^) cells (Figure 6a,b, respectively), using colorimetric assays (Alamar blue and SRB). Moreover, eprenetapopt concentrations that inhibited the cell growth of three additional PC cell lines were determined (IC_50_ at day 5; Supplementary Table S2). As PC-3 (p53^R273H^) is a derivative of PC-3 (p53^null^), it was relevant to compare the relative impact of eprenetapopt between them. Notably, PC-3 (p53^R273H^) (as shown for two independent clones) was nearly twice as sensitive to the drug as PC-3 (p53^null^) [IC_50_ of the two mutant clones ~16 mM and 32.5 mM for the p53 null cell line], as measured by relative metabolic activity using Alamar Blue. These PC-3 (p53^R273H^) lines, isogenic for mutant p53, demonstrate that the presence of mutant p53 is physiologically relevant to eprenetapopt treatment efficacy. It is also notable that DU145 and PC-3 (p53^R273H^) demonstrated similar sensitivities to eprenetapopt.

On the basis of the single-agent efficacy of both *MDM4* KD and eprenetapopt respectively, we tested their combined impact on cell proliferation in vitro. The IC_30_ of eprenetapopt for each cell line, as relevant to day five, was selected for co-treatment with *MDM4* KD. The effect of *MDM4* KD and eprenetapopt was additive, with cell metabolic activity markedly reduced in DU145 cells at day five (Figure 6c). It is also highly relevant that this co-treatment had a significant impact on PC-3 (p53^R273H^) using its eprenetapopt IC_30_ (Figure 6d), where in contrast, at this concentration it did not impact PC-3 (p53^null^) cells, either alone or in combination with *MDM4* KD (Supplementary Figure S7b).

As this combinatorial approach appeared to have increased potency beyond either treatment individually in these mutant p53 PC cells, we explored whether MDM4 influences any molecular targets linked to eprenetapopt treatment. As ferroptosis inhibitors were not able to rescue *MDM4* KD growth inhibition in DU145, we looked for other targets of relevance. Notably, it was recently demonstrated by the Clemons lab, in collaboration, that the major predictor for eprenetapopt treatment response was the expression level of SLC7A11 rather than the mutational status of *TP53* [51,52]. This provided the rationale to measure the protein expression levels of SLC7A11 in response to *MDM4* KD in these PC cell lines. Somewhat unexpectedly, *MDM4* KD in DU145 cells, led to a significant decrease in SLC7A11 protein levels in conjunction with a drop in mutant p53 levels (Figure 6e; respectively). In contrast, in PC-3 (p53^R273H^), the levels of these proteins were not significantly changed (Figure 6f). The findings for DU145 are particularly interesting, because they suggest that MDM4 maintains both mutant p53 and SLC7A11 levels, which is of likely benefit to the cancer cells in sustaining redox balance. It also suggests that lowering SLC7A11 is insufficient trigger ferroptosis in these cells to. Importantly though, when levels of SLC7A11 were reduced in response to *MDM4* KD in DU145 cells, this appeared to predispose to eprenetapopt induction of cell growth inhibition. A relevant avenue to explore, which is beyond the scope of this current work, is whether co-treated cells are dying predominantly from ferroptosis or apoptosis.

**Figure 7:**
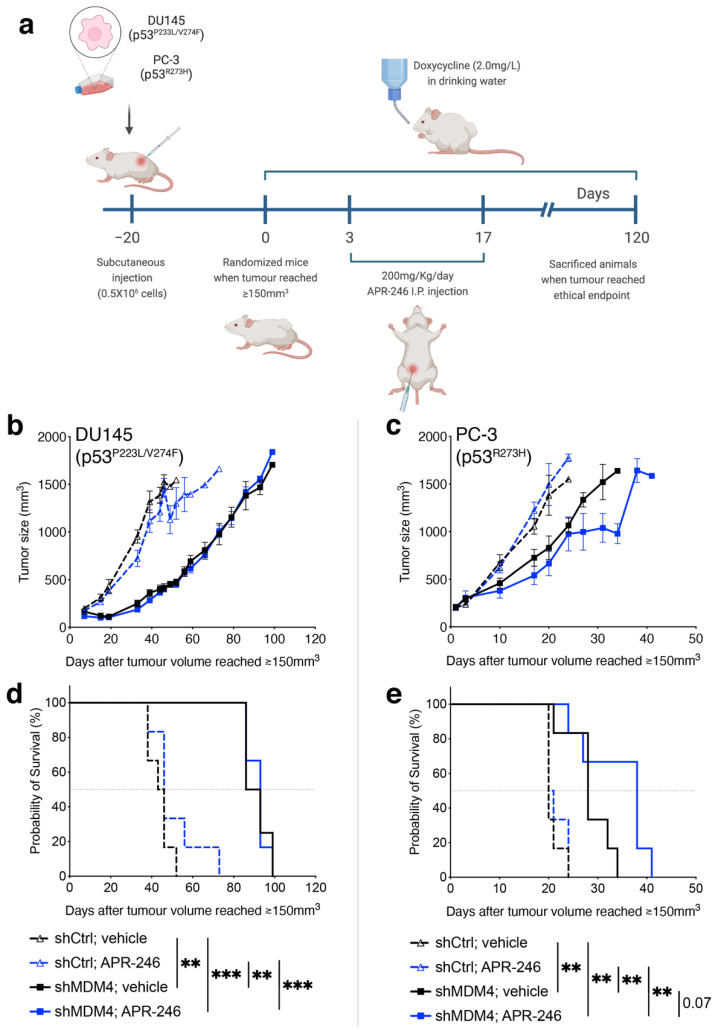
Testing the therapeutic potential of *MDM4* KD in combination with eprenetapopt (APR-246) in PC xenografts that express mutant p53 in vivo. (**a**) NSG male mice between 6–8 weeks were injected subcutaneously with either DU145 (p53^p223L/V274F^) or PC-3 (p53^R273H^) PC cells into their contra-lateral flanks. When tumour growth reached ≥ 150 mm^3^ (day 0), *MDM4* KD was induced by Doxycyline supplementation in the drinking water (2.0 mg/L) until the ethical endpoint was reached (∼1500 mm^3^). APR-246 was given to mice as a 200 mg/Kg intraperitoneal injection (100 mg/Kg twice a day) for 14 days. (**b**) Tumour volume and (**c**) survival percentage were measured. *n* = 6, per treatment cohort. (**d**,**e**) Statistical significance shown as result of Log-rank (Mantel–Cox) test. (** *p* ≤ 0.01, *** *p* ≤ 0.001).

To extend our findings into a more clinically relevant tumour setting, we tested how *MDM4* KD in combination with eprenetapopt affected the growth of DU145 and PC-3 (p53^R273H^)-established xenografts in NSG male mice (Figure 7a). Transplanted tumours were allowed to grow to 150 mm^3^ prior to inducing *MDM4* KD with Doxycycline; this contrasted the prophylactic approach in Figure 4. In this in vivo treatment setting, the sustained KD of *MDM4* as a single treatment slowed tumour growth and significantly improved survival for mouse recipients of both xenotransplanted lines (Figure 7b,c). This is an important finding as it demonstrates the relative efficacy of this treatment approach in models that advance at different paces when left untreated (i.e., DU145 control that reached end point in ~50 days and also the more rapid PC-3 control (p53^R273H^), whose end point was reached at ~25 days). These findings complement the proof of principle of our prophylactic study by demonstrating also the treatment potency of *MDM4* KD in DU145-established xenografts (Figure 4).

At the selected dose of eprenetapopt, a single agent approach did not lead to any significant tumour growth inhibition, although there was a trending survival improvement in mice with DU145 xenotransplants. These observations emphasise that *MDM4* KD yielded much stronger tumour-growth inhibition than eprenetapopt alone in both PC cell lines at the concentration administered. In contrast to the in vitro findings, *MDM4* KD was not potentiated by the addition of eprenetapopt in DU145 xenotransplants (Figure 7b,d). Rather, it was the PC-3 (p53^R273H^) tumours that were growth inhibited by this co-treatment (Figure 7c,e). It is crucial to note that our ethics approved only 14 days of eprenetapopt administration to these mice. It is fascinating to speculate that the tumour cell growth kinetics dictated the relative responses, with the more rapidly growing tumours of PC-3 (p53^R273H^) being more obviously affected by the co-treatment, but then also outgrowing the effect quite rapidly. Moreover, cancer patient’s treatment plans usually include a number of chemotherapy cycles in which they typically undergo 4 to 8 cycles of treatment. It would also be interesting to assess whether multiple eprenetapopt cycles in combination with MDM4 inhibition could yield a better in vivo response. Further exploration of this combined treatment strategy is beyond the scope of our current research and animal ethics approval.

## 4. Discussion

In normal cells, members of the MDM-family, MDM2 and MDM4, keep the growth inhibitory functions of wt p53 in check [54]. Loss of wt p53 function is observed in PC, but the mechanisms beyond its direct mutation are not well understood. This is particularly relevant to localized PC, where wt p53 predominates. Our observations of high expression levels of *MDM4* mRNA in wt p53 PC datasets, relative to normal prostate; and high levels of MDM4 protein in primary PC TMAs, strongly suggest that elevated *MDM4* mRNA and protein expression is common in primary and localised PC. In contrast, MDM2 levels do not show a consistent trend in these contexts. (Figure 1). Evidence of MDM4 growth dependency in wt p53 PC was derived from the C4-2 cell line (Figure 2a–c) and is consistent with an independent study in LNCaP [45]. High MDM4 levels are likely to be selected at early disease stage in PC to keep wt p53 tumour-suppressive activities in check, particularly in response to intra- and extra-cellular stress signals, such as oncogenic events (i.e., loss of *PTEN*) and inflammation (e.g., [55]). Our findings predict that oncogenic MDM4 inhibits wt p53 functions during PC pathogenesis, as consistent with previous reports in melanoma [23] and breast cancers [19], where oncogenic activity of MDM4 has also been identified to suppress wt p53 functions [56].

Importantly, we also found that MDM4 levels are high in PC metastases (Figure 1) (which commonly associate with *TP53* mutations, e.g., [12]); this suggests that oncogenic activities of MDM4 independent of wt p53 are being selected. Further, these findings invited the suggestion that oncogenic MDM4 may also augment mutant p53 function (e.g., [16,57,58]). This aligns with our findings of dependency on MDM4 for cell proliferation in PC lines: p53 null PC-3 (Figure 2d–f), and also in mutant p53 DU145 (Figure 2j–m), VCaP (Supplementary Figure S4f,g) and PC-3 (p53^R273H^) (Figure 2n–q). This concept was supported by our in vivo xenotransplant models, where delayed PC progression resulted from MDM4 ablation (Figure 4 and Figure 7). Our novel findings in PC are consistent with dependency on high levels of MDM4 in triple-negative breast cancers (TNBCs) expressing mutant p53 (e.g., [25,59] and further with accelerated tumourigenesis in mouse mammary gland tumours with mutant p53 model that overexpressed MDM4 [60]).

Mechanistically, the oncogenic functions of MDM4 that are independent of wt p53 have been attributed to its capacity to suppress p21 and p27 levels [22,35], and also pRb [61]. To uncover the novel MDM4 oncogenic dependencies in the context of mutant p53 PC, we focused on two cell lines expressing distinct p53 mutations of clinical relevance. Although both lines responded to *MDM4* KD, the type of response was distinct. DU145 cells underwent cell death in response to *MDM4* KD. More explicitly, in DU145, *MDM4* KD induced caspase-mediated death, but not through the canonical caspase 3/7 pathway or involving PARP-1 cleavage (Figure 3f,g). Cell death was accompanied by the elevation of a number of pro-apoptotic genes such as *BBC3*, *PMAIP1* and *BAX* (Figure 3h). VCaP, another mutant p53-expressing PC cell line, also demonstrated the characteristic apoptotic feature of *BAX* elevation in response to MDM4 inhibition, using a small molecule compound (XI-011), but differed in other details, such as a robust PARP-1 cleavage (Supplementary Figure S4g). A completely different response was observed in PC-3 (p53^R273H^), which exhibited features of cellular senescence associated with elevated *SERPINE1* and reduced *CCNA2* expression (Figure 5d). Given the previous link of MDM4 with p27 [25] and the role of p27 in cellular senescence [48], it was pertinent to study p27 in PC. We identified increased p27 expression in response to *MDM4* KD in PC-3 (p53^R273H^). This correlated with a reduction in the expression of its E3 ligase, SKP2, strongly implicating the SKP2–p27 axis in this response (Figure 5d,e). It is unclear how *MDM4* KD promotes the reduction of *SKP2* expression, as its regulation is not well understood, although it has been associated with PC progression [62].

Beyond these growth inhibition pathways, our findings suggest that at least in DU145, MDM4 sustains elevated SLC7A11 levels (Figure 6e), which acts against redox stress and resists ferroptosis (e.g., [63,64]). Pertinently, MDM4 also maintains the expression levels of CXCR4, a metastasis-promoting factor [59]. MDM4 can also contribute to MDM2-driven epithelial to mesenchymal transformation [65] and also inflammation ([16] and references therein). Other p53-independent functions of MDM4 are likely to add to PC reliance on its expression, including DNA replication and genomic instability (e.g., [57,58]), cellular metabolism and in particular lipid metabolism [43].

The consistent inhibitory response of multiple PC lines to *MDM4* KD suggests an acquired dependency on MDM4 expression for survival and/or proliferation that defines an “Achilles’ Heel” with exciting therapeutic opportunities. Therapeutic targeting of MDM4 has been identified to be efficient and safer than MDM2 inhibition therapy [21]. Importantly, MDM2 inhibitors, like RG112, demonstrated increased toxicity due to on-target effects in normal tissues, which halted clinical trials [20]. This prompted the search for compounds that target MDM4 or dual inhibitors of both MDM2 and MDM4 (as reviewed [16,20]). The therapeutic potential of targeting MDM4 by decreasing its mRNA expression using XI-011 has been demonstrated [27] across different cancer types, including uveal melanoma [22] and breast cancer [25] cells. Our novel results demonstrate that decreased MDM4 mRNA levels accompanied growth inhibition of PC cells treated with XI-011 (Supplementary Figure S4, The Raw Western blot data is shown in Figure S14).

As single agents are rarely effective, we tested the impact of MDM4 targeting in combination. We chose eprenetapopt (APR-246), which elevate levels of cellular reactive oxygen species through complexation of intracellular free glutathione, and also by inhibiting the antioxidant enzyme thioredoxin reductase (TrxR1), leading to cell cycle arrest or death [66]. We found that the combination of MDM4 targeting and eprenetapopt led to an additive growth inhibition response in vitro (Figure 6c,d). Unexpectedly, we made the fascinating finding that MDM4 depletion led to reduced SLC7A11 levels in DU145 PC cells. This predicts that MDM4 supports elevated SLC7A11 levels in this endogenous mutant p53 context. High SLC7A11 levels prime cellular cystine influx, maintaining glutathione levels and preventing ferroptotic cell death, which in turn poses a risk for resistance to cancer therapies, including in PC (as reviewed [67]). A rational explanation of the potency of combined MDM4 targeting and eprenetapopt in DU145 cells is that depleted MDM4 levels lowered SLC7A11 and in turn decreased intracellular glutathione levels, potentiating the activity of the pro-drug eprenetapopt to complex free glutathione [66], ultimately driving cell death.

The impact of MDM4 inhibition is relevant to a wt p53 context, as activated p53 is known to suppress SLC7A11 levels accompanying ferroptosis induction. We have also previously made the curious observation that mutant p53 is able to suppress SLC7A11 levels when overexpressed [63]. Building on our studies, it is interesting to hypothesize that MDM4 is a critical pivot holding the balance between SLC7A11 levels and either wt or mutant p53. In an overexpression context, such as PC-3 (p53^R273H^), the influence of MDM4 on SLC7A11 may be outweighed by mutant p53. In this case the growth inhibitory influence of *MDM4* KD is likely to involve other oncogenic pathways. Testing this is beyond the scope of this study but offers the potential for future studies relevant to the emerging interest in MDM4 inhibition therapies.

It is also pertinent to discuss our findings pertaining to the recent studies from the Stockwell–Prives groups on GBM, which reported that MDM4–MDM2 promotes ferroptosis through changes to lipid metabolism. This was measured both in a wt p53 and p53 null context, but not in the context of mutant p53 [43]. It is noted that shMdm4 treatment alone, over the entire 48 h period monitored in this study, did not significantly reduce GBM cell viability. This is in keeping with our data, where viability was not affected until at least 4 days post *MDM4* KD induction (Figure 2). Our studies are consistent with an oncogenic role for MDM4 in PC, which is supported by the selection of elevated MDM4 expression in metastatic PC.

Our observations that the potentiation of MDM4 KD by eprenetapopt was mutant p53 dependent is novel and of exciting therapeutic relevance, as demonstrated in the PC-3 isogenic lines (Supplementary Figure S7; Supplementary Table S2). Understanding the inhibitory mechanisms of this co-treatment is central to progressing this as a suitable clinical approach. This study reveals vital novel directions to explore in the quest for new therapeutic strategies for treating PC. It is relevant to add that MDM4 inhibition as a therapeutic approach is gaining attention, with efforts to date largely advanced in a wt p53 context, although not exclusively. There are three major approaches in development: (1) inhibiting MDM4 expression, with small molecule drugs and anti-sense targeting of spliceosome factors; (2) inhibiting p53–MDM4 interaction, predominantly using stapled-peptides; and (3) promoting MDM4 degradation, shown, for example, using Hsp90 inhibitor (17AAG) (as reviewed [24,68]). As relevant to our findings, blocking cystine uptake, most directly through SLC7A11 targeting, is being actively advanced for potentiating hematopoietic therapies [69]. It is also notable that blocking the multi-drug resistance pump 1 (MDR1, also known as P-glycoprotein or P-gp) is being explored for promoting eprenetapopt efficacy, though intracellular entrapment of glutathione-complexed drug to rationally target mutant p53 [70].

## 5. Conclusions

In summary, we identified MDM4 oncogenic functions in both the presence of wt p53 and its absence in PC. Pertinent to the most aggressive PCs, we discovered that MDM4 also promoted pathogenesis when p53 was mutated. Our in vitro and in vivo studies validated MDM4 as a rational therapeutic target across the breadth of PCs tested.

We demonstrated that MDM4 inhibition in PC cells with mutant p53 provoked either senescence or apoptotic-like responses. Importantly, we demonstrated potentiation of MDM4 inhibition with the addition of a redox-altering drug that was selected to target mutant p53. Our studies provide a strong rationale for exploring novel treatments that complement MDM4 targeting in PCs, beyond a strictly wt p53 context, in mutant p53-expressing advanced PC. Our findings predict that MDM4 targeted therapy may be effective in metastatic castrate-resistant prostate cancer driven by mutant p53/MDM4 overexpression that fails conventional therapy.

## Data Availability

The data presented in this study are available in this article and Supplementary Material.

## Supplementary Materials

The following supporting information can be downloaded at: https://www.mdpi.com/article/10.3390/cancers14163947/s1, Figure S1: *MDM4* alteration frequency in metastatic prostate cancer; Figure S2: *MDM2* levels and their impact on prostate cancer patient survival; Figure S3: *MDM4* knockdown inhibited the growth of DU145 (p53^p223L/V274F^) and PC-3 (p53^R273H^); Figure S4: *MDM4* targeting agent XI-011, inhibited the in vitro growth of prostate cancer cell lines; Figure S5: *MDM4* KD does not induce senescence in DU145 in vitro; Figure S6: *MDM4* KD does not induce caspase 3 and caspase 7 activation in PC-3 (p53^R273H)^ PC cell line; Figure S7: p53 immunofluorescence staining of PC-3 (p53^R273H^) and treatment efficacy of the combination treatment *MDM4* KD and eprenetapopt in PC-3 prostate cancer isogenic cell lines; Figure S8: Raw Western blot data of Figure 2; Figure S9: Raw Western blot, apoptosis positive controls. SKBr3 treated with doxorubicin for 24 h; Figure S10: Raw Western blot data of Figure 3; Figure S11: Raw Western blot data of Figure 4; Figure S12: Raw Western blot data of Figure 5; Figure S13: Raw Western blot data of Figure 6; Figure S14: Raw Western blot data of Figure S4; Figure S15: Raw Western blot data of Figure S6; Table S1: PC patient sample immunohistochemistry (IHC) scoring; Table S2: Epenetapopt IC_50_ values for prostate cancer (PC) cell lines.

## References

[1] Sandhu S., Moore C.M., Chiong E., Beltran H., Bristow R.G., Williams S.G. (2021). Prostate cancer. Lancet.

[2] Risdon E.N., Chau C.H., Price D.K., Sartor O., Figg W.D. (2021). PARP Inhibitors and Prostate Cancer: To Infinity and Beyond BRCA. Oncologist.

[3] Lang G.A., Iwakuma T., Suh Y.A., Liu G., Rao V.A., Parant J.M., Valentin-Vega Y.A., Terzian T., Caldwell L.C., Strong L.C. (2004). Gain of function of a p53 hot spot mutation in a mouse model of Li-Fraumeni syndrome. Cell.

[4] Olive K.P., Tuveson D.A., Ruhe Z.C., Yin B., Willis N.A., Bronson R.T., Crowley D., Jacks T. (2004). Mutant p53 gain of function in two mouse models of Li-Fraumeni syndrome. Cell.

[5] Powell E., Piwnica-Worms D., Piwnica-Worms H. (2014). Contribution of p53 to metastasis. Cancer Discov..

[6] Weissmueller S., Manchado E., Saborowski M., Morris JPt Wagenblast E., Davis C.A., Moon S.H., Pfister N.T., Tschaharganeh D.F., Kitzing T., Aust D. (2014). Mutant p53 drives pancreatic cancer metastasis through cell-autonomous PDGF receptor beta signaling. Cell.

[7] Muller P.A., Vousden K.H. (2014). Mutant p53 in cancer: New functions and therapeutic opportunities. Cancer Cell.

[8] Hong M.K., Macintyre G., Wedge D.C., Van Loo P., Patel K., Lunke S., Alexandrov L.B., Sloggett C., Cmero M., Marass F. (2015). Tracking the origins and drivers of subclonal metastatic expansion in prostate cancer. Nat. Commun..

[9] Gundem G., Van Loo P., Kremeyer B., Alexandrov L.B., Tubio J.M., Papaemmanuil E., Brewer D.S., Kallio H.M., Hognas G., Annala M. (2015). The evolutionary history of lethal metastatic prostate cancer. Nature.

[10] Ku S.Y., Rosario S., Wang Y., Mu P., Seshadri M., Goodrich Z.W., Goodrich M.M., Labbe D.P., Gomez E.C., Wang J. (2017). Rb1 and Trp53 cooperate to suppress prostate cancer lineage plasticity, metastasis, and antiandrogen resistance. Science.

[11] Gesztes W., Schafer C., Young D., Fox J., Jiang J., Chen Y., Kuo H.C., Mwamukonda K.B., Dobi A., Burke A.P. (2022). Focal p53 protein expression and lymphovascular invasion in primary prostate tumors predict metastatic progression. Sci. Rep..

[12] Quinn D.I., Stricker P.D., Kench J.G., Grogan J., Haynes A.M., Henshall S.M., Grygiel J.J., Delprado W., Turner J.J., Horvath L.G. (2019). p53 nuclear accumulation as an early indicator of lethal prostate cancer. Br. J. Cancer.

[13] Teroerde M., Nientiedt C., Duensing A., Hohenfellner M., Stenzinger A., Duensing S., Bott S.R.J., Ng K.L. (2021). Revisiting the Role of p53 in Prostate Cancer. Prostate Cancer.

[14] Vousden K.H., Lane D.P. (2007). p53 in health and disease. Nat. Rev. Mol. Cell Biol..

[15] Haupt S., Mejia-Hernandez J.O., Vijayakumaran R., Keam S.P., Haupt Y. (2019). The long and the short of it: The MDM4 tail so far. J. Mol. Cell Biol..

[16] Klein A.M., de Queiroz R.M., Venkatesh D., Prives C. (2021). The roles and regulation of MDM2 and MDMX: It is not just about p53. Genes Dev..

[17] Haupt Y., Maya R., Kazaz A., Oren M. (1997). Mdm2 promotes the rapid degradation of p53. Nature.

[18] Kubbutat M.H., Jones S.N., Vousden K.H. (1997). Regulation of p53 stability by Mdm2. Nature.

[19] Haupt S., Buckley D., Pang J.-M.B., Panimaya J., Paul P.J., Gamel C., Takano E.A., Lee Y.-Y., Hidding S., Rogers T.-M. (2015). Targeting Mdmx to treat Breast Cancers with wild type p53. Cell Death Dis..

[20] Burgess A., Chia K.M., Haupt S., Thomas D., Haupt Y., Lim E. (2016). Clinical Overview of MDM2/X-Targeted Therapies. Front. Oncol..

[21] Garcia D., Warr M.R., Martins C.P., Brown Swigart L., Passegue E., Evan G.I. (2011). Validation of MdmX as a therapeutic target for reactivating p53 in tumors. Genes Dev..

[22] De Lange J., Teunisse A.F., Vries M.V., Lodder K., Lam S., Luyten G.P., Bernal F., Jager M.J., Jochemsen A.G. (2012). High levels of Hdmx promote cell growth in a subset of uveal melanomas. Am. J. Cancer Res..

[23] Gembarska A., Luciani F., Fedele C., Russell E.A., Dewaele M., Villar S., Zwolinska A., Haupt S., de Lange J., Yip D. (2012). MDM4 is a key therapeutic target in cutaneous melanoma. Nat. Med..

[24] Yu D.H., Xu Z.Y., Mo S., Yuan L., Cheng X.D., Qin J.J. (2020). Targeting MDMX for Cancer Therapy: Rationale, Strategies, and Challenges. Front. Oncol..

[25] Miranda P.J., Buckley D., Raghu D., Pang J.B., Takano E.A., Vijayakumaran R., Teunisse A.F., Posner A., Procter T., Herold M.J. (2017). MDM4 is a rational target for treating breast cancers with mutant p53. J. Pathol..

[26] Herold M.J., van den Brandt J., Seibler J., Reichardt H.M. (2008). Inducible and reversible gene silencing by stable integration of an shRNA-encoding lentivirus in transgenic rats. Proc. Natl. Acad. Sci. USA.

[27] Wang H., Ma X., Ren S., Buolamwini J.K., Yan C. (2011). A small-molecule inhibitor of MDMX activates p53 and induces apoptosis. Mol. Cancer Ther..

[28] Czekanska E.M. (2011). Assessment of cell proliferation with resazurin-based fluorescent dye. Methods Mol. Biol..

[29] Page B., Page M., Noel C. (1993). A new fluorometric assay for cytotoxicity measurements in-vitro. Int. J. Oncol..

[30] Dimri G.P., Lee X., Basile G., Acosta M., Scott G., Roskelley C., Medrano E.E., Linskens M., Rubelj I., Pereira-Smith O. (1995). A biomarker that identifies senescent human cells in culture and in aging skin in vivo. Proc. Natl. Acad. Sci. USA.

[31] Louria-Hayon I., Grossman T., Sionov R.V., Alsheich O., Pandolfi P.P., Haupt Y. (2003). The promyelocytic leukemia protein protects p53 from Mdm2-mediated inhibition and degradation. J. Biol. Chem..

[32] Livak K.J., Schmittgen T.D. (2001). Analysis of relative gene expression data using real-time quantitative PCR and the 2(-Delta Delta C(T)) Method. Methods.

[33] Paul P.J., Raghu D., Chan A.L., Gulati T., Lambeth L., Takano E., Herold M.J., Hagekyriakou J., Vessella R.L., Fedele C. (2016). Restoration of tumor suppression in prostate cancer by targeting the E3 ligase E6AP. Oncogene.

[34] Robinson D., Van Allen E.M., Wu Y.M., Schultz N., Lonigro R.J., Mosquera J.M., Montgomery B., Taplin M.E., Pritchard C.C., Attard G. (2015). Integrative clinical genomics of advanced prostate cancer. Cell.

[35] Haupt S., Vijayakumaran R., Panimaya J., Burgess A., Lim E., Haupt Y. (2017). The role of MDM2 and MDM4 in breast cancer development and prevention. J. Mol. Cell Biol..

[36] Alsop K., Thorne H., Sandhu S., Hamilton A., Mintoff C., Christie E., Spruyt O., Williams S., McNally O., Mileshkin L. (2016). A community-based model of rapid autopsy in end-stage cancer patients. Nat. Biotechnol..

[37] Grasso C.S., Wu Y.M., Robinson D.R., Cao X., Dhanasekaran S.M., Khan A.P., Quist M.J., Jing X., Lonigro R.J., Brenner J.C. (2012). The mutational landscape of lethal castration-resistant prostate cancer. Nature.

[38] Stegeman S., Moya L., Selth L.A., Spurdle A.B., Clements J.A., Batra J. (2015). A genetic variant of MDM4 influences regulation by multiple microRNAs in prostate cancer. Endocr. Relat. Cancer.

[39] Vichai V., Kirtikara K. (2006). Sulforhodamine B colorimetric assay for cytotoxicity screening. Nat. Protoc..

[40] McCann J.J., Vasilevskaya I.A., McNair C., Gallagher P., Neupane N.P., de Leeuw R., Shafi A.A., Dylgjeri E., Mandigo A.C., Schiewer M.J. (2022). Mutant p53 elicits context-dependent pro-tumorigenic phenotypes. Oncogene.

[41] Roh J.L., Park J.Y., Kim E.H. (2014). XI-011 enhances cisplatin-induced apoptosis by functional restoration of p53 in head and neck cancer. Apoptosis.

[42] Liu Y., Gu W. (2022). p53 in ferroptosis regulation: The new weapon for the old guardian. Cell Death Differ..

[43] Venkatesh D., O’Brien N.A., Zandkarimi F., Tong D.R., Stokes M.E., Dunn D.E., Kengmana E.S., Aron A.T., Klein A.M., Csuka J.M. (2020). MDM2 and MDMX promote ferroptosis by PPARalpha-mediated lipid remodeling. Genes Dev..

[44] Molnar T., Pallagi P., Tel B., Kiraly R., Csoma E., Jenei V., Varga Z., Gogolak P., Odile Hueber A., Mate Z. (2021). Caspase-9 acts as a regulator of necroptotic cell death. FEBS J..

[45] Miller K.R., Kelley K., Tuttle R., Berberich S.J. (2010). HdmX overexpression inhibits oncogene induced cellular senescence. Cell Cycle.

[46] Kortlever R.M., Higgins P.J., Bernards R. (2006). Plasminogen activator inhibitor-1 is a critical downstream target of p53 in the induction of replicative senescence. Nat. Cell Biol..

[47] Xu S., Wu W., Huang H., Huang R., Xie L., Su A., Liu S., Zheng R., Yuan Y., Zheng H.L. (2019). The p53/miRNAs/Ccna2 pathway serves as a novel regulator of cellular senescence: Complement of the canonical p53/p21 pathway. Aging Cell.

[48] Zhao H., Bauzon F., Fu H., Lu Z., Cui J., Nakayama K., Nakayama K.I., Locker J., Zhu L. (2013). Skp2 deletion unmasks a p27 safeguard that blocks tumorigenesis in the absence of pRb and p53 tumor suppressors. Cancer Cell.

[49] Birsen R., Larrue C., Decroocq J., Johnson N., Guiraud N., Gotanegre M., Cantero-Aguilar L., Grignano E., Huynh T., Fontenay M. (2022). APR-246 induces early cell death by ferroptosis in acute myeloid leukemia. Haematologica.

[50] Fujihara K.M., Zhang B., Jackson T.D., Nijiagel B., Ang C., Nikolic I., Sutton V., Trapani J., Simpson K.J. (2022). Stojanovski, D.; et al. Eprenetapopt triggers ferroptosis, inhibits NFS1 cysteine desulfurase and synergizes with serine and glycine dietary restriction. Sci. Adv..

[51] Liu D.S., Duong C.P., Haupt S., Montgomery K.G., House C.M., Azar W.J., Pearson H.B., Fisher O.M., Read M., Guerra G.R. (2017). Inhibiting the system xC(-)/glutathione axis selectively targets cancers with mutant-p53 accumulation. Nat. Commun..

[52] Fujihara K.M., Corrales Benitez M., Cabalag C.S., Zhang B.Z., Ko H.S., Liu D.S., Simpson K.J., Haupt Y., Lipton L., Haupt S. (2021). SLC7A11 Is a Superior Determinant of APR-246 (Eprenetapopt) Response than TP53 Mutation Status. Mol. Cancer Ther..

[53] Lehmann S., Bykov V.J., Ali D., Andren O., Cherif H., Tidefelt U., Uggla B., Yachnin J., Juliusson G., Moshfegh A. (2012). Targeting p53 in vivo: A first-in-human study with p53-targeting compound APR-246 in refractory hematologic malignancies and prostate cancer. J. Clin. Oncol..

[54] Toledo F., Wahl G.M. (2006). Regulating the p53 pathway: In vitro hypotheses, in vivo veritas. Nat. Rev. Cancer.

[55] Cetintas V.B., Batada N.N. (2020). Is there a causal link between PTEN deficient tumors and immunosuppressive tumor microenvironment?. J. Transl. Med..

[56] Danovi D., Meulmeester E., Pasini D., Migliorini D., Capra M., Frenk R., de Graaf P., Francoz S., Gasparini P., Gobbi A. (2004). Amplification of Mdmx (or Mdm4) directly contributes to tumor formation by inhibiting p53 tumor suppressor activity. Mol. Cell Biol..

[57] Carrillo A.M., Bouska A., Arrate M.P., Eischen C.M. (2015). Mdmx promotes genomic instability independent of p53 and Mdm2. Oncogene.

[58] Wohlberedt K., Klusmann I., Derevyanko P.K., Henningsen K., Choo J., Manzini V., Magerhans A., Giansanti C., Eischen C.M., Jochemsen A.G. (2020). Mdm4 supports DNA replication in a p53-independent fashion. Oncogene.

[59] Gao C., Xiao G., Piersigilli A., Gou J., Ogunwobi O., Bargonetti J. (2019). Context-dependent roles of MDMX (MDM4) and MDM2 in breast cancer proliferation and circulating tumor cells. Breast Cancer Res..

[60] Xiong S., Pant V., Zhang Y., Aryal N.K., You M.J., Kusewitt D., Lozano G. (2017). The p53 inhibitor Mdm4 cooperates with multiple genetic lesions in tumourigenesis. J. Pathol..

[61] Uchida C., Miwa S., Isobe T., Kitagawa K., Hattori T., Oda T., Yasuda H., Kitagawa M. (2006). Effects of MdmX on Mdm2-mediated downregulation of pRB. FEBS Lett..

[62] Simeckova S., Kahounova Z., Fedr R., Remsik J., Slabakova E., Suchankova T., Prochazkova J., Bouchal J., Kharaishvili G., Kral M. (2019). High Skp2 expression is associated with a mesenchymal phenotype and increased tumorigenic potential of prostate cancer cells. Sci. Rep..

[63] Liu D.S., Read M., Cullinane C., Azar W.J., Fennell C.M., Montgomery K.G., Haupt S., Haupt Y., Wiman K.G., Duong C.P. (2015). APR-246 potently inhibits tumour growth and overcomes chemoresistance in preclinical models of oesophageal adenocarcinoma. Gut.

[64] Jyotsana N., Ta K.T., DelGiorno K.E. (2022). The Role of Cystine/Glutamate Antiporter SLC7A11/xCT in the Pathophysiology of Cancer. Front. Oncol..

[65] Chen Y., Wang D.D., Wu Y.P., Su D., Zhou T.Y., Gai R.H., Fu Y.Y., Zheng L., He Q.J., Zhu H. (2017). MDM2 promotes epithelial-mesenchymal transition and metastasis of ovarian cancer SKOV3 cells. Br. J. Cancer.

[66] Bykov V.J., Zhang Q., Zhang M., Ceder S., Abrahmsen L., Wiman K.G. (2016). Targeting of Mutant p53 and the Cellular Redox Balance by APR-246 as a Strategy for Efficient Cancer Therapy. Front. Oncol..

[67] Lin W., Wang C., Liu G., Bi C., Wang X., Zhou Q., Jin H. (2020). SLC7A11/xCT in cancer: Biological functions and therapeutic implications. Am. J. Cancer Res..

[68] Wu J., Lu G., Wang X. (2021). MDM4 alternative splicing and implication in MDM4 targeted cancer therapies. Am. J. Cancer Res..

[69] Pardieu B., Pasanisi J., Ling F., Dal Bello R., Penneroux J., Su A., Joudinaud R., Chat L., Wu H.C., Duchmann M. (2022). Cystine uptake inhibition potentiates front-line therapies in acute myeloid leukemia. Leukemia.

[70] Ceder S., Eriksson S.E., Cheteh E.H., Dawar S., Corrales Benitez M., Bykov V.J.N., Fujihara K.M., Grandin M., Li X., Ramm S. (2021). A thiol-bound drug reservoir enhances APR-246-induced mutant p53 tumor cell death. EMBO Mol. Med..

