# Supplementary material for: Targeting MDM4 as a Novel Therapeutic Approach in Prostate Cancer Independent of p53 Status
Source: Cancers (Basel). 2022 Aug 16;14(16):3947. doi: 10.3390/cancers14163947 (PMC9405814; doi:10.3390/cancers14163947)
Supplement: Supplementary file 1 [file cancers-14-03947-s001.zip › cancers-1838033-supplementary.pdf]

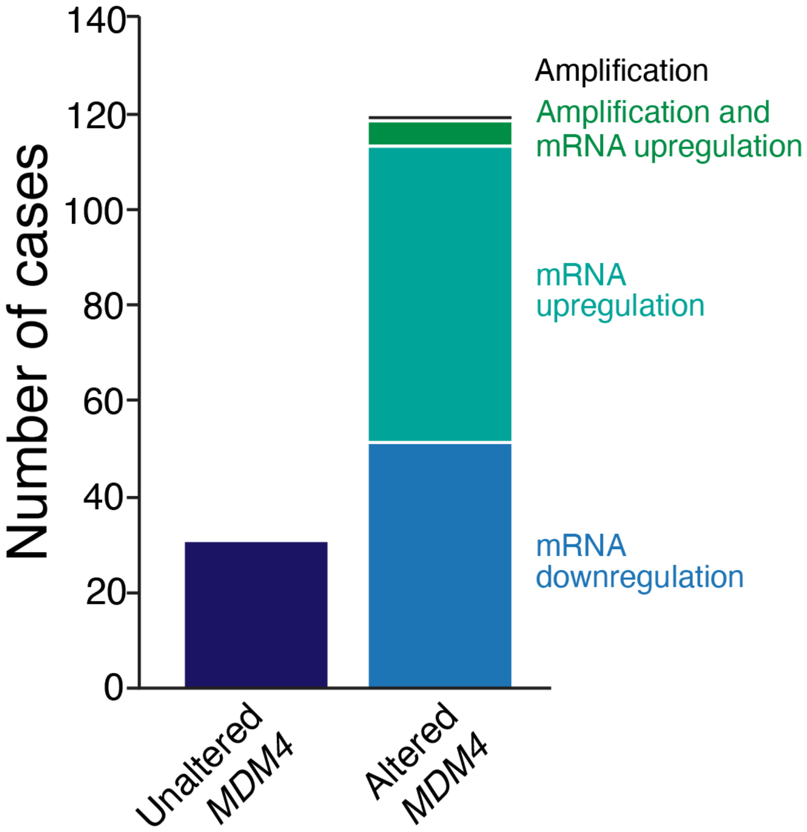

Figure S1. ***MDM4*** **alteration frequency in metastatic prostate cancer.** Alteration frequency of MDM4 in metastatic dataset from CBioPortal.

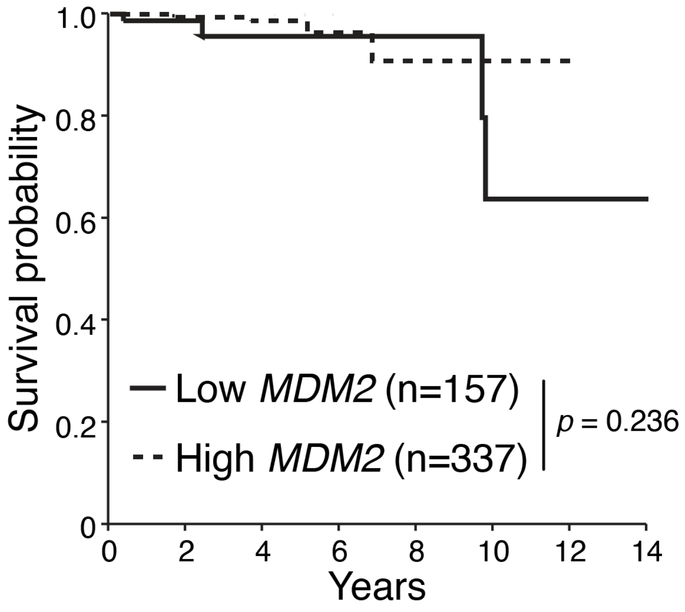

Figure S2. ***MDM2*** **levels and their impact on prostate cancer patient survival.** Kaplan-Meier plot for PC patients expressing either low or high *MDM2* mRNA levels as a function of survival probability. Statistical significance was calculated using Log-rank (Mantel-Cox) test.

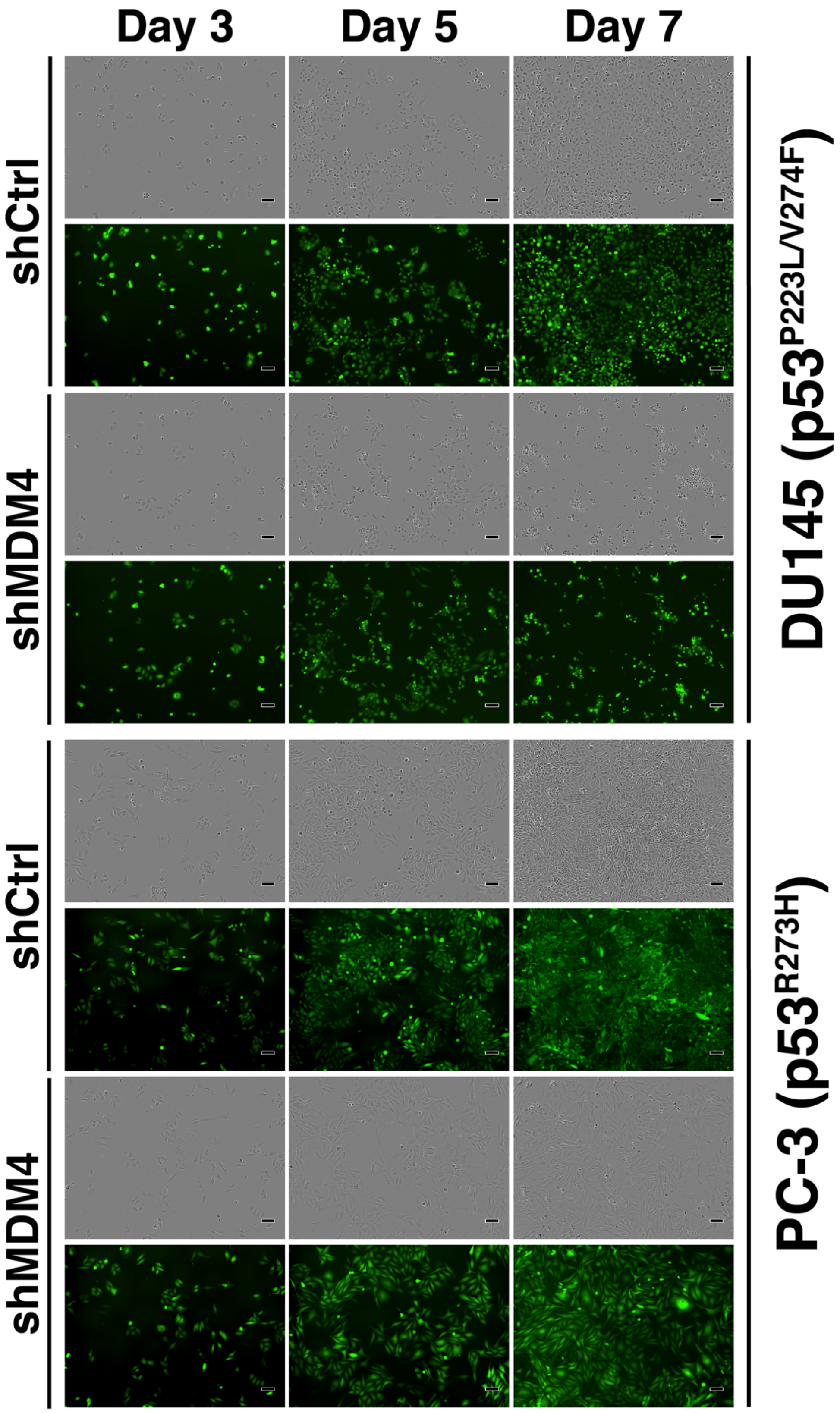

Figure S3. ***MDM4*** **knockdown inhibited the growth of DU145 (p53$^{P223L/V274F}$) and PC-3 (p53^{R273H}).** Representative phase-contrast and fluorescence microscopy images of mutant p53 and GPF-tagged PC cell lines treated with Doxycycline (Doxy; 25ng/mL) over a period of 7 days. Scale bars indicate 100µm in all cases.

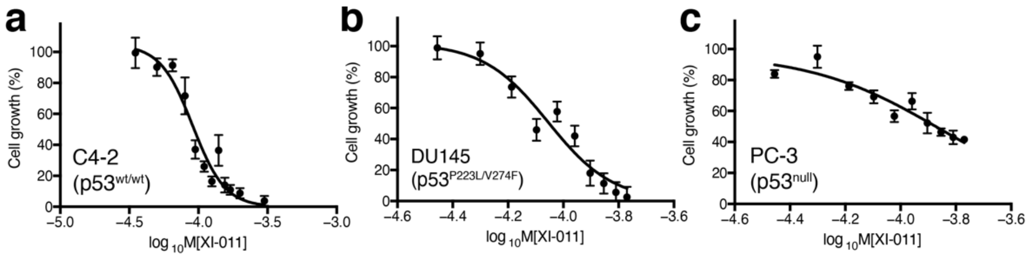

d

PC cell lines	XI-011 IC_{50} (nM) ± SEM determined using SRB assay
C4-2 ($p53^{wt/wt}$)	98.78 ± 42
DU145 ($p53^{P223L/V247F}$)	90.39 ± 12
PC-3 ($p53^{null}$)	138.4 ± 24

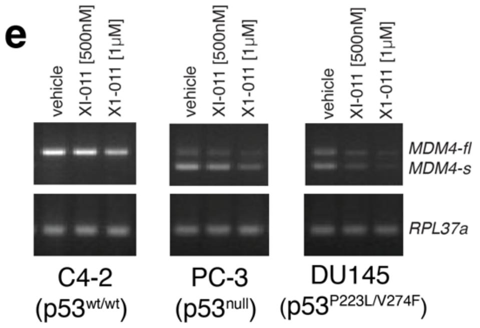

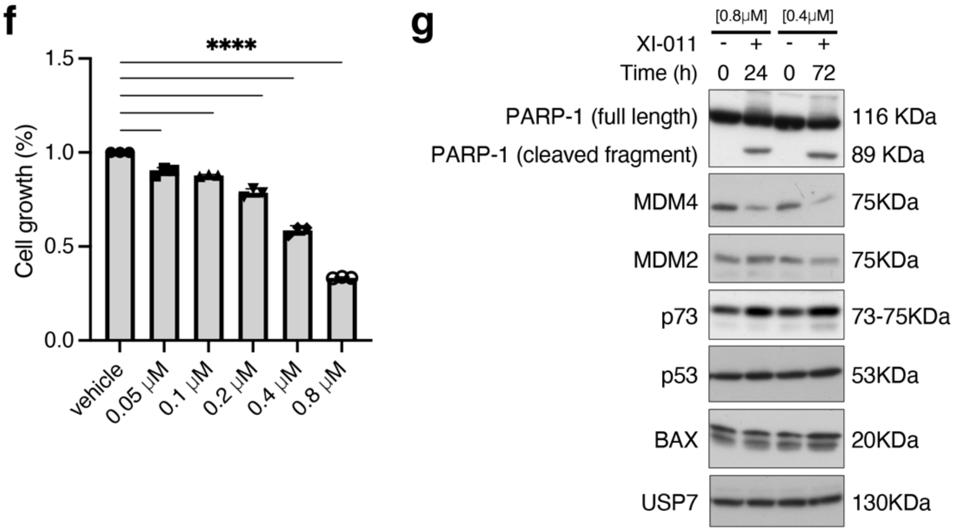

Figure S4. MDM4 targeting agent XI-011, inhibited the *in vitro* growth of prostate cancer cell lines. **(a-c)** Three PC cell lines C4-2 ($p53^{wt/wt}$), DU145 ($p53^{P223L/V274F}$), and PC-3 ($p53^{null}$) were treated with XI-011 for a period of 96 hours and the effects over cell numbers were assessed using SRB assay. **(d)** The table shows the XI-011 IC_{50} values determined for each PC cell line. **(e)** Reduction of *MDM4* levels in response to XI-011 after 24h was evident as demonstrated for C4-2 ($p53^{wt/wt}$), DU145 ($p53^{P223L/V274F}$) and PC-3 ($p53^{null}$) using two different concentrations of XI-011 (500nM and 1uM). **(f)** The graph showing the relative survival of VCaP after XI-011 96 hours treatment assessed by CellTiter-Blue. Data shown as mean ± SEM of biological replicates (n=3). Statistical significance was calculated using ANOVA and Tukey's tests (*p≤0.05, **p≤0.01, ***p≤0.001, ****p≤0.0001). **(g)** VCaP PC cells were treated with XI-011 and collected for exploring the protein expression levels using Western blot.

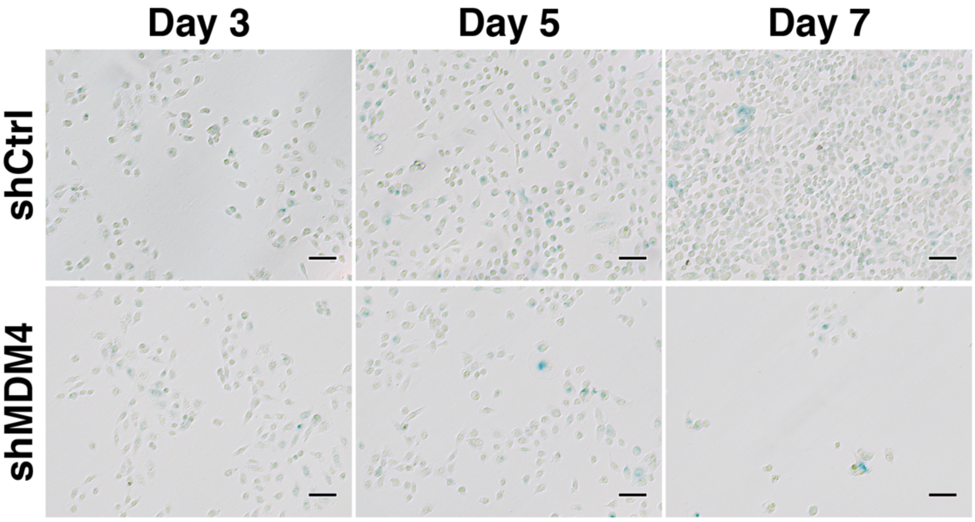

Figure S5. ***MDM4*** **KD does not induce senescence in DU145** ***in vitro*****.** MDM4 inhibition does not cause senescence in DU145 as revealed by Senescence-associated β-galactosidase (SA-β-gal) staining at pH 6 on day 3, day 5, and day 7. SA-β-gal-positive cells stains in blue. Scale bars indicate 100μm.

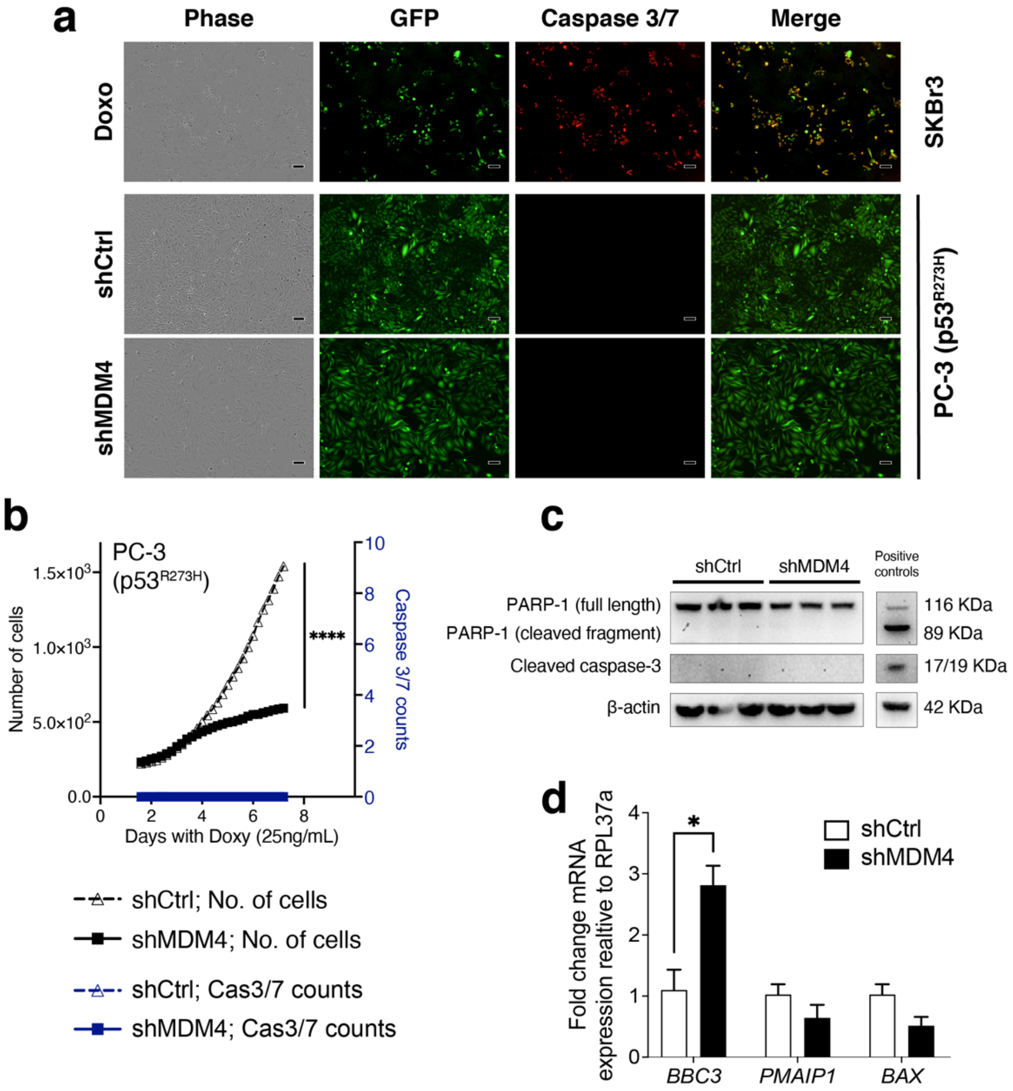

Figure S6. ***MDM4*** **KD does not induce caspase 3 and caspase 7 activation in PC-3 ($p53^{R273H}$) PC cell line.** SKBr3 cells (GFP tagged) were treated with 20µM of doxorubicin and used as apoptosis-positive control. Either shMDM4 or shCtrl expression was induced with Doxycycline (Doxy; 25ng/mL) in GPF-tagged PC-3 ($p53^{R273H}$) for 7 days. On day 2, cells were treated with Red Incucyte® Caspase-3/7 Dye for detecting apoptosis. **(a)** Representative phase-contrast and fluorescence microscopy images; scale bars indicate 100µm. **(b)** Cell growth rate and kinetic activation of caspase-3/7 were monitored using the live-cell imaging Incucyte® system. **(c)** After MDM4 inhibition, protein was extracted on day 5. Activation of caspase 3 and PARP-1 was explored by Western blot. Each column corresponds to a biological replicate. **(d)** mRNA expression of apoptosis related genes was analysed by RT-qPCR after 5 days of treatment with Doxycycline. mRNA expression levels were normalised to the housekeeping gene *hRLP37a* and expressed relative to shCtrl. Data are shown as mean ± SEM of biological replicates (n=3-6). Statistical significance was calculated using a two-tailed student's t-test (*p≤0.05, **p≤0.01, ***p≤0.001, ****p≤0.0001).

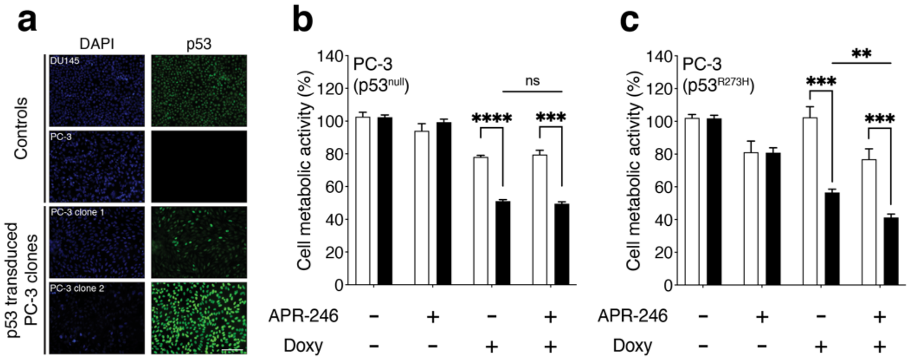

Figure S7. p53 immunofluorescence staining of PC-3 (p53^{R273H}) and treatment efficacy of the combination treatment *MDM4* KD and eprenetapopt in PC-3 prostate cancer isogenic cell lines. (a) Immunofluorescence staining of mutant p53 in PC-3 isogenic clones transduced with the missense p53^{R273H}. DU145 and PC-3 (p53^{null}) were used as controls. DAPI was used to stain the nuclei. Scale bar is 100μm. **(b & c)** To examine whether APR-246 increases the efficacy of MDM4 inhibition, PC-3 isogenic cell lines were treated either with IC_{30} of APR-246 alone or in combination with Doxycycline (Doxy; 25ng/mL) over a period of 5 days (see **Supplementary Table 2**). Treatment response was evaluated by assessing the suppression of cell growth using alamar blue assay. Data are shown as mean ± SEM of biological replicates (n=3). Statistical significance was calculated using a two-tailed student's t-test (*p≤0.05, **p≤0.01, ***p≤0.001, ****p≤0.0001).

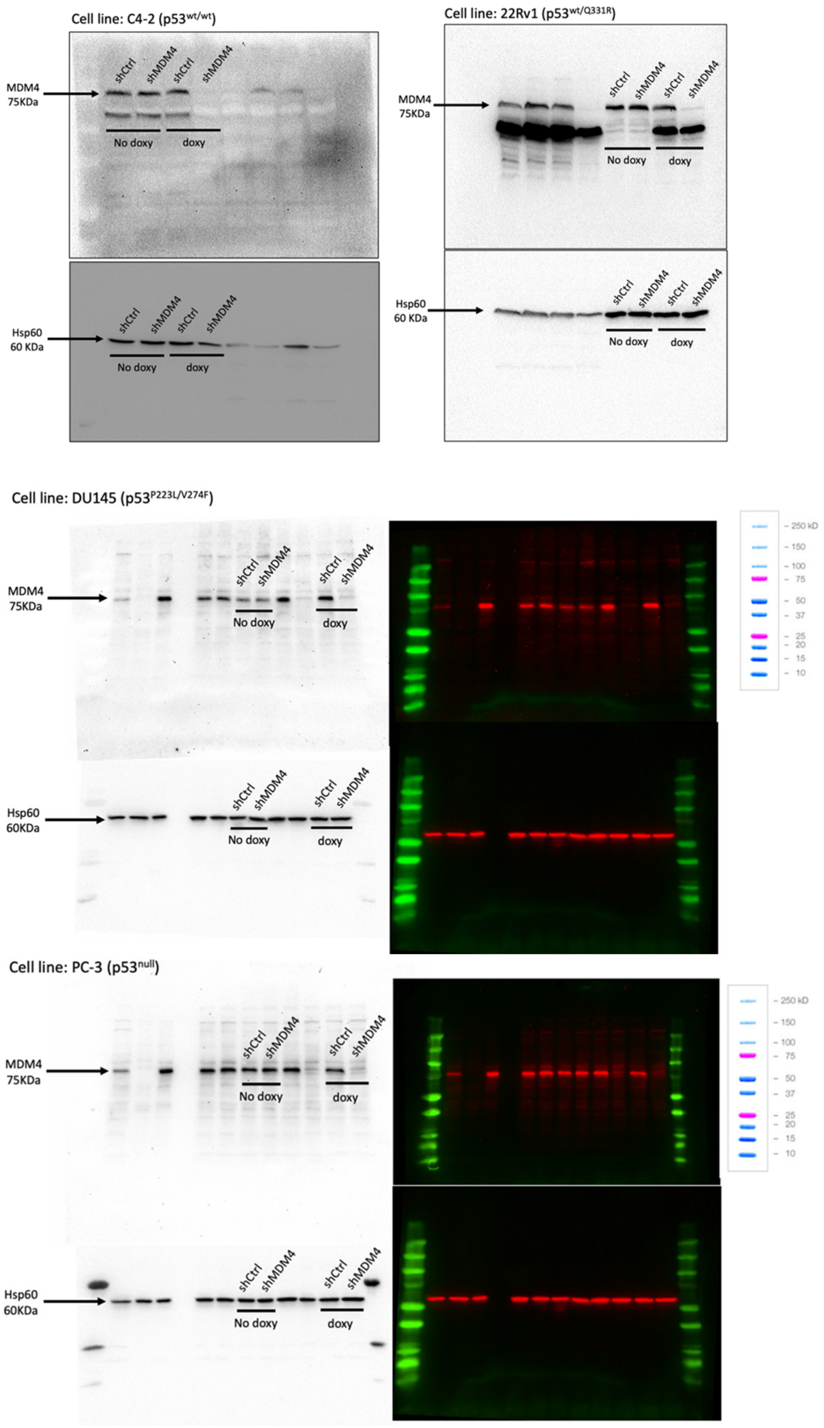

Cell line: C4-2 (p53wt/wt)
MDM4
75KDa
shCtrl
shMDM4
shCtrl
shMDM4
No doxy
doxy
Hsp60
60 KDa
shCtrl
shMDM4
shCtrl
shMDM4
No doxy
doxy
Cell line: 22Rv1 (p53wt/Q331R)
MDM4
75KDa
shCtrl
shMDM4
shCtrl
shMDM4
No doxy
doxy
Hsp60
60 KDa
shCtrl
shMDM4
shCtrl
shMDM4
No doxy
doxy
Cell line: DU145 (p53P223L/V274F)
MDM4
75KDa
shCtrl
shMDM4
shCtrl
shMDM4
No doxy
doxy
Hsp60
60KDa
shCtrl
shMDM4
shCtrl
shMDM4
No doxy
doxy
250 kD
150
100
75
50
37
25
20
15
10
Cell line: PC-3 (p53null)
MDM4
75KDa
shCtrl
shMDM4
shCtrl
shMDM4
No doxy
doxy
Hsp60
60KDa
shCtrl
shMDM4
shCtrl
shMDM4
No doxy
doxy
250 kD
150
100
75
50
37
25
20
15
10

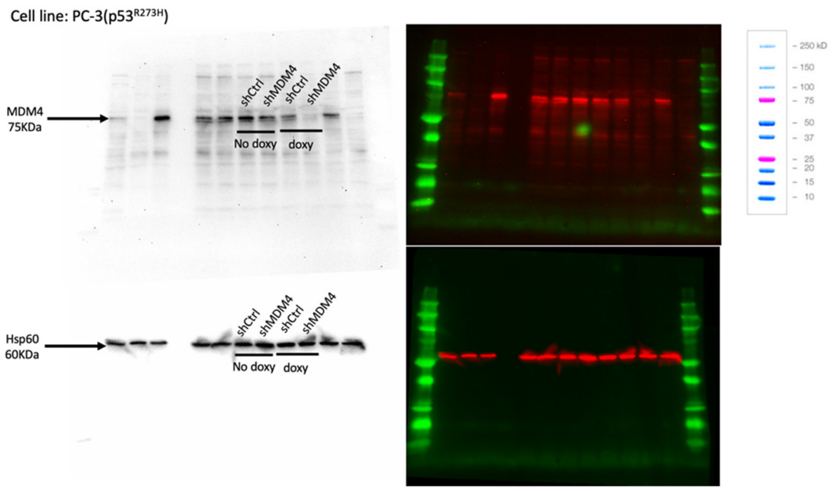

Figure S8. Raw Western blot data of Figure 2

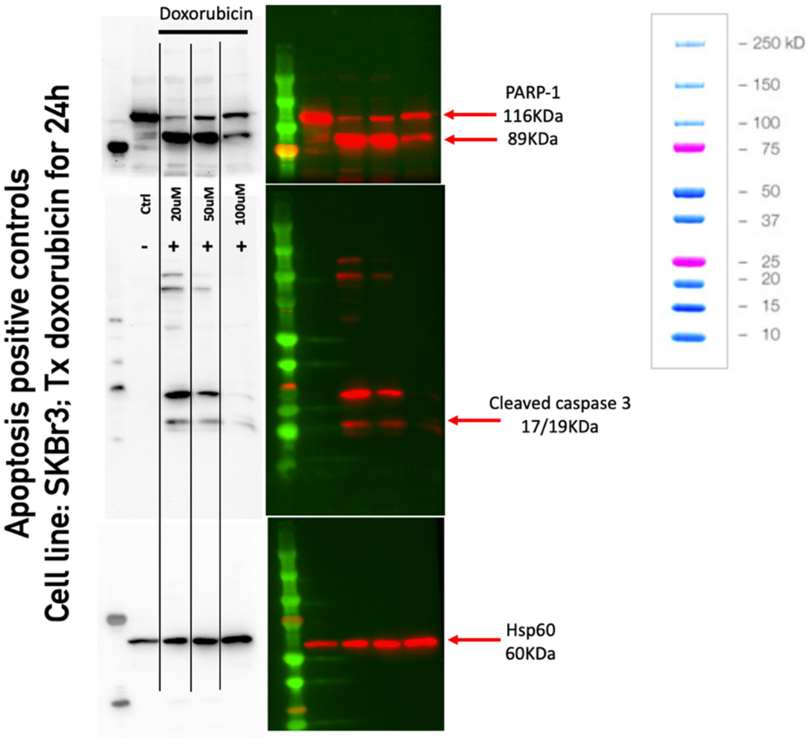

Figure S9. Raw Western blot, apoptosis positive controls. SKBr3 treated with doxorubicin for 24h.

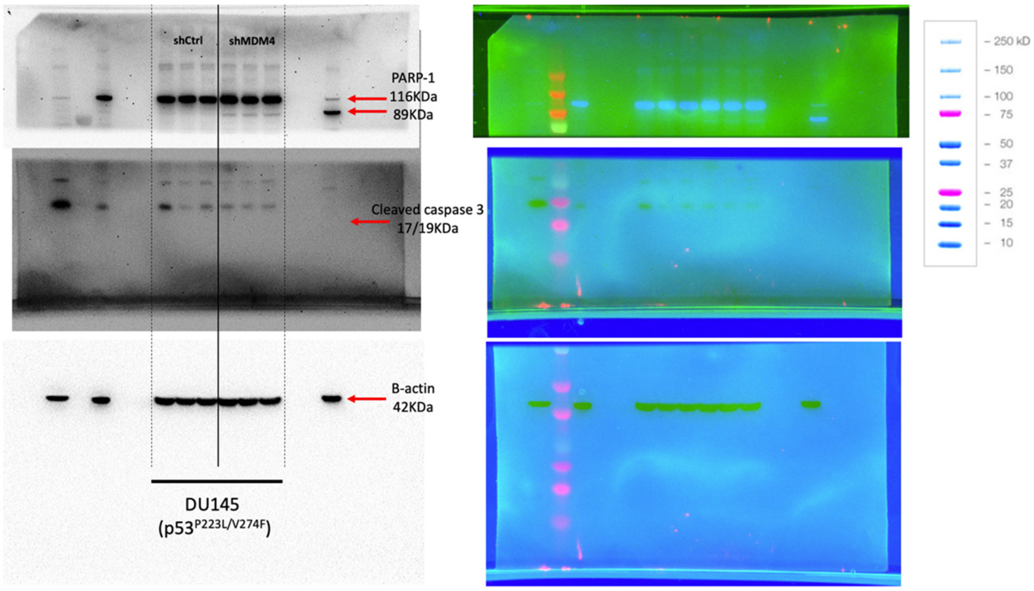

Figure S10. Raw Western blot data of Figure 3.

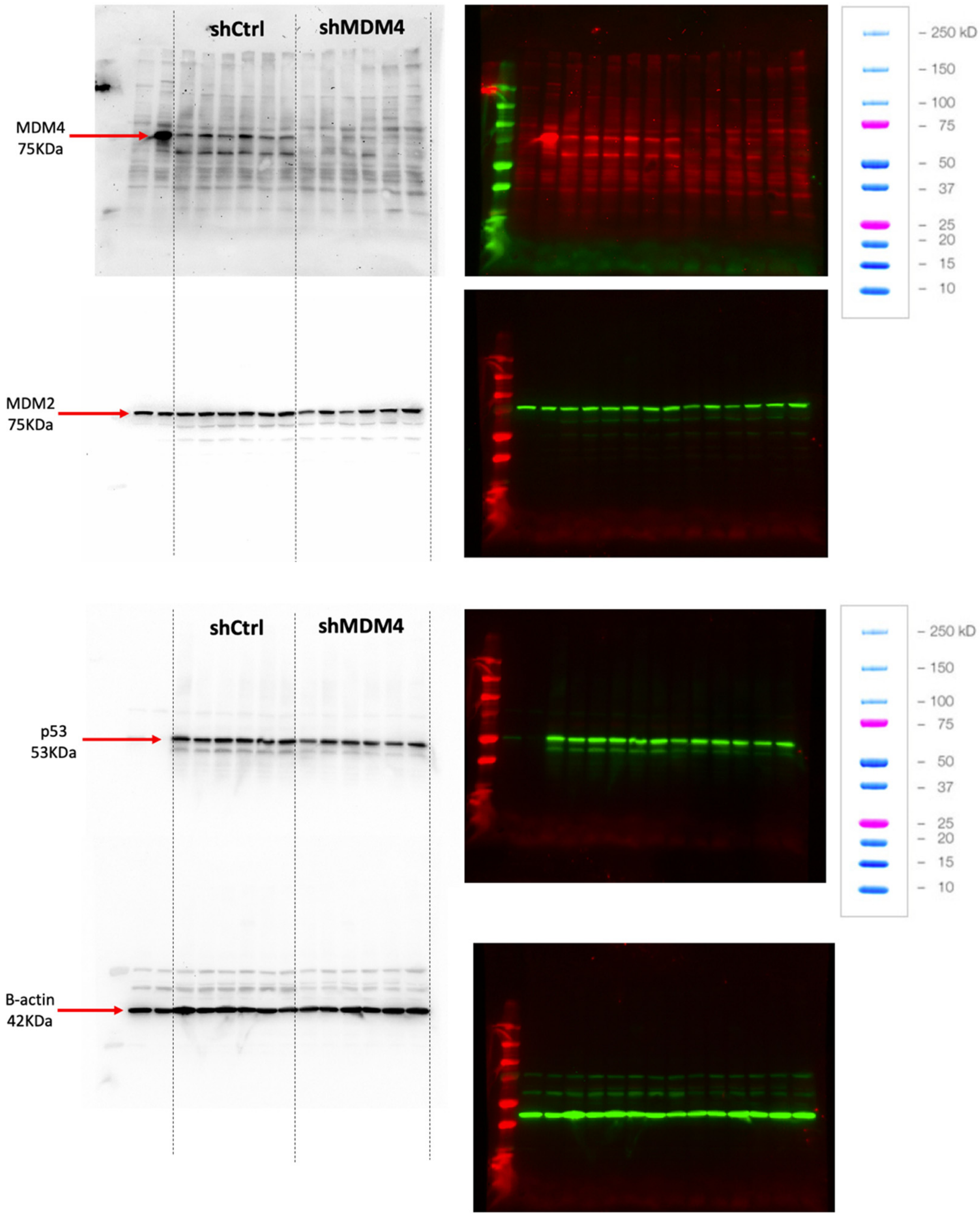

Figure S11. Raw Western blot data of Figure 4.

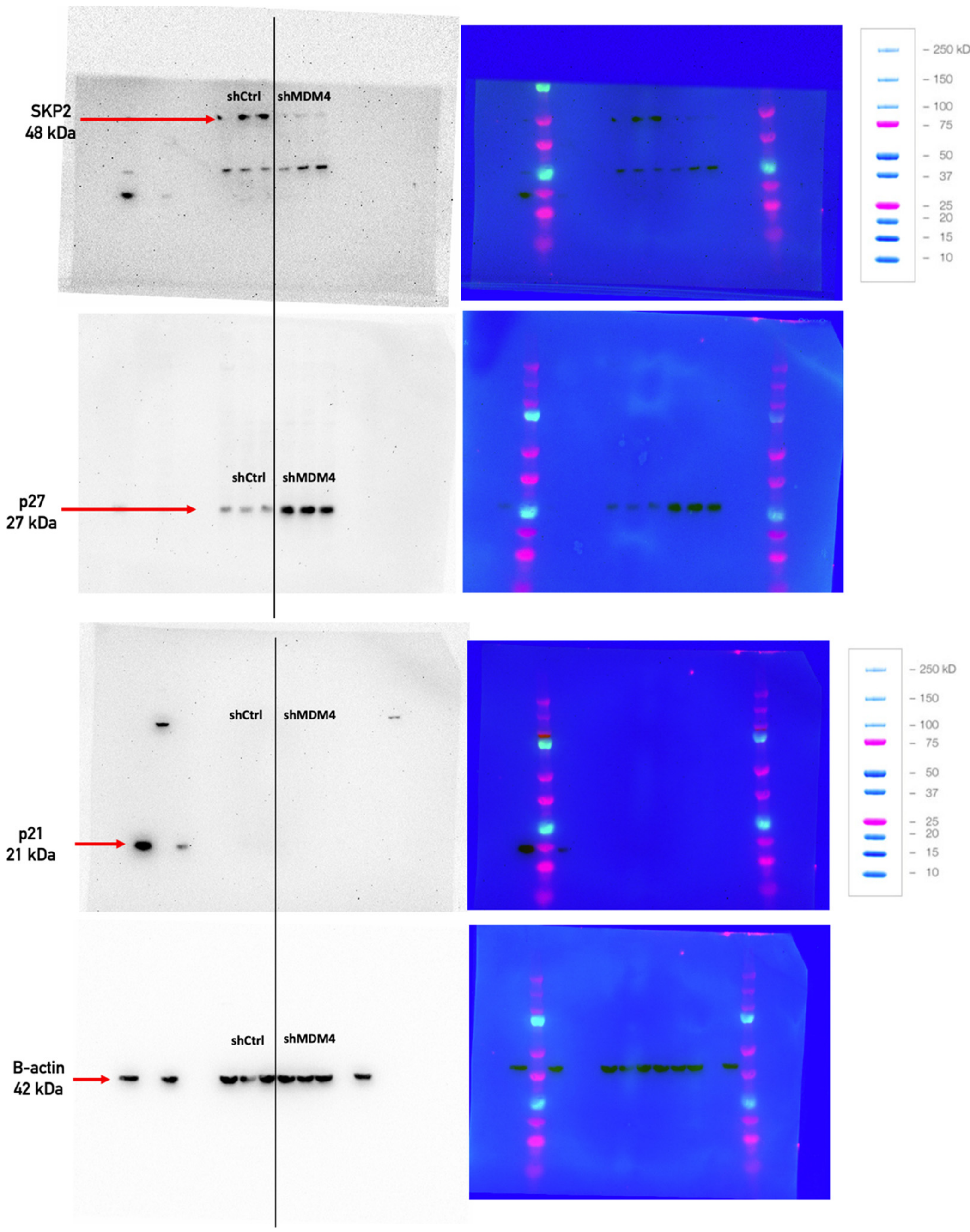

Figure S12. Raw Western blot data of Figure 5.

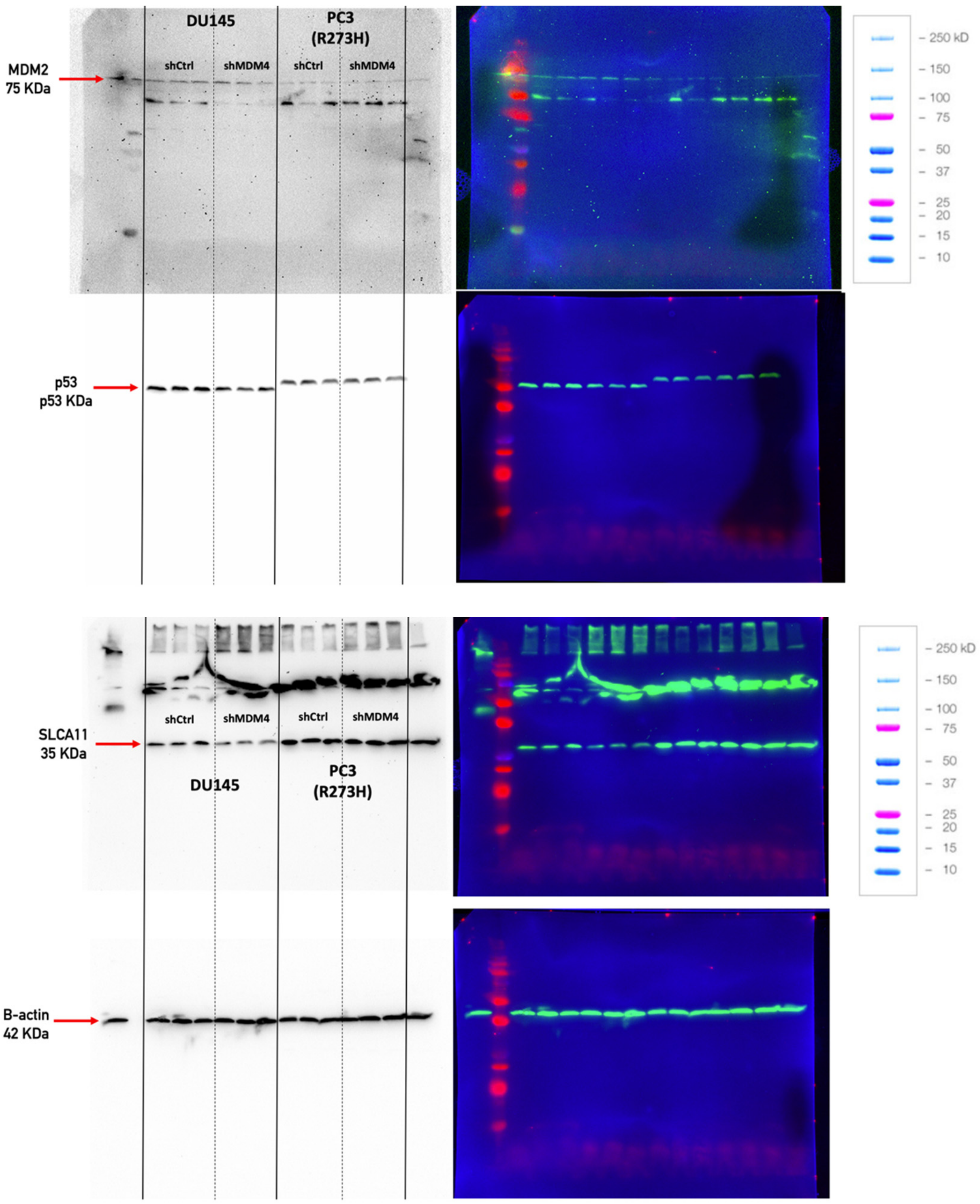

Figure S13. Raw Western blot data of Figure 6.

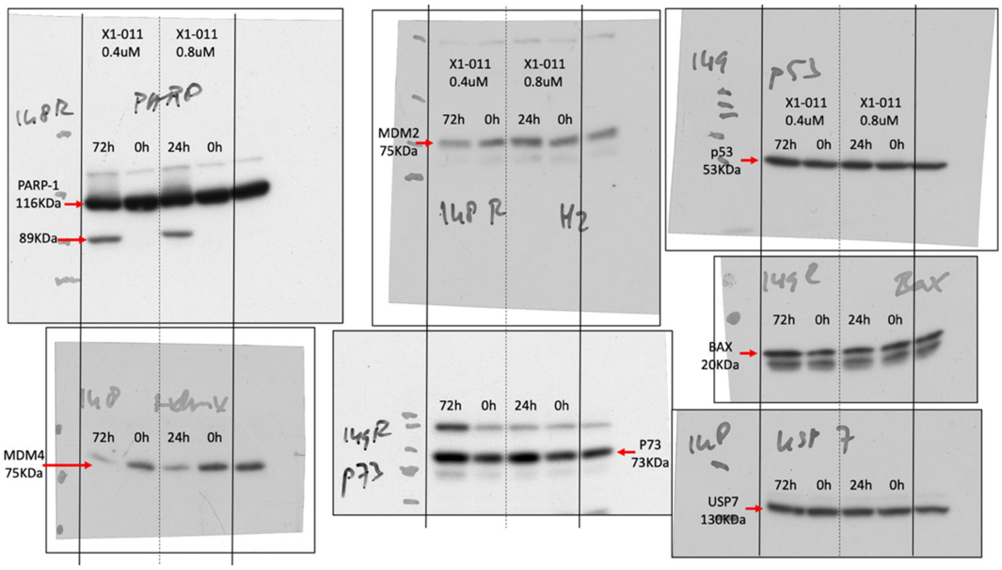

Figure S14. Raw Western blot data of Figure S4.

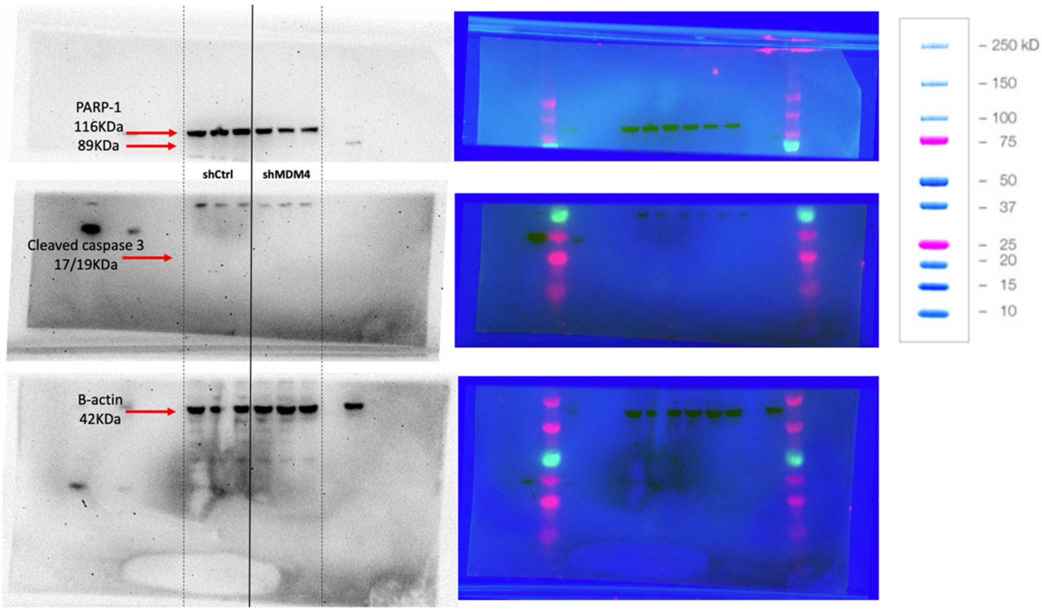

Figure S15. Raw Western blot data of Figure S6.

Table S1. PC patient sample immunohistochemistry (IHC) scoring

Patient	Slide	Tumour sample	IHC histoscore		
			p53	MDM2	MDM4
Patient 1	216	Para-aortic lymph nodes (right)	4	0	7
	222	Prostate - apex left side	4	0	7
	226	Dural base skull	4	0	7
Patient 2	327	Prostate tissue - neck of bladder	0	5	7
	331	Porta-hepatic lymph node	0	2	7
	334	Liver: right lobe A	0	3	7
Patient 3	442	Left para-aortic lymph node track	4	6	6
	444	Bladder base #2	5	3	7
	447	T4/T5 vertebrae	4	7	7
Patient 4	297	Peri-vertebral tumour	7	1	7
	305	Liver: right lobe deposit A	5	1	6
Patient 5	430	Thoracic vertebrae	5	5	7
	431	lymph node - portal	5	2	7
	433	Liver: right lobe nodule 1	5	5	7

Table S2. Epenetapopt IC_{50} values for prostate cancer (PC) cell lines

PC cell lines	Epenetapopt (APR-246) IC_{50} ± SEM determined using Alamar Blue assay
R22v1 ($^{p53wt/Q331R}$)	3.5 ± 0.39
DU145 ($p53^{P223L/V247F}$)	16.3 ± 1.93
PC-3 parental ($p53^{null}$)	32.5 ± 2.90
PC-3 clone 1 ($p53^{R273H}$)	16.3 ± 2.07
PC-3 clone 2 ($p53^{R273H}$)	16.4 ± 1.12
