# Supplementary material for: Targeting MDM4 as a Novel Therapeutic Approach in Prostate Cancer Independent of p53 Status
Source: Cancers (Basel). 2022 Aug 16;14(16):3947. doi: 10.3390/cancers14163947 (PMC9405814; doi:10.3390/cancers14163947)
Supplement: Supplementary file 1 [file cancers-14-03947-s001.zip › Supplementary Materials.docx]

**
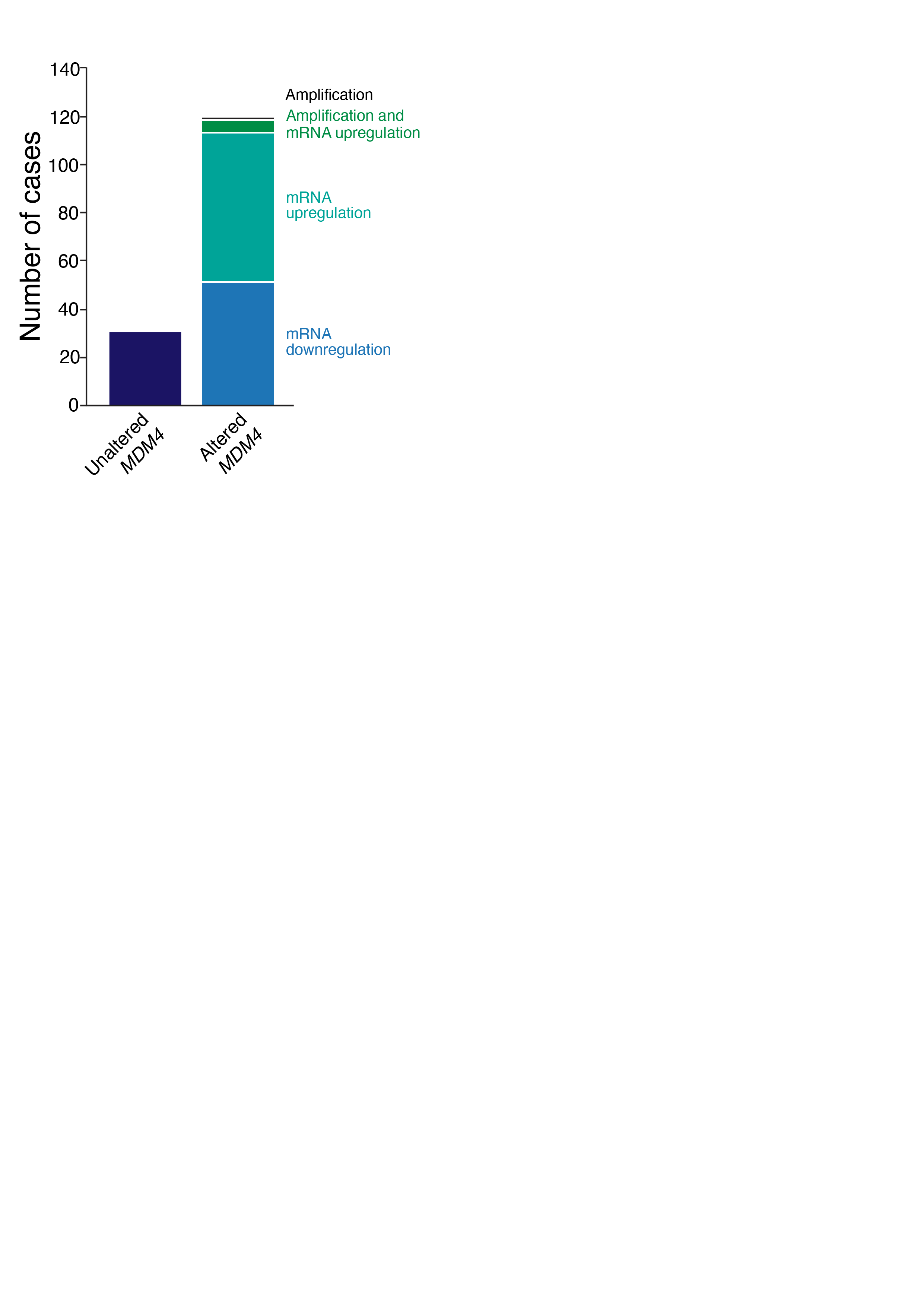
Figure S1. *MDM4* alteration frequency in metastatic prostate cancer.** Alteration frequency of MDM4 in metastatic dataset from CBioPortal.


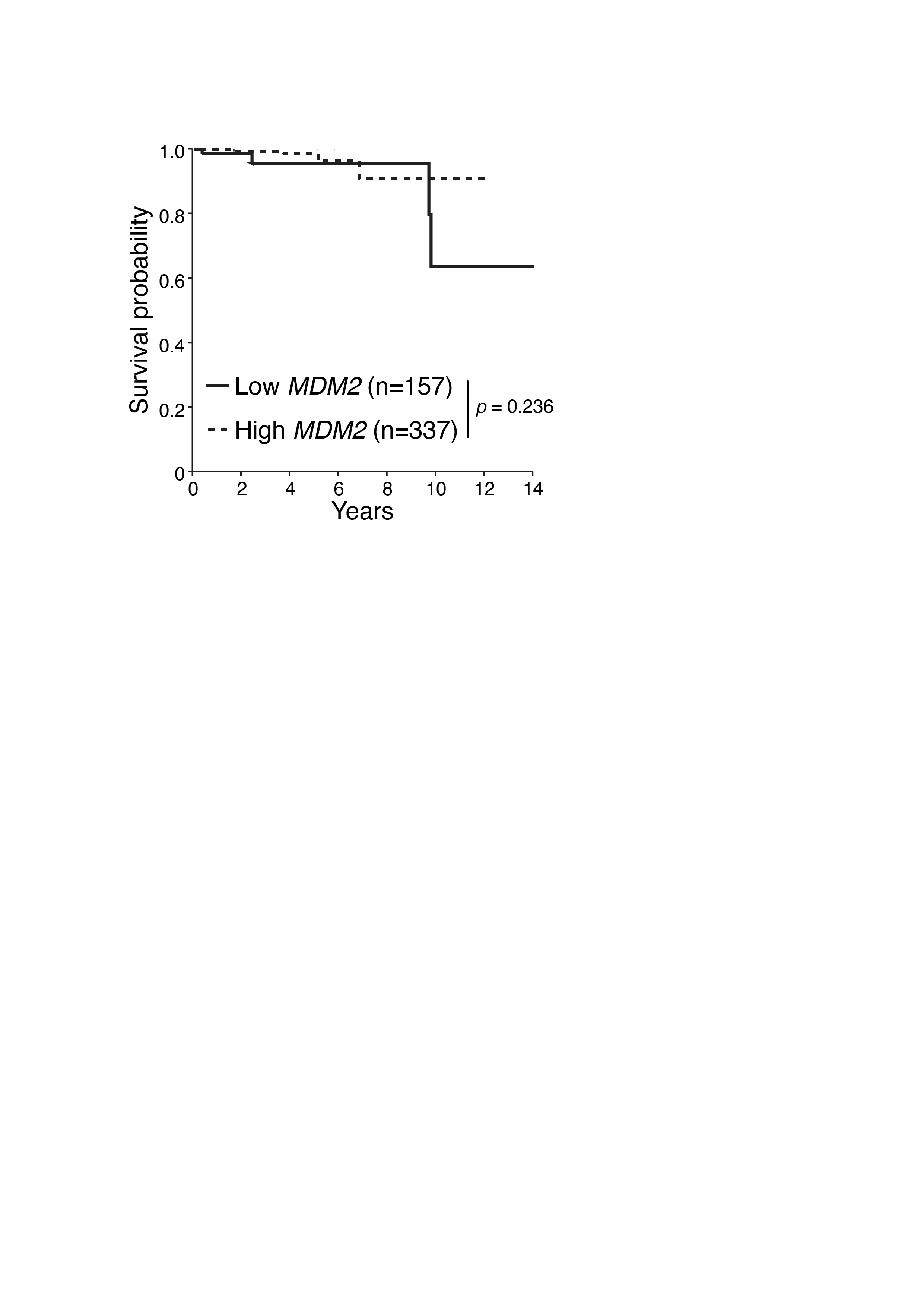


**Figure S2. *MDM2* levels and their impact on prostate cancer patient survival.** Kaplan-Meier plot for PC patients expressing either low or high *MDM2* mRNA levels as a function of survival probability. Statistical significance was calculated using Log-rank (Mantel-Cox) test.

**
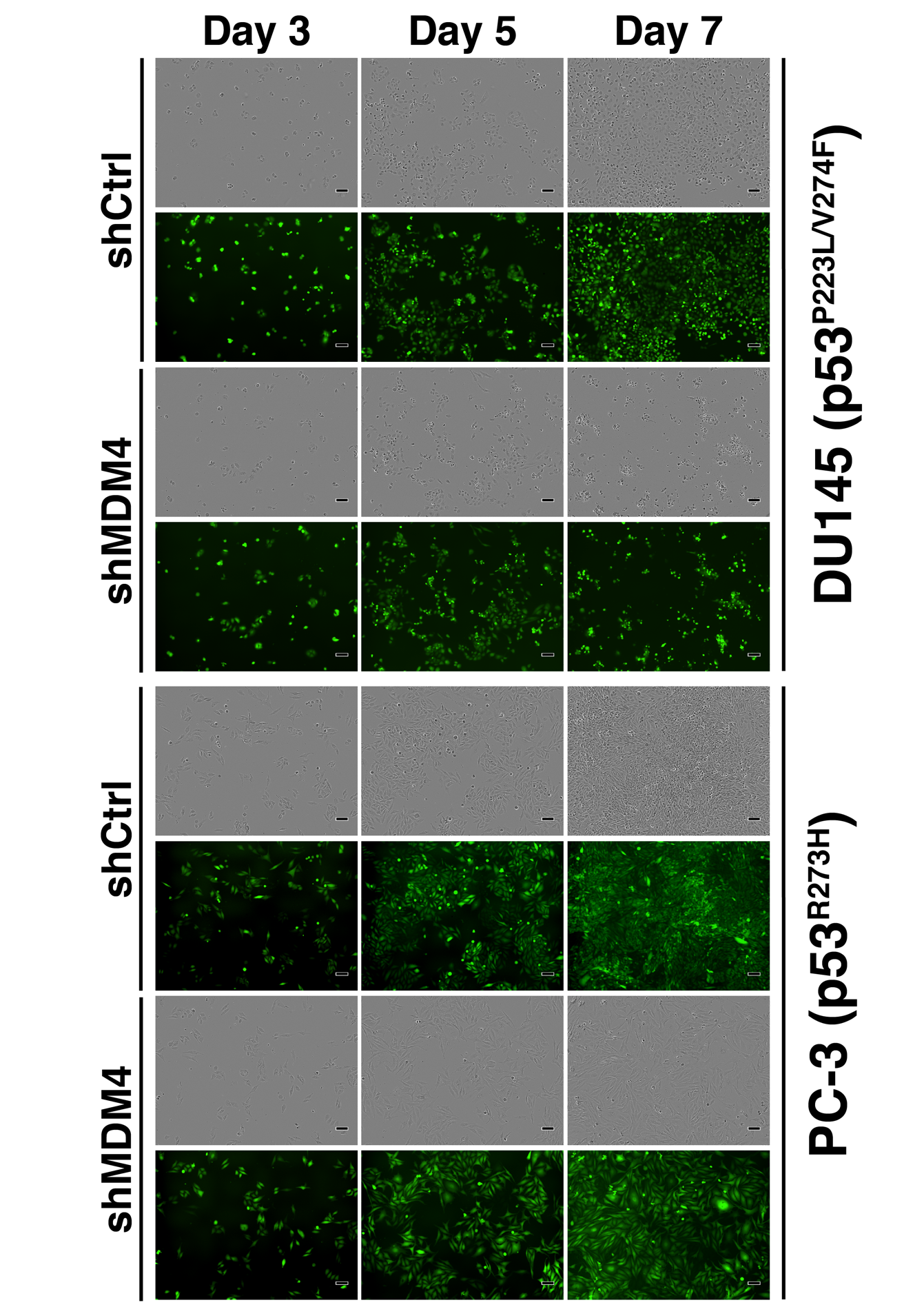
Figure S3. *MDM4* knockdown inhibited the growth of DU145 (p53^p223L/V274F^) and PC-3 (p53^R273H^).** Representative phase-contrast and fluorescence microscopy images of mutant p53 and GPF-tagged PC cell lines treated with Doxycycline (Doxy; 25ng/mL) over a period of 7 days. Scale bars indicate 100μm in all cases.

**
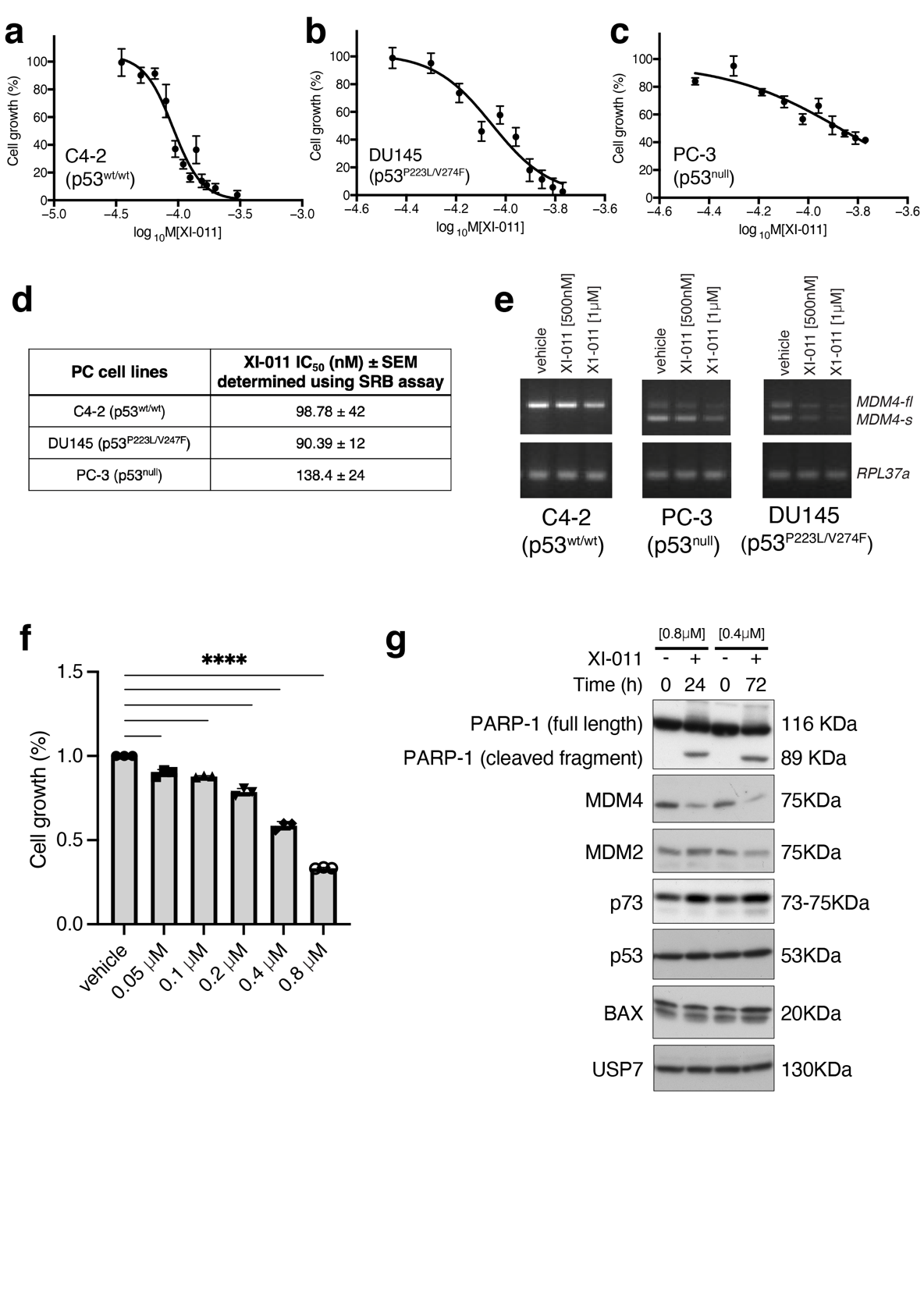
Figure S4. MDM4 targeting agent XI-011, inhibited the *in vitro* growth of prostate cancer cell lines. (a-c)** Three PC cell lines C4-2 (p53^wt/wt^), DU145 (p53^P223L/V274F^), and PC-3 (p53^null^) were treated with XI-011 for a period of 96 hours and the effects over cell numbers were assessed using SRB assay. **(d)** The table shows the XI-011 IC_50_ values determined for each PC cell line. **(e)** Reduction of *MDM4* levels in response to XI-011 after 24h was evident as demonstrated for C4-2 (p53^wt/wt^), DU145 (p53^P223L/V274F^) and PC-3 (p53^null^) using two different concentrations of XI-011 (500nM and 1uM). **(f)** The graph showing the relative survival of VCaP after XI-011 96 hours treatment assessed by CellTiter-Blue. Data shown as mean ± SEM of biological replicates (n=3). Statistical significance was calculated using ANOVA and Tukey’s tests (*p≤0.05, **p≤0.01, ***p≤0.001, ****p≤0.0001). **(g)** VCaP PC cells were treated with XI-011 and collected for exploring the protein expression levels using Western blot.

**
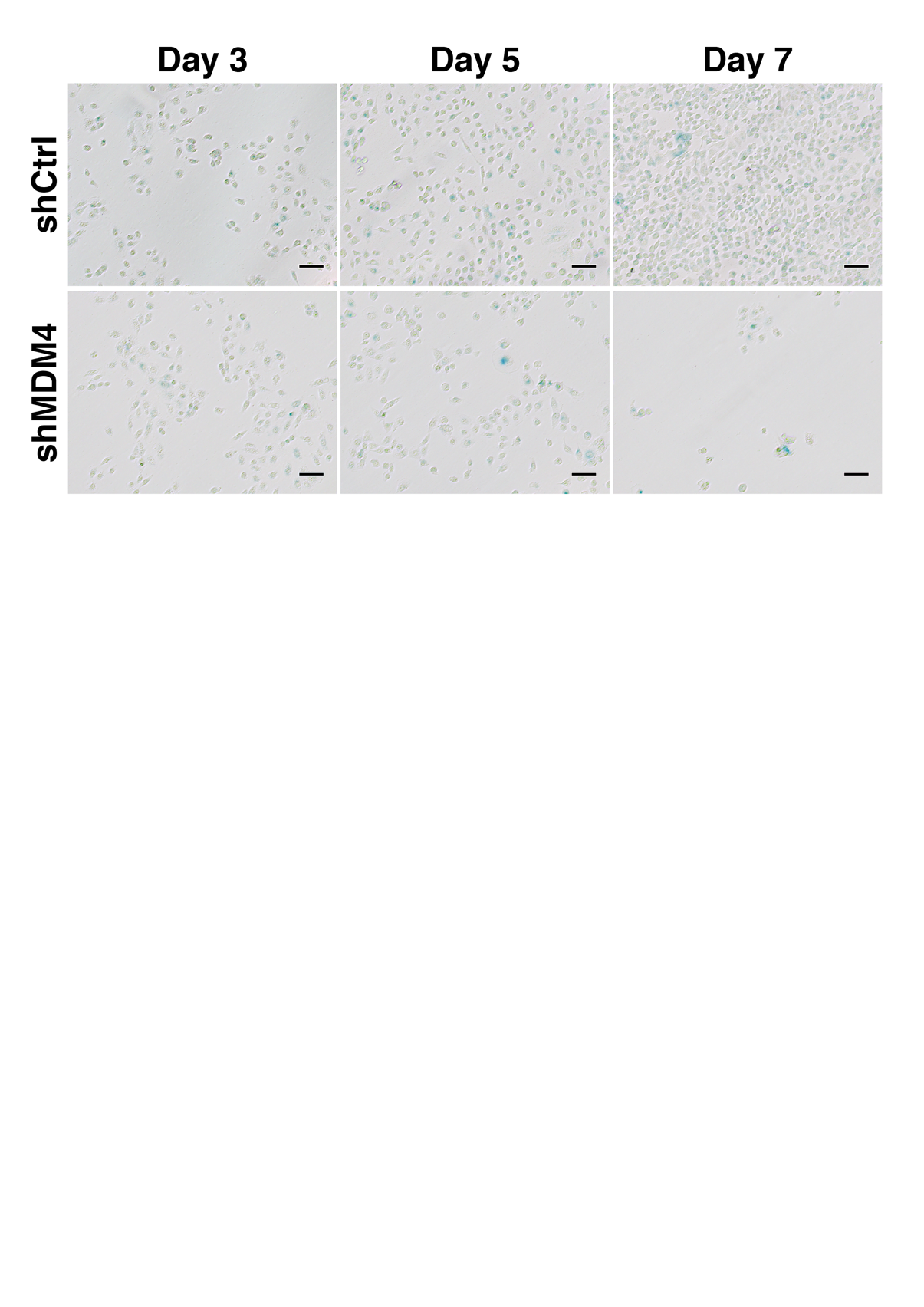
Figure S5. *MDM4* KD does not induce senescence in DU145 *in vitro*.** MDM4 inhibition does not cause senescence in DU145 as revealed by Senescence-associated β-galactosidase (SA-β-gal) staining at pH 6 on day 3, day 5, and day 7. SA-β-gal-positive cells stains in blue. Scale bars indicate 100μm.

**
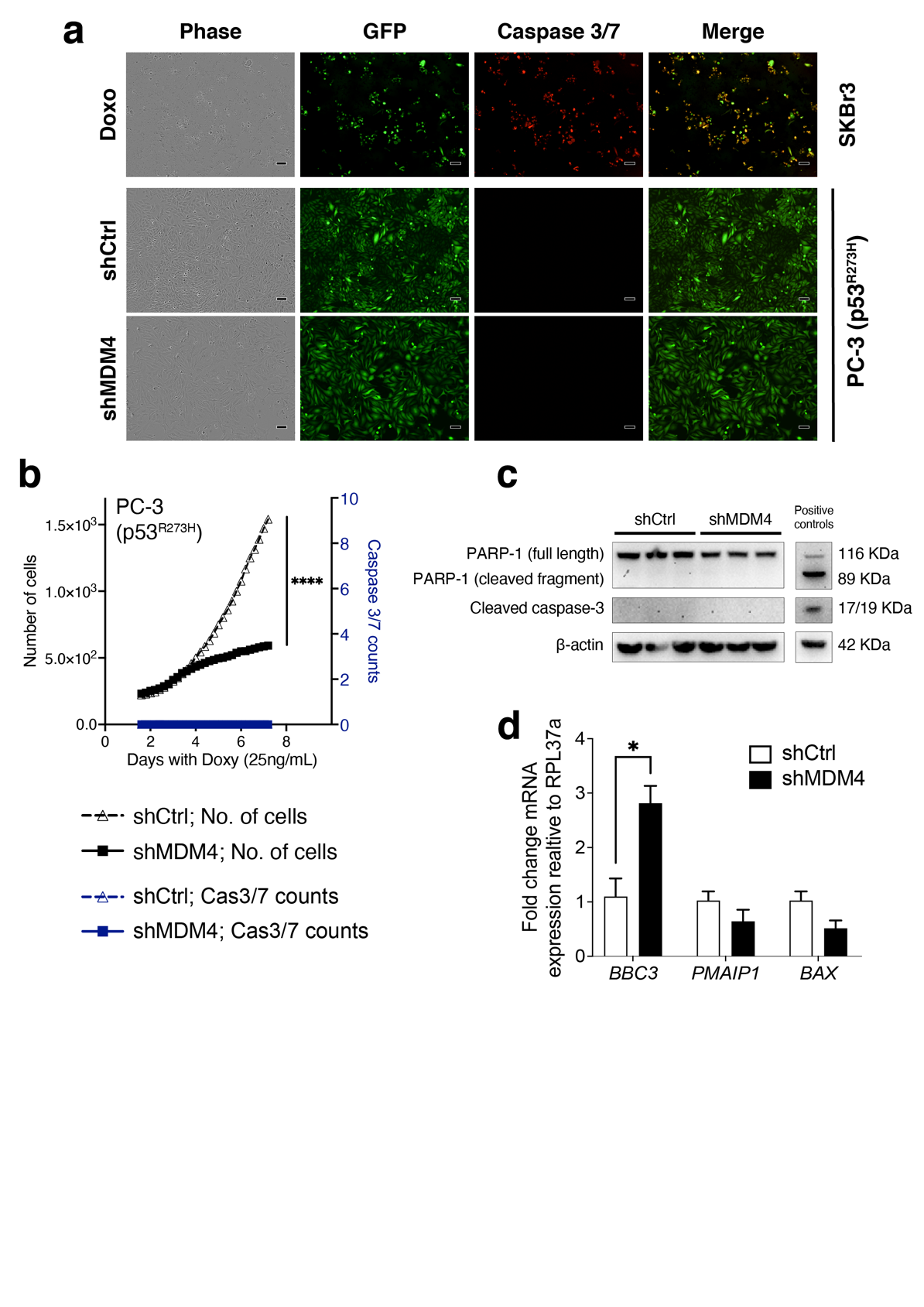
Figure S6. *MDM4* KD does not induce caspase 3 and caspase 7 activation in PC-3 (p53^R273H^) PC cell line.** SKBr3 cells (GFP tagged) were treated with 20µM of doxorubicin and used as apoptosis-positive control. Either shMDM4 or shCtrl expression was induced with Doxycycline (Doxy; 25ng/mL) in GPF-tagged PC-3 (p53^R273H^) for 7 days. On day 2, cells were treated with Red Incucyte® Caspase-3/7 Dye for detecting apoptosis. **(a)** Representative phase-contrast and fluorescence microscopy images; scale bars indicate 100μm. **(b)** Cell growth rate and kinetic activation of caspase-3/7 were monitored using the live-cell imaging Incucyte® system. **(c)** After MDM4 inhibition, protein was extracted on day 5. Activation of caspase 3 and PARP-1 was explored by Western blot. Each column corresponds to a biological replicate. **(d)** mRNA expression of apoptosis related genes was analysed by RT-qPCR after 5 days of treatment with Doxycycline. mRNA expression levels were normalised to the housekeeping gene *hRLP37a* and expressed relative to shCtrl. Data are shown as mean ± SEM of biological replicates (n=3-6). Statistical significance was calculated using a two-tailed student’s t-test (*p≤0.05, **p≤0.01, ***p≤0.001, ****p≤0.0001).

**
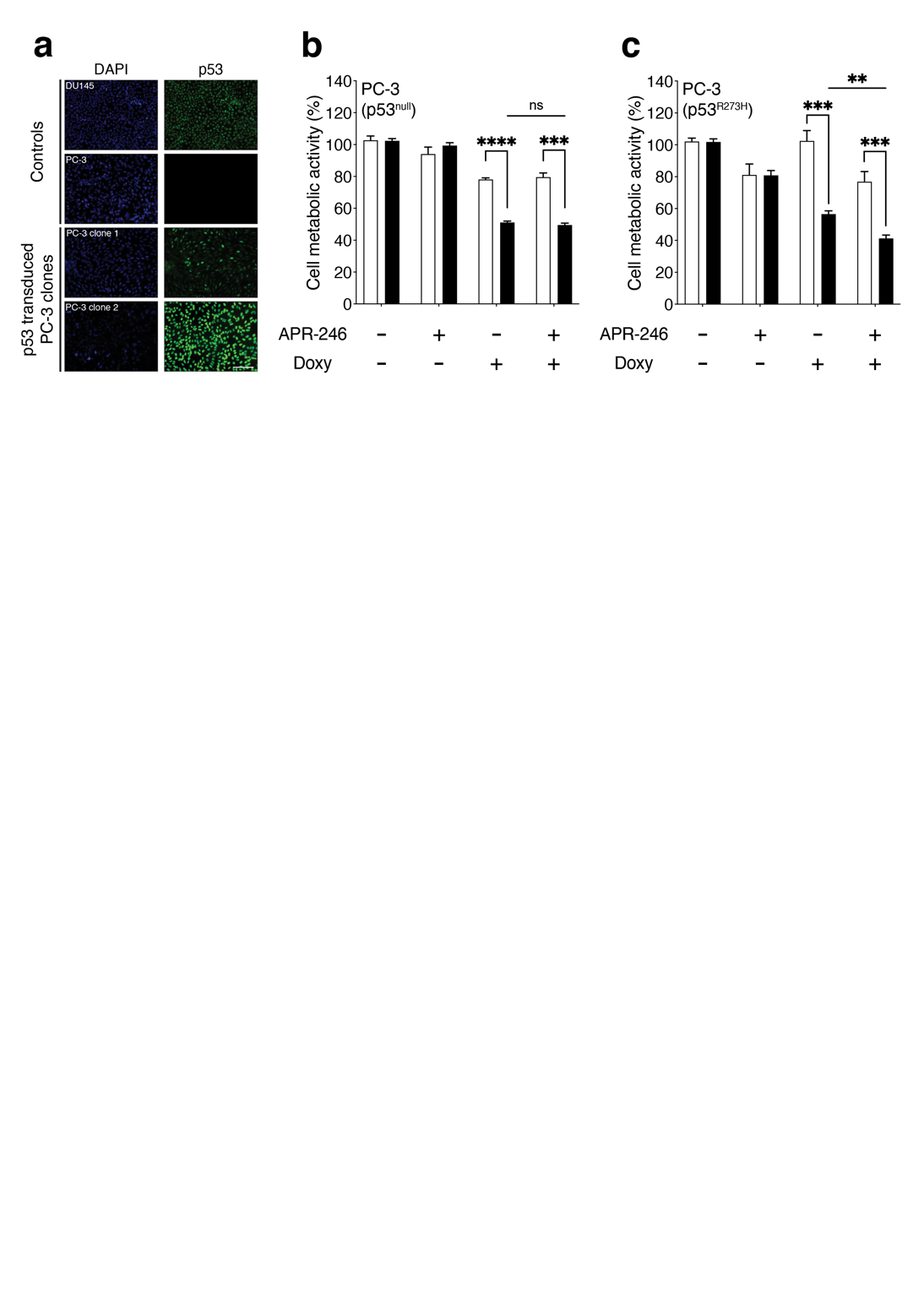
Figure S7. p53 immunofluorescence staining of PC-3 (p53^R273H^) and treatment efficacy of the combination treatment *MDM4* KD and eprenetapopt in PC-3 prostate cancer isogenic cell lines*.* (a)** Immunofluorescence staining of mutant p53 in PC-3 isogenic clones transduced with the missense p53^R273H^. DU145 and PC-3 (p53^null^) were used as controls. DAPI was used to stain the nuclei. Scale bar is 100μm. **(b & c)** To examine whether APR-246 increases the efficacy of MDM4 inhibition, PC-3 isogenic cell lines were treated either with IC_30_ of APR-246 alone or in combination with Doxycycline (Doxy; 25ng/mL) over a period of 5 days (see **Supplementary Table 2**). Treatment response was evaluated by assessing the suppression of cell growth using alamar blue assay. Data are shown as mean ± SEM of biological replicates (n=3). Statistical significance was calculated using a two-tailed student’s t-test (*p≤0.05, **p≤0.01, ***p≤0.001, ****p≤0.0001).

**
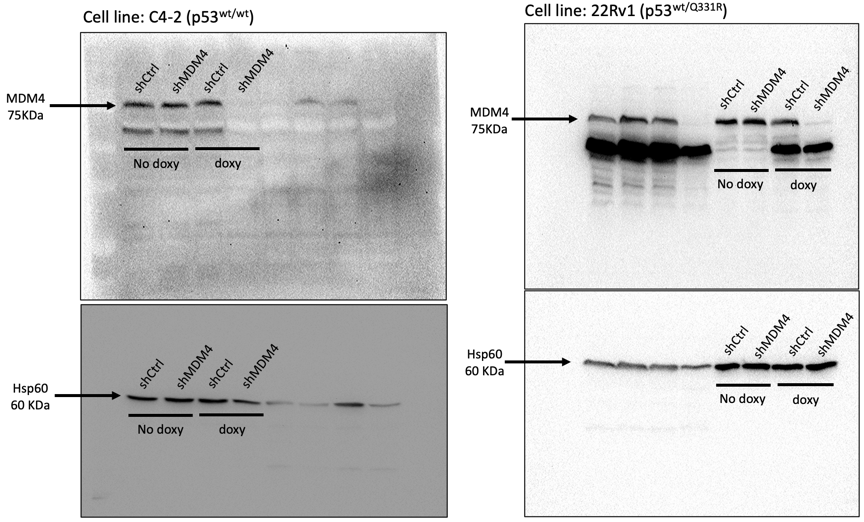
**

**
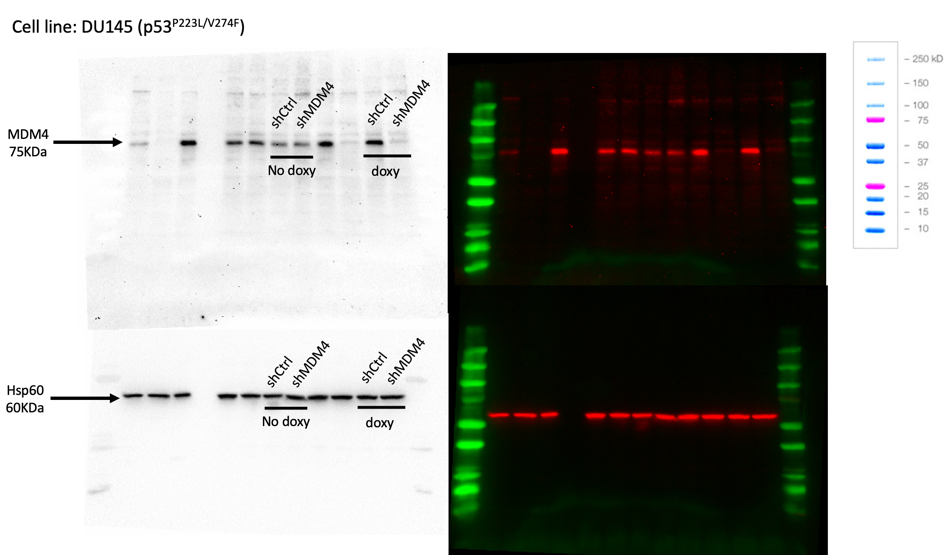

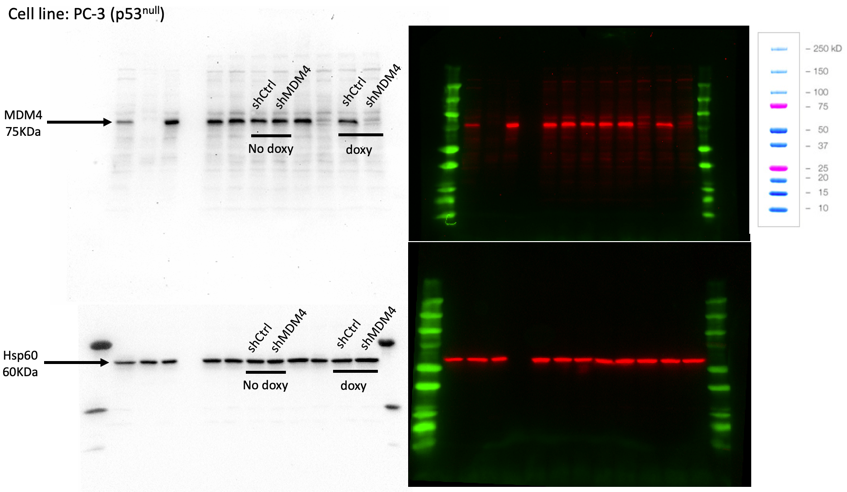
**

**
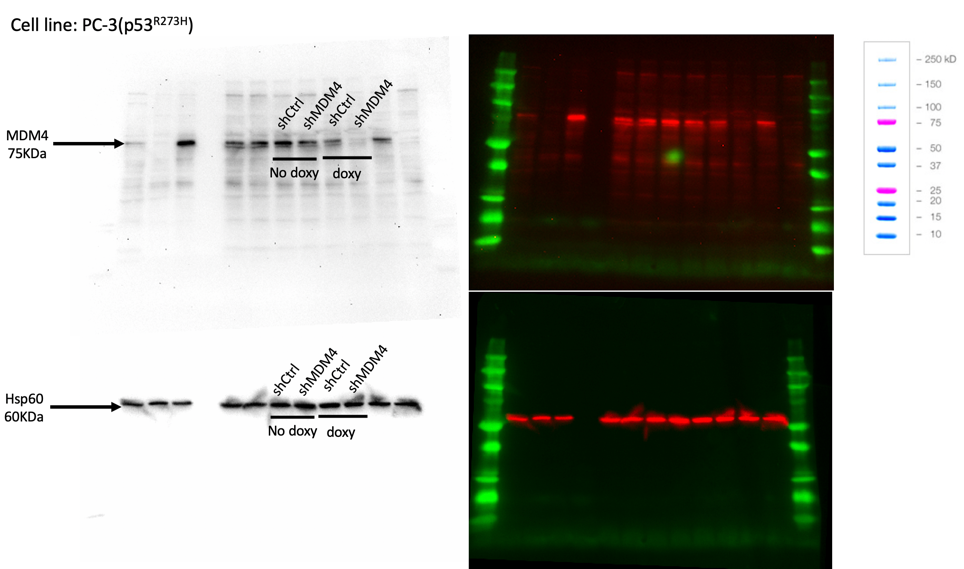
**

**Figure S8. Raw Western blot data of Figure 2**

**
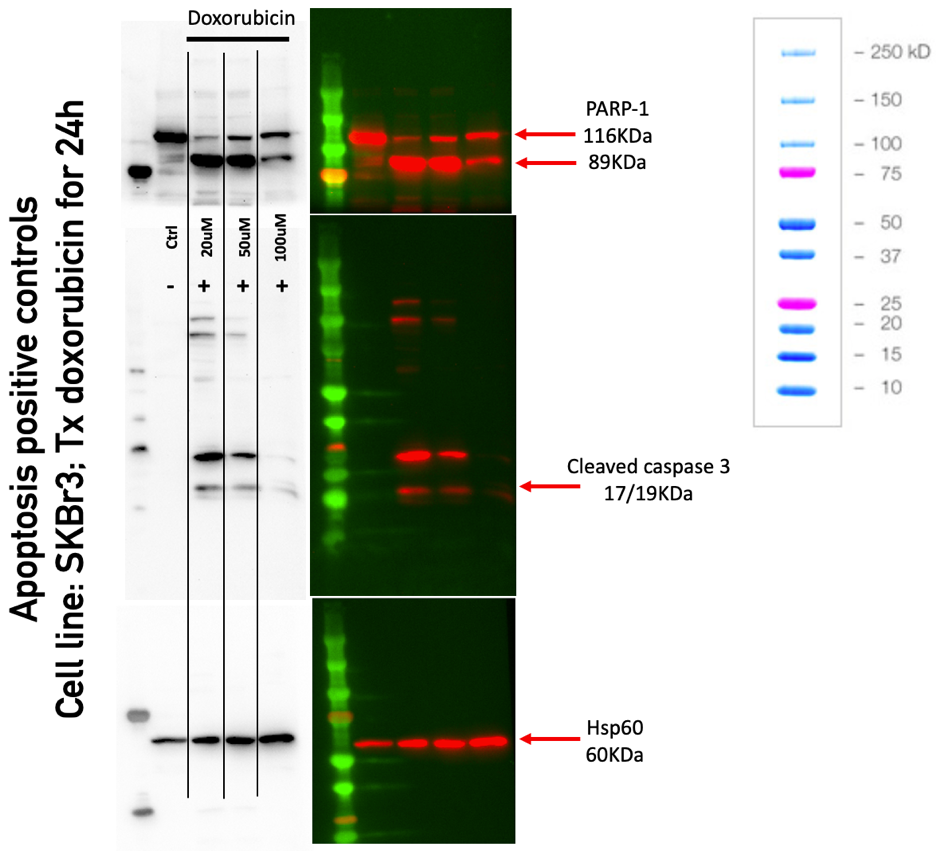
**

**Figure S9. Raw Western blot, apoptosis positive controls. SKBr3 treated with doxorubicin for 24h.**

**
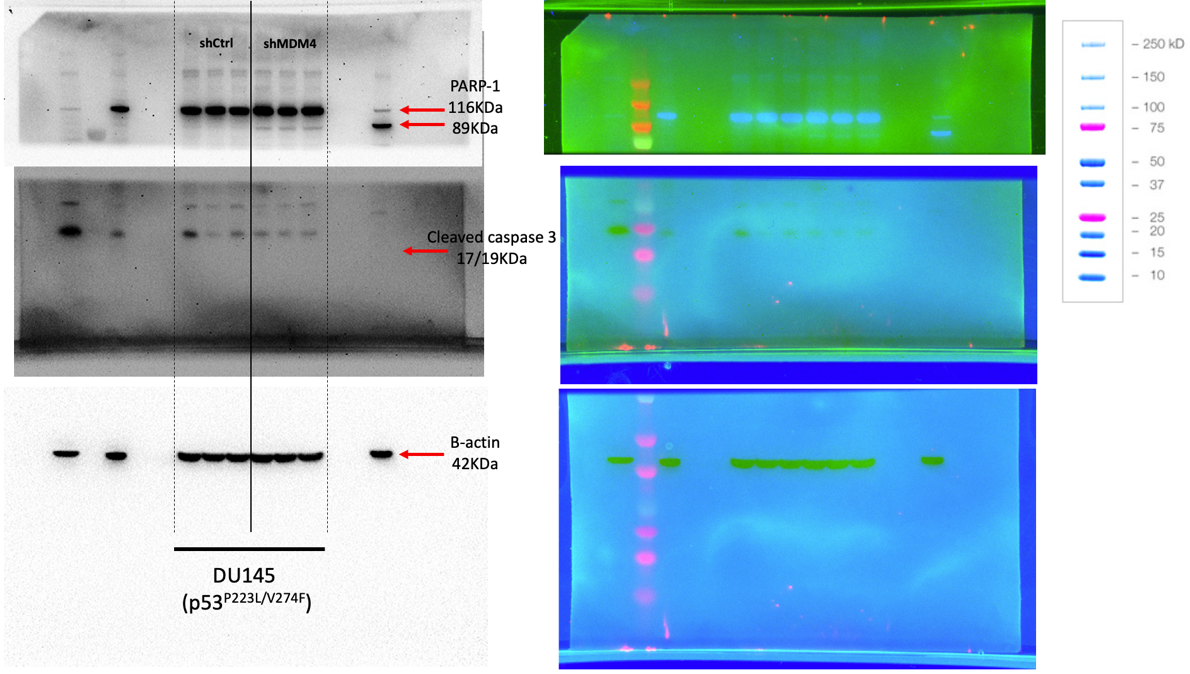
**

**Figure S10. Raw Western blot data of Figure 3.**

**
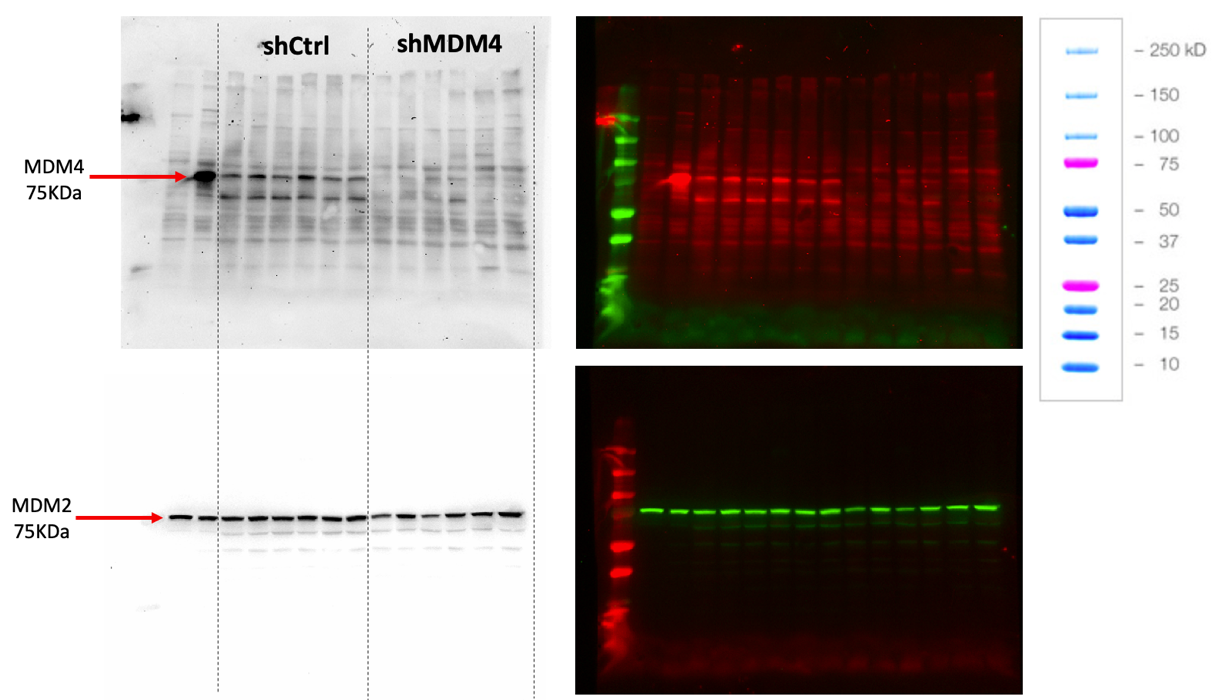
**

**
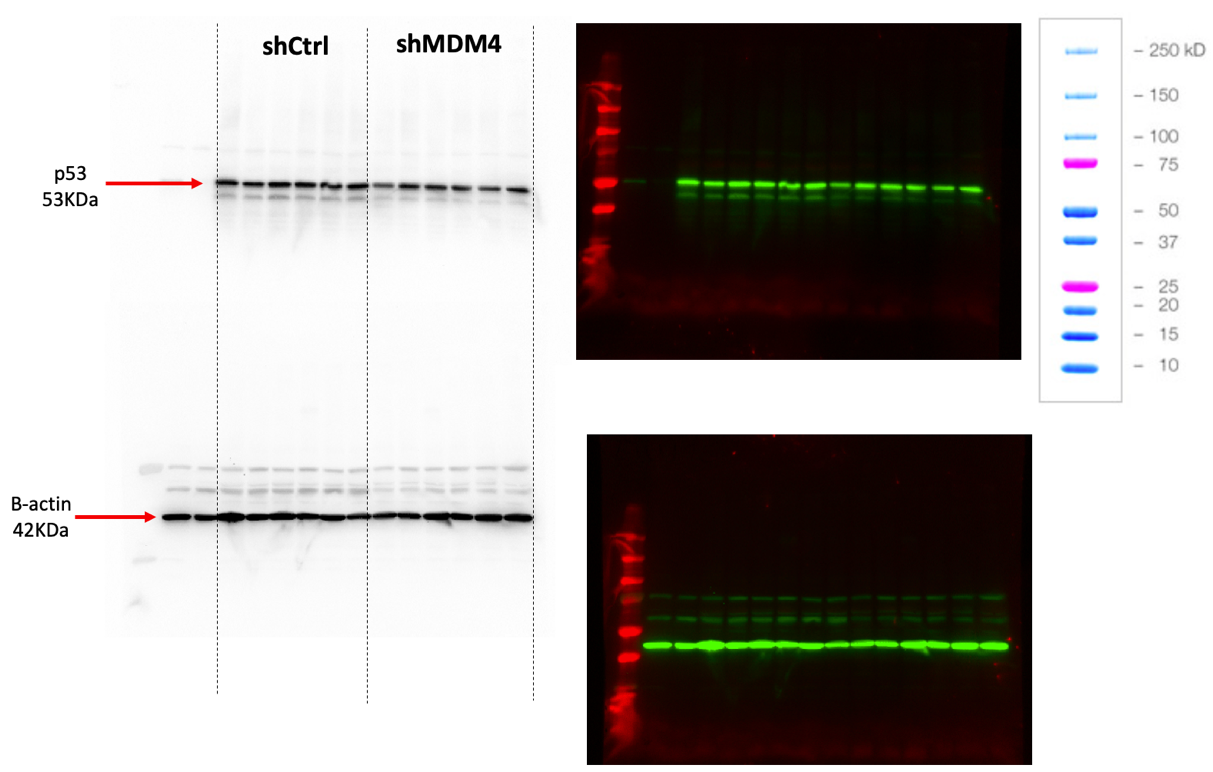
**

**Figure S11. Raw Western blot data of Figure 4.**

**
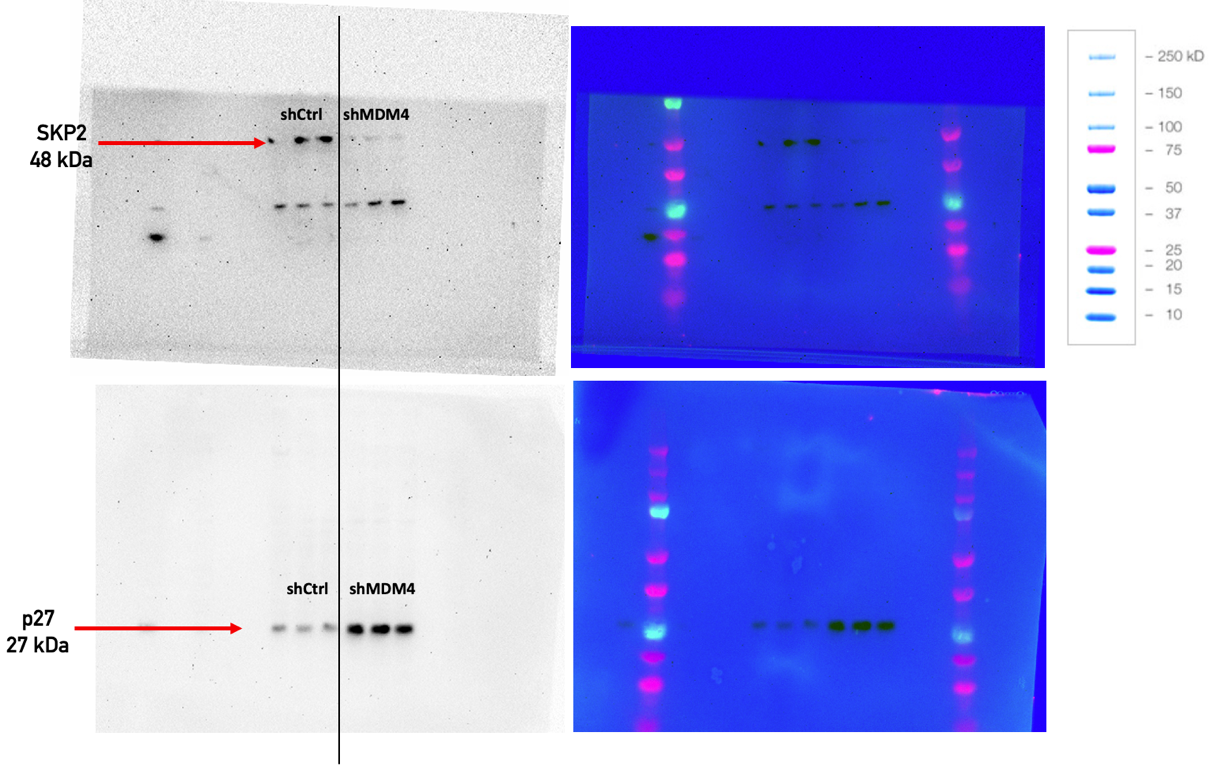

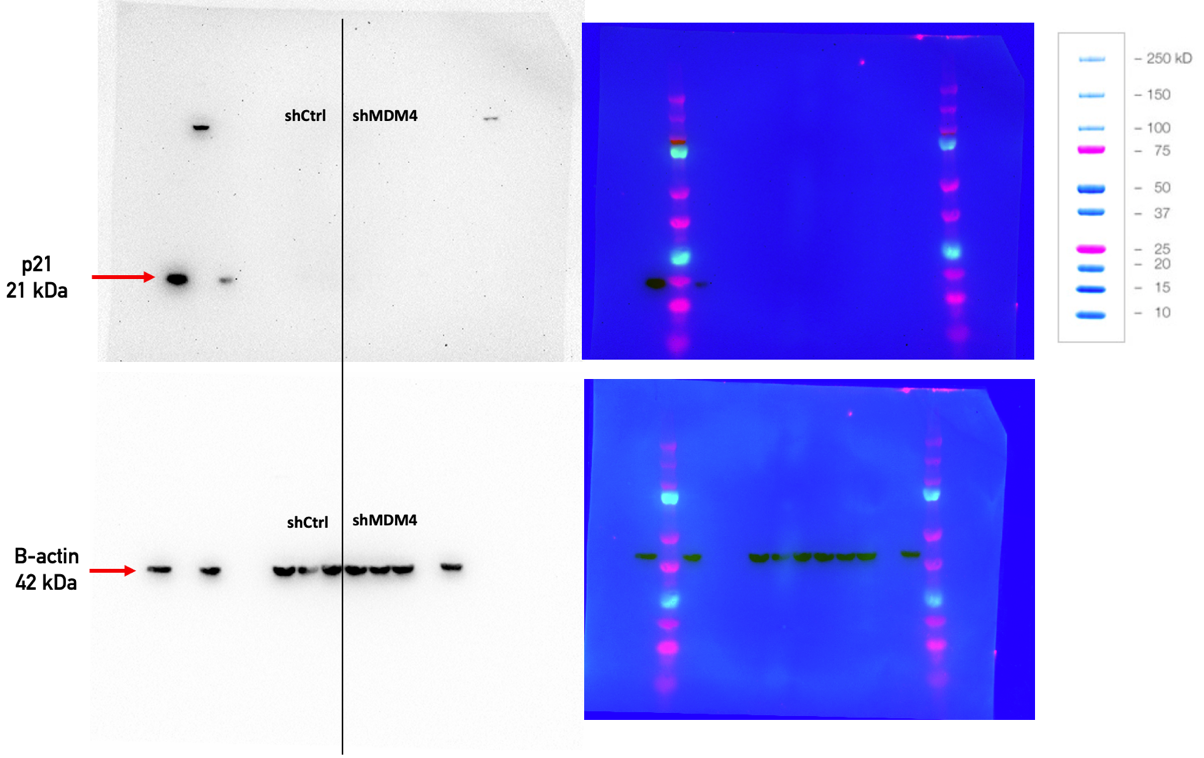
**

**Figure S12. Raw Western blot data of Figure 5.**

**
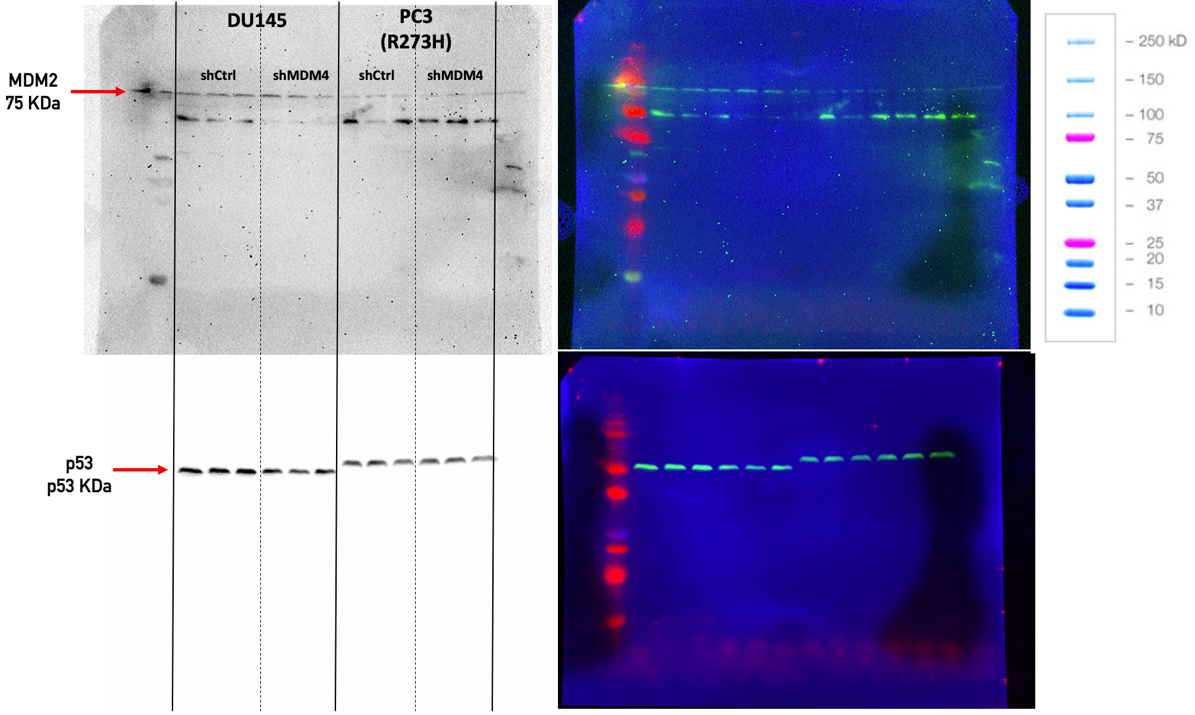
**

**
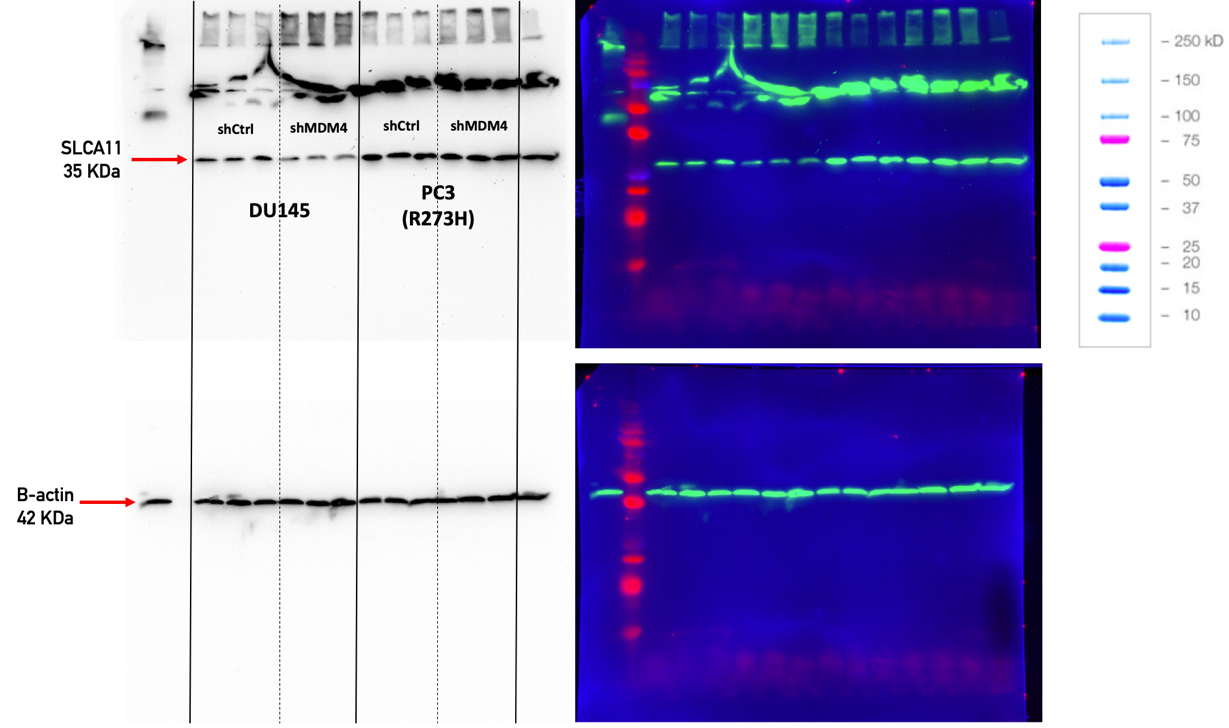
**

**Figure S13. Raw Western blot data of Figure 6.**

**
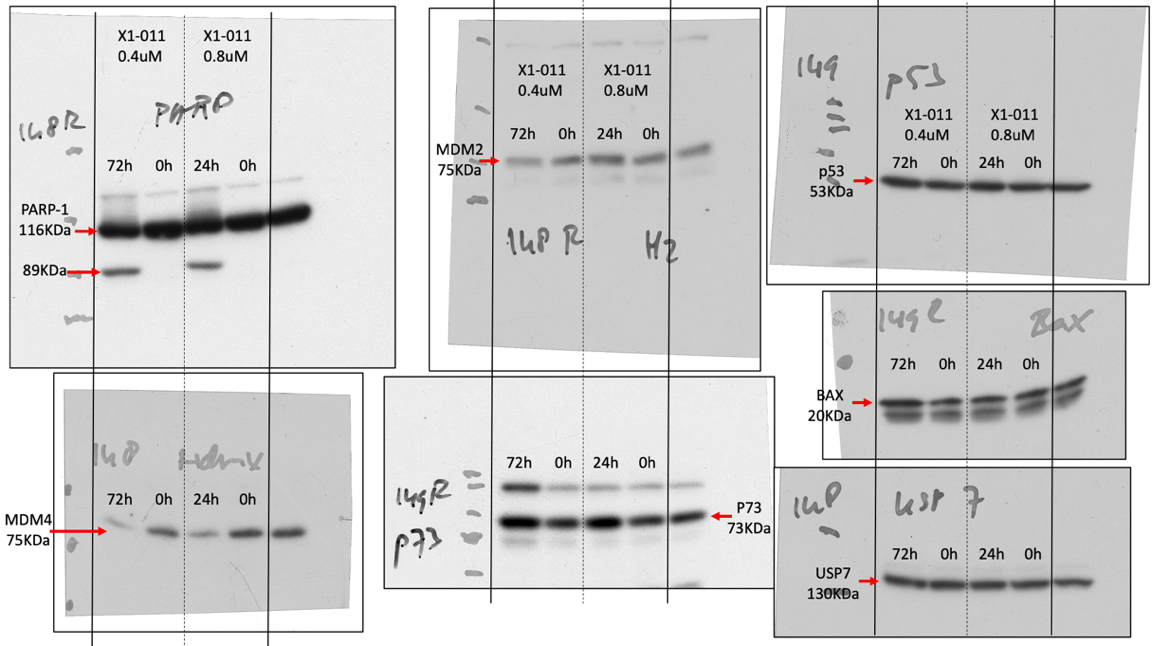
**

**Figure S14. Raw Western blot data of Figure S4.**

**
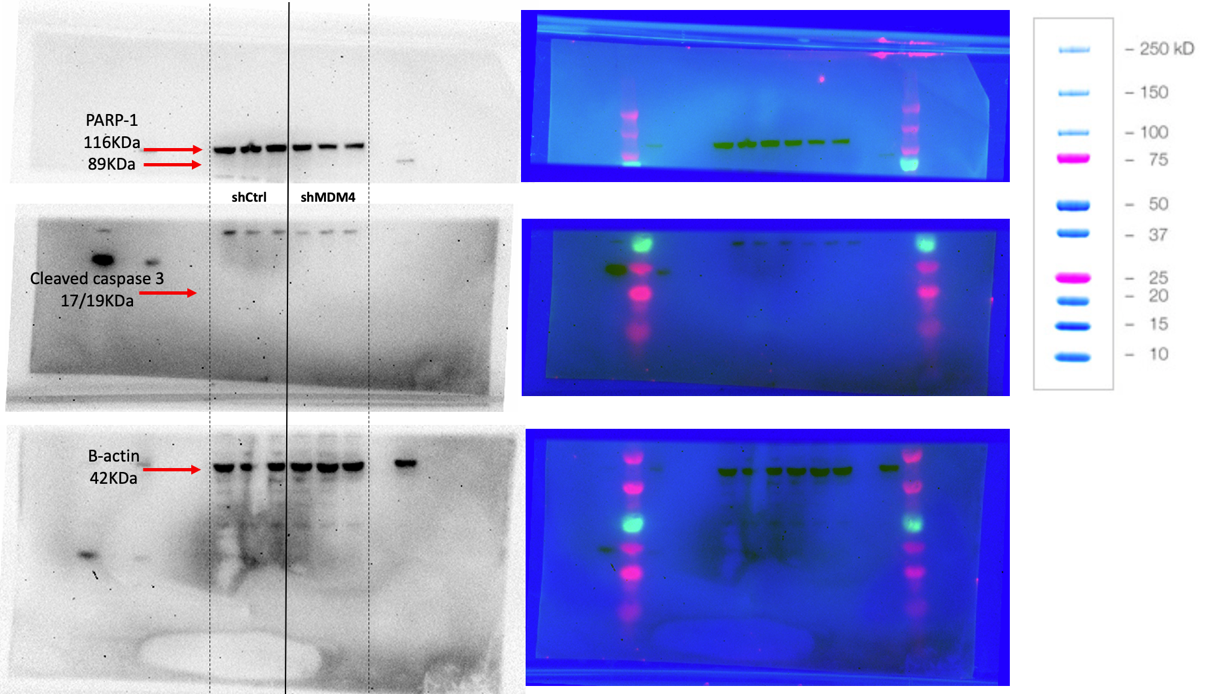
**

**Figure S15. Raw Western blot data of Figure S6.**

**Table S1.** PC patient sample immunohistochemistry (IHC) scoring

| **Patient** | **Slide** | **Tumour sample** | **IHC histoscore** | | |
| --- | --- | --- | --- | --- | --- |
|  |  |  | **p53** | **MDM2** | **MDM4** |
| Patient 1 | 216 | Para-aortic lymph nodes (right) | 4 | 0 | 7 |
|  | 222 | Prostate - apex left side | 4 | 0 | 7 |
|  | 226 | Dural base skull | 4 | 0 | 7 |
| Patient 2 | 327 | Prostate tissue - neck of bladder | 0 | 5 | 7 |
|  | 331 | Porta-hepatic lymph node | 0 | 2 | 7 |
|  | 334 | Liver: right lobe A | 0 | 3 | 7 |
| Patient 3 | 442 | Left para-aortic lymph node track | 4 | 6 | 6 |
|  | 444 | Bladder base #2 | 5 | 3 | 7 |
|  | 447 | T4/T5 vertebrae | 4 | 7 | 7 |
| Patient 4 | 297 | Peri-vertebral tumour | 7 | 1 | 7 |
|  | 305 | Liver: right lobe deposit A | 5 | 1 | 6 |
| Patient 5 | 430 | Thoracic vertebrae | 5 | 5 | 7 |
|  | 431 | lymph node - portal | 5 | 2 | 7 |
|  | 433 | Liver: right lobe nodule 1 | 5 | 5 | 7 |

**Table S2.** Epenetapopt IC_50_ values for prostate cancer (PC) cell lines

| **PC cell lines** | **Epenetapopt (APR-246) IC_50_ ± SEM determined using Alamar Blue assay** |
| --- | --- |
| R22v1 (^p53wt/Q331R^) | 3.5 ± 0.39 |
| DU145 (p53^P223L/V247F^) | 16.3 ± 1.93 |
| PC-3 parental (p53^null^) | 32.5 ± 2.90 |
| PC-3 clone 1 (p53^R273H^) | 16.3 ± 2.07 |
| PC-3 clone 2 (p53^R273H^) | 16.4 ± 1.12 |
